# Activation and inhibition of sirtuins: From bench to bedside

**DOI:** 10.1002/med.22076

**Published:** 2024-08-31

**Authors:** Francesco Fiorentino, Emanuele Fabbrizi, Antonello Mai, Dante Rotili

**Affiliations:** ^1^ Department of Drug Chemistry and Technologies Sapienza University of Rome Rome Italy; ^2^ Pasteur Institute, Cenci‐Bolognetti Foundation Sapienza University of Rome Rome Italy

**Keywords:** cancer, drug discovery, metabolism, protein lysine deacylation, sirtuins

## Abstract

The sirtuin family comprises seven NAD^+^‐dependent enzymes which catalyze protein lysine deacylation and mono ADP‐ribosylation. Sirtuins act as central regulators of genomic stability and gene expression and control key processes, including energetic metabolism, cell cycle, differentiation, apoptosis, and aging. As a result, all sirtuins play critical roles in cellular homeostasis and organism wellness, and their dysregulation has been linked to metabolic, cardiovascular, and neurological diseases. Furthermore, sirtuins have shown dichotomous roles in cancer, acting as context‐dependent tumor suppressors or promoters. Given their central role in different cellular processes, sirtuins have attracted increasing research interest aimed at developing both activators and inhibitors. Indeed, sirtuin modulation may have therapeutic effects in many age‐related diseases, including diabetes, cardiovascular and neurodegenerative disorders, and cancer. Moreover, isoform selective modulators may increase our knowledge of sirtuin biology and aid to develop better therapies. Through this review, we provide critical insights into sirtuin pharmacology and illustrate their enzymatic activities and biological functions. Furthermore, we outline the most relevant sirtuin modulators in terms of their modes of action, structure–activity relationships, pharmacological effects, and clinical applications.

**Abbreviations**
ACAD: acyl‐CoA dehydrogenase
ACAT1: acetyl‐CoA acetyltransferase 1
AceCS2: acetyl‐CoA synthetase 2
ACOX1: acyl‐CoA oxidase 1
ACS: acetyl‐CoA synthetase 2
AD: Alzheimer's disease
ADP: adenosine diphosphate
AI: artificial intelligence
ALDH1A1: aldehyde dehydrogenase 1A1
ALL: acute lymphoblastic leukemia
AMC: 7‐amino‐4‐methylcoumarin
AML: acute myeloid leukemia
AMPK: 5’ AMP‐activated protein kinase
AP‐1: activator protein 1
ATM: ataxia‐telangiectasia mutated
ATP: adenosine triphosphate
BACE1: β‐secretase 1
BAT: brown adipose tissue
Bax: Bcl2‐associated X protein
C_max_
: maximal plasma concentration
cAMP: cyclic adenosine monophosphate
Cbz: carbobenzyloxy
CD31: cluster of differentiation 1
CETSA: cellular thermal shift assay
CETSA: cellular thermal shift assay
CLP: cecal ligation/perforation
CML: chronic myeloid leukemia
CNS: central nervous system
CPS1: carbamoyl phosphate synthetase 1
CRC: colorectal cancer
CSCs: cancer stem cells
DHP: dihydropyridine
DHP: dihydropyridine
DLBCL: diffuse large B‐cell lymphoma
DSB: double strand break
E2F1: E2F transcription factor 1
ELF3: early flowering 3
EMT: epithelial‐mesenchymal transition
ER: endoplasmic reticulum
ETC: electron transport chain
FBXO7: F‐box‐only protein 7
FdL: Fluor‐de‐Lys
FOXO: forkhead box O
G6PC: glucose‐6‐phosphatase catalytic subunit
GADPH: glyceraldehyde 3‐phosphate dehydrogenase
GBM: glioblastoma multiforme
GCDH: glutaryl‐CoA‐dehydrogenase
GDH: glutamate dehydrogenase
GLUD1: glutamate dehydrogenase 1
GOT1: glutamic‐oxaloacetic transaminase 1
GSH: reduced glutathione
H2AX: H2A histone family member X
HAT: histone acetyltransferase
HCC: hepatocellular carcinoma
HD: Huntington's disease
HDAC: histone deacetylase
HDL: high‐density lipoprotein
HDX‐MS: hydrogen/deuterium exchange mass spectrometry
HIF‐1α: hypoxia‐inducible factor‐1α
HMG: 3‐hydroxy‐3‐methyl‐glutaryl
HMGCS2: 3‐hydroxy‐3‐methyl‐glutaryl‐CoA synthase 2
HPMEC: human pulmonary lung microvascular endothelial cells
HT7: HaloTag 7
HUVEC: human umbilical venous endothelial cell
IC_50_
: half maximal inhibitory concentration
ICAM: Intercellular Adhesion Molecule 1
IDH: isocitrate dehydrogenase
IGF: insulin‐like growth factor
IGF2BP2: insulin‐like growth factor 2 mRNA‐binding protein 2
IL: interleukin
IPF: idiopathic pulmonary fibrosis
ITC: isothermal titration calorimetry
ITDRF‐CETSA: isothermal dose–response fingerprinting cellular thermal shift assay
JAK3: Janus Kinase 3
KLF15: Kruppel‐like factor 15
LCAD: long chain acyl‐CoA dehydrogenase
LDHA: lactate dehydrogenase A
LDL: low‐density lipoprotein
LINE‐1: long interspersed element‐1
LKB1: liver kinase B1
LPS: lipopolysaccharide
MAO‐B: monoamine oxidase B
MAPK: mitogen‐activated protein kinase
MCAD: medium‐chain acyl‐CoA dehydrogenase
Mcl‐1: myeloid leukemia cell differentiation protein 1
MD: molecular dynamics
MEF: mouse embryonic fibroblast
MMP9: matrix metallopeptidase 9
MPP^+^
: 1‐methyl‐4‐phenylpyridinium
MPTP: 1‐methyl‐4‐phenyl‐1,2,3,6‐tetrahydropyridine
MS: mass spectrometry
mTOR: mammalian target of rapamycin
NAD^+^
: nicotinamide adenine dinucleotide
NADPH: nicotinamide adenine dinucleotide phosphate
NBS1: Nijmegen Breakage Syndrome‐1
NDUFA9: NADH dehydrogenase [ubiquinone] 1 alpha subcomplex subunit 9
NFATc2: nuclear factor of activated T‐cells cytoplasmatic 2
NF‐κB: nuclear factor kappa B
NME4: nucleoside diphosphate kinase
Nrf2: nuclear factor erythroid 2–related factor 2
NSCLC: non‐small cell lung cancer
NSP14: nonstructural viral protein 14
OSCC: oral squamous cell carcinoma
OTC: ornithine transcarbamylase
p70S6K1: ribosomal protein S6 kinase beta‐1
PABPN1: polyadenylate‐binding protein nuclear 1
PARP: poly(ADP‐ribose) polymerase
PD: Parkinson's disease
PDAC: pancreatic ductal adenocarcinoma
PDH: pyruvate dehydrogenase
PDHA1: pyruvate dehydrogenase subunit E1α
PDO: patient‐derived organoid
PDX: patient‐derived xenograft
PFKFB3: 6‐phosphofructo‐2‐kinase/fructose‐2,6‐bisphosphotase 3
PGK1: phosphoglycerate kinase 1
PKA: protein kinase A
PKM2: pyruvate kinase M2
pRB: retinoblastoma protein
PRMT5: protein arginine methyltransferase 5
PTM: posttranslation modification
RaPID: random nonstandard peptides integrated discovery
ROS: reactive oxygen species
RUNX2: Runt‐related transcription factor 2
SAMDI‐MS: self‐assembled monolayer desorption/ionization mass spectrometry
SAMHD1: sterile alpha motif and HD domain‐containing protein 1
SAR: structure–activity relationships
SCF: SKP1‐Cullin‐1‐F‐box
SDH: succinate dehydrogenase
SHMT2: serine hydroxy methyltransferase 2
Sir2: silent information regulator 2
SIRT: sirtuin
SIRTa: sirtuin activator(s)
SIRTi: sirtuin inhibitor(s)
SLC39A8: Solute Carrier Family 39 Member 8
SLE: systemic lupus erythematosus
SMAD4: mothers against decapentaplegic homolog 4
SOD: superoxide dismutase
Sosbo: sirtuin one selective benzoxazines
SPR: surface plasmon resonance
STAC: small molecule sirtuin‐activating compound
STAT3: signal transducer and activator of transcription
TAMRA: tetramethylrhodamine
TCA: tricarboxylic acid
Th: T‐helper
TIGAR: TP53‐induced glycolysis and apoptosis regulator
TNBC: triple negative breast cancer
TNFSF4: tumor necrosis factor superfamily member
TNF‐α: tumor necrosis factor α
TPP: triphenylphosphonium
TSA: trichostatin A
UCP1: uncoupling protein 1
VCAM: vascular cell adhesion protein 1
VDAC3: voltage dependent anion channel 3
VEGF‐A: vascular endothelial growth factor A
VMSC: vascular smooth muscle cell
WDR77: WD repeat domain 77
Wip1: wild‐type p53‐induced phosphatase 1
YY1: Yin Yang 1 transcription factor

## 1 INTRODUCTION

**Figure 1:**
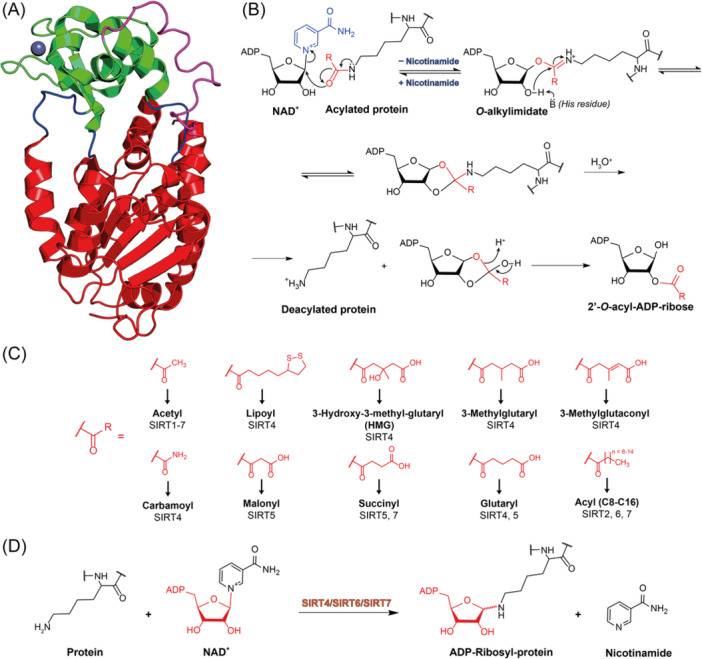
(A) X‐ray crystal structure of *H. sapiens* SIRT2 (PDB ID: 1J8F). The Rossmann‐fold domain is depicted in red, the Zn^2+^‐binding domain in green, the key loops in blue, the cofactor binding loop in magenta, and the Zn^2+^ is represented as a dark sphere. (B) Schematic representation of the mechanism of SIRT‐catalyzed deacylation. (C) Overview of the various acyl substrates of the different sirtuins. (D) Schematic representation of the mono‐ADP‐ribosylation reaction catalyzed by SIRT4, SIRT6, and SIRT7. [Color figure can be viewed at wileyonlinelibrary.com]

Histone lysine acetylation is a posttranslation modification (PTM) catalyzed by histone acetyltransferases (HATs), while the opposite reaction is mediated by histone deacetylases (HDACs).^1^, ^2^, ^3^ HDACs are divided into two groups based on the presence of a conserved deacetylase domain and their reliance on specific cofactors: the Zn^2+^‐dependent HDACs and sirtuins (SIRTs). According to sequence similarities to yeast deacetylases, the Zn^2+^‐dependent HDACs are subdivided into three classes: class I (HDAC1‐3, 8), class II (HDAC4‐7, 9, 10), and class IV (HDAC11).^1^, ^4^, ^5^ Depending on the composition of their domains, class II HDACs are divided into class IIa and class IIb. Sirtuins require nicotinamide adenine dinucleotide (NAD^+^) as co‐substrate for catalysis (Figure 1A) and present different structural features, thereby being classified as class III HDACs.^6^

Given the involvement of mammalian SIRTs in many critical functions for cellular and organism homeostasis, the alteration of their activity is connected to various pathologies such as cancer, neurodegenerative diseases, cardiovascular disorders, and metabolic alterations.^7^, ^8^, ^9^ SIRTs have also been identified as a possible target for the development of antiparasitic treatments given their key functions in parasite proliferation, survival, and host response.^10^, ^11^, ^12^, ^13^, ^14^, ^15^

The sirtuin family currently consists of seven isoforms (SIRT1 to SIRT7) sharing an evolutionarily conserved catalytic site of roughly 275 residues, while diverging in size and sequence of their *N*‐ and *C*‐terminal domains. For instance, SIRT4 and 5 only have a small mitochondrial‐localization *N*‐terminal portion while they lack the *C*‐terminal domain.^16^ The shared catalytic region includes a Rossmann‐fold and a small Zn^2+^‐binding domain, which surround a groove that contains the binding sites for both substrate and NAD^+^.^16^ The Rossmann‐fold domain contains a β‐sheet made of six parallel β‐strands surrounded by a varying number of α‐helices, depending on the SIRT isoform (Figure 1A). This organization is characteristic of NAD^+^/NADH binding proteins and indeed contains a conserved G‐X‐G motif that is essential for phosphate interaction, a NAD^+^ binding pocket, and charged amino acids that increase the affinity for the ribose portion. Two insertions arising from the Rossmann‐fold domain are packed to produce a single globular domain. One of these insertions binds Zn^2+^ through four conserved cysteine residues and has only a structural function (Figure 1A).^17^ The Rossmann‐fold and Zn^2+^‐binding domains are brought closer to each other upon substrate binding, which interacts with two adjacent loops via β‐sheet‐like interactions. Specifically, one loop belongs to the Rossman‐fold domain and the other consists of a loop containing the highly conserved F‐G‐E‐X‐L motif. These interactions contribute to the conformational change of the enzyme from an open to a close conformation leading to the so‐called β‐staple. This shift promotes binding of NAD^+^ and accommodation of nicotinamide in a binding cleft called C‐pocket, proximal to the acyl‐lysine binding site. Here, the ε‐nitrogen of the substrate lysine engages in a hydrogen bond with a conserved valine and the acyl group forms van der Waals interactions with specific residues.^18^

SIRTs share a similar deacylation mechanism, shown in Figure 1B.^19^, ^20^ Following the binding of the acylated substrate to the enzyme, the carbonyl oxygen of the acyl group attacks the ribose at the C1ʹ position, leading to the displacement of nicotinamide and yielding the *O*‐alkylamidate intermediate (Figure 1B). Then, a conserved histidine acts as a general base and leads to deprotonation of 2ʹ‐OH, which in turn attacks the imine carbon of the *O*‐alkylimidate, producing the C1ʹ/C2ʹ cyclic intermediate, which is finally hydrolyzed, yielding the deacylated protein and 2ʹ‐*O*‐acyl‐ADP‐ribose as reaction products.

Small changes in the binding site of each SIRT isoform, along with differences at the *N‐* and *C‐* termini, affect substrate specificity and cellular localization, along with the interaction with modulators and other proteins.^21^ Indeed, the 7 mammalian SIRTs regulate the acylation state of a broad range of protein substrates (Figure 1C,D). Specifically, SIRT1‐3 possess a prevalent protein lysine deacetylase activity, although SIRT2 was also shown to possess demyristoylase activity.^22^, ^23^, ^24^ SIRT4 possesses deacetylase, decarbamylase, lipoamidase, and mono‐ADP‐ribosyltransferase activities and also catalyzes the removal of 3‐hydroxy‐3‐methyl‐glutaryl (HMG) moieties from protein lysine residues.^25^, ^26^ SIRT5 has a weak deacetylase activity and prefers negatively‐charged acyl chains, thereby possessing significant deglutarylase, desuccinylase, and demalonylase activities.^16^, ^27^, ^28^, ^29^ SIRT6 is also a weak deacetylase, while it has preferential activity toward long‐chain fatty acyl moieties (e.g., myristoyl) and also possesses a mono‐ADP‐ribosyltransferase activity.^30^, ^31^ Lastly, SIRT7 has deacetylase and desuccinylase activities,^32^ and was recently shown to possess auto‐ADP‐ribosylation activity^33^ and a broad spectrum deacylase activity in vitro, with a preference for hexanoyl, octanoyl, decanoyl, and lauryl‐containing substrates.^34^, ^35^ The catalytic activity of SIRTs is modulated via multiple mechanisms, which include protein–protein interactions, PTMs, and binding of endogenous molecules. Moreover, their biological function is also regulated at transcriptional level and through the modulation of their degradation. These mechanisms have been recently reviewed by Wang and Lin.^36^

Given their central role in different cellular processes, SIRTs have attracted increasing research interest aimed at developing both activators and inhibitors. In this review, we summarize the biological roles of SIRTs and examine the most relevant SIRT activators and inhibitors. We provide a detailed analysis of their pharmacology and structure–activity relationships (SAR), along with an overview of the clinical trials in which SIRT modulators have been used and a critical perspective on the state‐of‐the‐art.

## 2 BIOLOGICAL FUNCTIONS OF SIRTUINS

### 2.1 SIRT1

SIRT1 is mostly a nuclear protein, with a small fraction present in the cytosol. It is the first sirtuin to be discovered given its similarity to the yeast protein silent information regulator 2 (Sir2).^37^ SIRT1 function has been linked to aging given its manifold roles in stress adaptation,^38^ cell cycle regulation,^39^ and DNA damage response.^40^ Given its expression in calorie‐restricted cells, SIRT1 has a central role in several metabolic processes such as gluconeogenesis, lipogenesis, and fatty acid β‐oxidation.^41^ Moreover, SIRT1 has been shown to inhibit inflammation through the lysine deacetylation of different factors, including nuclear factor kappa B (NF‐κB), activator protein 1 (AP‐1), and signal transducer and activator of transcription (STAT3).^42^ SIRT1 also deacetylates sterile alpha motif and HD domain‐containing protein 1 (SAMHD1), a deoxyribonucleoside triphosphohydrolase which facilitates DNA double‐strand break (DSB) repair independently from its catalytic activity. Specifically, SIRT1 deacetylates SAMHD1 at K354 and promotes its recruitment at DSBs, finally facilitating homologous recombination.^43^

SIRT1 is phosphorylated by several kinases which modulate its activity. For instance, casein kinase 2 (CK2) phosphorylates SIRT1 at four Ser residues (S154, S649, S651, and S683 in mice) and promotes its activity following exposure to ionizing radiations thereby facilitating SIRT1‐mediated DNA repair.^44^ Nonetheless, the same kinase was shown to phosphorylate (at S164) and inactivate SIRT1 in obese mice.^45^ SIRT1 phosphorylation also affects its role in metabolism. For instance, mTOR phosphorylates SIRT1 at S47, thereby impairing its catalytic activity.^46^ Conversely, phosphorylation in the catalytic pocket (at S434) by protein kinase A (PKA) activates SIRT1. Hence, SIRT1 activation is one of the downstream effects of adrenergic signaling, which is usually initiated in conditions of increased fatty acid consumption and energy production.^47^, ^48^ This is in line with reports indicating SIRT1 as one of the key lipolysis inducers, while it inhibits lipogenesis. Finally, SIRT1 activity is governed by REGγ, the 11S proteasome regulatory complex, through ubiquitin‐independent proteasomal degradation. Indeed, SIRT1 levels were higher in the liver of REGγ‐knockout mice, thereby promoting autophagy mediated lipid metabolism and decreasing hepatic steatosis. Moreover, the regulation of SIRT1 by REGγ is influenced by the energy condition of the cells.^49^

SIRT1 has roles in several neurodegenerative conditions such as Alzheimer's (AD), Huntington's (HD), and Parkinson's (PD) disease, where it seems to exert neuroprotective functions.^50^, ^51^ For instance, in AD, SIRT1‐mediated deacetylation of proteins involved in the astrocyte lysosomal pathway triggers β‐amyloid degradation in primary astrocytes, resulting in an increased number of lysosomes.^52^ Notably, β‐amyloid was indicated to downregulate SIRT1 in vitro, and its overexpression could rescue β‐amyloid‐induced senescence and mitochondrial dysfunction.^53^ In a mouse model of PD, overexpression of SIRT1 seemed to improve the prognosis, since it led to reduction of α‐synuclein aggregates and diminished reactive gliosis.^51^ In line with this, in an HD mouse model, SIRT1 overexpression increased neurotropic factor expression and survival rate, while its knockout worsened the pathological setting.^54^ Conversely, mutant huntingtin deacetylation by SIRT1 prevents its degradation^55^ and experiments in a *Drosophila melanogaster* HD model demonstrated that inhibition or knockout of Sir2, the SIRT1 homolog in *D. melanogaster*, are neuroprotective.^56^ Further research examining cellular and animal HD models revealed that inhibiting human SIRT1 pharmacologically may halt the neurodegenerative process and recover neuronal functioning.^57^

Furthermore, SIRT1 is implicated in cardiovascular diseases^58^ and its levels are lower in the heart tissues of rats and humans affected by heart failure.^59^, ^60^ Specifically, SIRT1 decreased levels were suggested to cause antioxidant factors downregulation and upregulation of proapoptotic proteins as a consequence of increased p53 acetylation and reduced translocation in the nucleus of forkhead box O 1 (FOXO1) protein.^59^ SIRT1 also exerts protective functions in atherosclerosis according to both in vitro and in vivo studies.^61^, ^62^ SIRT1 was shown to trigger DNA damage repair by deacetylating the repair protein Nijmegen Breakage Syndrome‐1 (NBS1) in vascular smooth muscle cells (VMSCs).^62^ SIRT1 also decelerates cardiomyopathies by protecting cardiomyocytes from oxidative stress and preventing apoptosis.^63^, ^64^ More recently, SIRT1 was shown to reduce inflammasome signaling and apoptosis by modulating the NF‐κB pathway and this activity is responsible for the neuroprotective effects of the mesenchymal stem cell therapy following ischemic stroke.^65^ Recently, SIRT1 was indicated to deacetylate histone H2AX at Lys5, which in turn triggers its phosphorylation at Ser139. This sequence of events activates the DNA damage repair machinery and counteracts the doxorubicin‐induced cardiotoxicity in mouse models.^66^

SIRT1 has been also shown to have a key role in autoimmune and chronic diseases. In contrast with its role in neurodegeneration, SIRT1 was suggested to contribute to the activity of reactive astrocytes. Indeed, SIRT1 astrocyte‐specific knockout in mouse models was shown to block the progression of autoimmune encephalomyelitis.^67^ Furthermore, SIRT1 activity promotes the self‐renewal and differentiation of type 2 alveolar epithelial cells in lung tissues of old mice and patients affected by idiopathic pulmonary fibrosis (IPF) and its expression is dependent on the levels of the zinc transporter SLC39A8 (ZIP8).^68^

In cancer, SIRT1 possesses a dichotomous role by regulating the expression and function of a wide array of proteins at different phases of cancer development.^69^ Beyond influencing the expression of the onco‐suppressor p53,^70^ SIRT1 directly inactivates this protein via deacetylation.^71^ SIRT1 also stimulates the activity of the DNA repair protein Ku70 through deacetylation, therefore inhibiting Bcl2‐associated X protein (Bax)‐mediated apoptosis.^72^ Moreover, SIRT1‐mediated deacetylation of FOXO family proteins suppresses FOXO‐dependent transcription and apoptosis pathways.^72^ Conversely, SIRT1 may also protect cells against oncogenic mutations by promoting apoptosis. For instance, NF‐κB deacetylation sensitizes cancer cells to apoptosis triggered by tumor necrosis factor α (TNF‐α).^7^, ^73^ Notably, SIRT1 expression is affected by p53 and FOXO3a. Indeed, both proteins promote SIRT1 transcription during starvation conditions, thereby creating a negative feedback mechanism.^74^ SIRT1 is overexpressed in acute myeloid leukemia (AML), chronic myeloid leukemia (CML), diffuse large B‐cell lymphoma (DLBCL), melanoma, lung, and gastric cancer. Conversely, it is downregulated in glioma and bladder cancer.^75^ Furthermore, SIRT1 exhibits both oncogenic and oncosuppressor functions in colorectal carcinoma (CRC), prostate, and breast cancer.^75^, ^76^ SIRT1 inhibits the activity of the transcription factor NF‐κB whose activity promotes cell survival and immune response during tumorigenesis. Conversely, SIRT1 acts as a tumor promoter by inhibiting the activity of oncosuppressors through their deacetylation. These include p53, FOXO proteins, E2F transcription factor 1 (E2F1), and the retinoblastoma protein (pRb).^76^ Furthermore, SIRT1 promotes DNA damage repair by activating the relevant enzymes through deacetylation, thus impairing the onset of cancer on the one hand while simultaneously promoting cancer cell proliferation at later stages on the other.

### 2.2 SIRT2

SIRT2 is primarily a cytoplasmic protein, with α‐tubulin being one of its main targets,^77^ and is mainly localized in the brain, where it seems to be involved in several disorders.^78^ In AD, SIRT2 has been shown to deacetylate Reticulon 4B (RTN4B), triggering its ubiquitination and degradation.^79^ RTN4B expression is oppositely correlated with the production of β‐secretase 1 (BACE1), an enzyme that leads to the generation of Aβ peptides. In line with this, suppression of SIRT2 activity was reported to reduce BACE1 expression, finally lowering Aβ levels and ameliorating cognitive functions in mouse models of AD.^79^ In PD, SIRT2 inhibition is protective against α‐synuclein mediated neuronal toxicity.^80^ Similarly, in HD mouse models, SIRT2 inhibition increases lifespan and is protective for neurons through a reduction of the polyglutamine accumulation rate at the *N*‐terminus of huntingtin.^81^ SIRT2 is implicated in the control of cell cycle and apoptosis via deacetylation of H4K16 and p53,^82^ as well as p65, which enables the regulation of NF‐κB controlled genes.^83^

SIRT2 has also a central role in metabolism regulation. During adipogenesis, SIRT2 expression is decreased while its levels in the adipose tissue augment following caloric restriction. In line with this, in diet‐induced obese mice, the hypoxia‐inducible factor‐1α (HIF‐1α), a key protein for the activation of glycolysis‐associated genes, inhibits SIRT2 transcription, impairing fatty acid β‐oxidation and energy consumption.^84^ Notably, SIRT2 was shown to deacetylate HIF1α, thereby facilitating its degradation, in a sort of negative feedback loop.^85^ Intriguingly, SIRT2 and HIF1α have also opposite activities in the regulation of vascularization. Indeed, while HIF‐1α promotes vascularization, SIRT2 was shown to be recruited by seryl‐tRNA synthetase to chromatin to reduce vascular endothelial growth factor A (VEGF‐A) transcription.^86^

In cardiovascular settings, SIRT2 was shown to exert a protective function against cardiac hypertrophy. Mechanistically, SIRT2 deacetylates the nuclear factor of activated T‐cells, cytoplasmic 2 (NFATc2) transcription factor, thereby impairing its transcription activity. In line with this, NFATc2 inhibition could rescue the cardiac dysfunction in SIRT2‐knockout mice.^87^ Moreover, SIRT2 was indicated to deacetylate the liver kinase B1 (LKB1), which in turn phosphorylates and activates AMPK, finally modulating gene expression leading to cardioprotection.^88^ In atherosclerosis, SIRT2 activity is correlated with reduced plaque formation in LDL receptor knockout mice through inhibition of macrophage polarization to the M1 phenotype.^89^

In the context of autoimmune diseases, SIRT2 was shown to promote the development of systemic lupus erythematosus (SLE). Hisada et al. showed that SIRT2 deacetylates multiple targets, including p70S6K and *c*‐Jun, finally promoting interleukin (IL)−17A expression, Th17‐cell differentiation, and decreasing the production of IL‐2, which are key factors contributing to the onset of SLE.^90^

SIRT2 also plays a dual role in cancer, depending on the specific context.^91^ Its expression is upregulated in certain types of cancers (e.g., neuroblastoma, renal cell carcinoma, uveal melanoma, AML, osteosarcoma) while it is downregulated in others (non‐small cell lung cancer [NSCLC], CRC, breast and gastric cancer).^92^ For instance, in osteosarcoma, SIRT2 deacetylates the epithelial‐mesenchymal transition (EMT)‐associated factor Snail, thus inhibiting its degradation and promoting cancer cell proliferation, invasion, and metastasis.^93^ Conversely, in CRC, SIRT2 deacetylates isocitrate dehydrogenase 1 (IDH1), therefore promoting its activity and the consequent production of α‐ketoglutarate and leads to decreased levels of HIF‐1α and the proto‐oncogene SRC. This finally leads to impaired invasion and metastasis of CRC cells.^94^ In some cancer types, SIRT2 plays both a tumor promoting and tumor suppressing role. For instance, in breast cancer cells, SIRT2 was shown to inhibit peroxiredoxin‐1 via deacetylation, which decreased its antioxidant peroxidase activity. This in turn increases the sensitivity to reactive oxygen species (ROS)‐induced DNA damage, finally leading to cancer cell death.^95^ Conversely, in breast cancer stem cells (CSCs), SIRT2 was found to promote the activity of aldehyde dehydrogenase 1A1 (ALDH1A1) via deacetylation, which in turn facilitates breast CSCs activation and self‐renewal.^96^

### 2.3 SIRT3

SIRT3 is predominantly a mitochondrial protein, mainly located in kidneys, heart, and liver.^97^ Nevertheless, a long‐chain SIRT3 isoform still containing the *N*‐terminal mitochondrial targeting has been reported in the nucleus,^98^, ^99^ although different reports describe this isoform as catalytically inactive,^100^ and some others suggest that SIRT3 is exclusively mitochondrial.^101^, ^102^ Hence, the relevance of long‐chain SIRT3 remains unclear and additional experiments are required to resolve this discrepancy.

Given its mitochondrial localization, it comes with no surprise that SIRT3 plays key roles in metabolism regulation. For instance, SIRT3 was shown to deacetylate acetyl‐CoA synthetase 2 (AceCS2), a mitochondrial enzyme responsible for the conversion of acetate to acetyl‐CoA. SIRT3‐mediated deacetylation at K642 leads to increased AceCS2 activity, as confirmed by knockdown studies.^103^, ^104^ Similarly, SIRT3 was indicated to regulate fatty‐acid oxidation by deacetylating long‐chain acyl‐CoA dehydrogenase (LCAD)^105^ and was also shown to target medium‐chain ACAD (MCAD) and acyl‐CoA dehydrogenase 9 (ACAD‐9).^106^ Finally, SIRT3 promotes ketone body synthesis via deacetylation and activation of hydroxyl methylglutaryl‐CoA synthase 2 (HMGCS2).^107^ SIRT3 also enhances the activity of the pyruvate dehydrogenase subunit E1α (PDHA1) through deacetylation, facilitating acetyl‐CoA production from pyruvate.^108^ Moreover, SIRT3 was shown to deacetylate and activate many complexes of the electron transport chain (ETC) such as NADH dehydrogenase [ubiquinone] 1 α subcomplex subunit 9 (NDUFA9), subunits α and β of the ATP synthase, and succinate dehydrogenase.^109^

SIRT3 is also a sensor of mitochondrial health. Indeed, SIRT3 undergoes a pH‐dependent interaction with ATP synthase via the ATP5O subunit, presenting a key His residue (H135) whose protonation is essential for SIRT3 binding. Under physiological conditions, SIRT3 binds to ATP synthase, but following pH reduction of the mitochondrial matrix and depolarization, SIRT3 disassociates from ATP synthase and diffuses in the matrix where it deacetylates many proteins, thus promoting restoration of normal membrane potential.^106^

Furthermore, SIRT3 mediates M2 macrophage activation through deacetylation, and consequent activation of the mitochondrial enzyme glutamate dehydrogenase 1 (GLUD1). The subsequent increase of α‐ketoglutarate levels causes a metabolic switch toward oxidative phosphorylation and reduction of histone H3K27 trimethylation, finally upregulating genes related to macrophage polarization.^110^

To date, SIRT3 has been mainly described as a neuroprotective factor. In a mouse model of HD, SIRT3 upregulation played a therapeutic role since it diminished striatal neuron degeneration, leading to increased neuronal survival and ameliorated motor functions.^111^ Furthermore, SIRT3 counteracts oxidative stress and synuclein in dopaminergic neurons, thereby acting as a protective factor against aging.^112^ During caloric restriction, isocitrate dehydrogenase 2 (IDH2) is activated by SIRT3 via deacetylation, leading to increased levels of glutathione in its reduced form and nicotinamide adenine dinucleotide phosphate (NADPH) in the mitochondria, which protects from oxidative pressure.^113^ Manganese superoxide dismutase (SOD2) is also activated by SIRT3‐mediated deacetylation, exerting a protective role against oxidative stress in the microglia.^114^

The activity of SIRT3 has also crucial implications on cardiac function and cardiovascular disorders. Indeed, SIRT3 seems to mitigate diabetic cardiomyopathy by deacetylating the transcription factor p53, consequently decreasing the expression of the fructose‐2,6‐bisphosphatase TP53‐induced glycolysis and apoptosis regulator (TIGAR).^115^ Moreover, SIRT3 was shown to indirectly enhance the expression of 6‐phosphofructo‐2‐kinase/fructose‐2,6‐bisphosphatase 3 (PFKFB3).^116^ Altogether, these two activities increase the levels of fructose‐2,6‐bisphosphate, an activator of phosphofructokinase‐1, which is crucial in increasing glycolysis under hyperglycemic conditions.^115^ Another study showed that SIRT3 knockout in endothelial cells reduces the glycolytic process mediated by PFKFB3 while augmenting the consumption rate of oxygen and the formation of ROS, thus contributing to cardiac hypoxia and apoptosis. In vivo studies showed that SIRT3‐knockout mice develop microvascular and diastolic dysfunction. Hence, alteration of endothelial SIRT3 function may contribute to microvascular rarefaction and heart failure.^117^ SIRT3 was also shown to be downregulated in mouse models of hypertensive heart failure which were characterized by hyperacetylation of the mitochondrial proteins.^118^ In line with this, SIRT3 levels were reported to be reduced in failing myocardium and SIRT3 knockout mice exhibited elevated levels of acetylation of PDH and ATP synthase, which resulted in decreased enzymatic activity.^119^

SIRT3, like other sirtuins, has dual implications in tumorigenesis.^120^ For instance, SIRT3 is overexpressed in head and neck squamous carcinomas, where its protective actions against ROS determine apoptosis prevention, which facilitates cancer. In DLBCL, SIRT3 activates glutamate dehydrogenase (GDH) thereby enhancing tricarboxylic acid (TCA) cycle metabolism and promoting cancer onset and development.^121^ Moreover, the above‐mentioned deacetylation of IDH2 by SIRT3 promotes tumor growth in multiple myeloma.^122^ Conversely, SIRT3 is downregulated in hepatocellular, breast, and prostate cancer.^123^, ^124^ SIRT3 exerts its oncosuppressor role via deacetylation and consequent inhibition of HIF‐1α which in turn leads to activation of prolyl hydroxylases, thus facilitating HIF‐1α hydroxylation and consequent proteasomal degradation.^116^ This function contributes to the disruption of the Warburg effect, an alteration of glucose metabolism typical of cancer cells where ATP is acquired mostly by glycolysis even when oxygen is present, to quickly produce energy and support cancer cell growth.^125^

### 2.4 SIRT4

SIRT4 is mainly located in mitochondria and possesses mono ADP‐ribosyltransferase,^126^ lipoamidase,^127^ deacetylase,^21^, ^128^ decarbamylase,^24^ and broad spectrum deacylase activities. Specifically, SIRT4 catalyzes the in vitro and in vivo removal of HMG and structurally related modifications, such as glutaryl, 3‐methylglutaryl, and 3‐methylglutaconyl, with a preference for HMG.^25^, ^26^ SIRT4‐mediated de‐HMGylation is implicated in insulin secretion via modulation of leucine metabolism.^26^ SIRT4 dysregulation has been linked to metabolic and ageing‐related disorders, including type 2 diabetes, nonalcoholic fatty liver disease, neurodegeneration, and cardiac hypertrophy.^129^, ^130^ All these outcomes are directly connected to the wide range of cellular functions regulated by the enzymatic activity of SIRT4, which include carbon entry in the TCA cycle, amino‐ and fatty acid metabolism, insulin secretion, ROS generation, ATP homeostasis, and apoptosis regulation.^129^, ^130^ Indeed, SIRT4 acts as a checkpoint between glycolysis and the TCA cycle by catalyzing the delipoylation of pyruvate dehydrogenase (PDH).^127^ This is a multiprotein complex that performs the oxidative decarboxylation of pyruvate to yield acetyl‐CoA and therefore links glycolysis to the TCA cycle. Notably, SIRT4‐mediated delipoylation decreases PDH activity in cells and in vivo, indicating the key role of SIRT4 as a master regulator of metabolism.^127^ Moreover, SIRT4 was shown to inhibit GDH via mono ADP‐ribosylation and its activity leads to lower insulin secretion in pancreatic β cells.^126^ A recent study indicated that SIRT4 possesses decarbamylase activity and targets ornithine transcarbamylase (OTC), a key enzyme of the urea cycle which is inactivated by decarbamylation. Interestingly, SIRT4 is upregulated in amino acid insufficiency conditions and its knockout led to high levels of urea cycle intermediates in cells and prevented hepatic encephalopathy in mice. Overall, these results suggest a key role of SIRT4 in ammonia metabolism regulation.^24^

Like SIRT1, SIRT4 was shown to reduce doxorubicin‐induced cardiotoxicity. In this case, SIRT4 expression increases the levels of Bcl2 and activates the Akt/mTOR pathways, thus inhibiting apoptosis and autophagy.^131^ Moreover, SIRT4 has a protective role against myocardial ischemia/reperfusion injury which is connected with decreased apoptosis and retained mitochondrial function of myocardial cells.^132^

SIRT4 mostly has a tumor suppressor role via modulation of DNA damage response in both healthy and cancerous cells^133^ and by impairing glutamine catabolism.^134^ In line with this, SIRT4 is downregulated in various cancer types, including breast, thyroid, lung, and prostate cancer.^135^ In pancreatic ductal adenocarcinoma (PDAC), SIRT4 activity was associated with higher p53 phosphorylation as a consequence of GDH inhibition, which finally led to autophagy promotion and tumor growth inhibition both in vitro and in vivo.^136^ Nonetheless, SIRT4 may also play a tumor promoting role since its activity in cancer cells is protective against endoplasmic reticulum (ER) stress and DNA damage, thus facilitating cancer cell survival and proliferation, as shown in the case of hepatocellular carcinoma (HCC) HepG2 cell line.^137^

### 2.5 SIRT5

Analogously to SIRT3 and 4, SIRT5 is mostly a mitochondrial protein.^138^ It is mainly distributed in heart, kidney, muscles, liver, brain, and testis.^139^, ^140^ SIRT5 has a weak deacetylase activity, while it displays the highest catalytic efficiency for protein lysine deglutarylation, followed by desuccinylation and demalonylation.^16^, ^27^, ^28^, ^29^, ^141^

Most of the SIRT5 substrates are involved in oxidative stress response and energetic metabolism processes, including glycolysis, pentose phosphate pathway, fatty acid oxidation, ketone body formation, ammonia detoxification, and glutamine metabolism.^140^, ^142^, ^143^ For instance, SIRT5 was indicated to desuccinylate and inhibit IDH2, which converts isocitrate to α‐ketoglutarate.^144^ Differently, SIRT5 activates through demalonylation, glyceraldehyde 3‐phosphate dehydrogenase (GAPDH), thus promoting glycolysis.^145^ Another glycolytic enzyme targeted by SIRT5 is pyruvate kinase M2 (PKM2), which catalyzes the conversion of phosphoenolpyruvate in pyruvate when in its tetrameric form, while it acts as a nuclear protein kinase as a dimer. The effects of SIRT5 activity on PKM2 have been investigated in multiple studies. A report by Wang and colleagues indicates that in activated macrophages SIRT5 activates PKM2 through desuccinylation K311, thus sustaining glycolysis.^146^ Another study, conducted in lung cancer cells, indicated that SIRT5 inhibits PKM2 through desuccinylation of K498 during oxidative stress, thus impairing glycolysis.^147^ Finally, Qi et al. showed that SIRT5‐mediated desuccinylation blocks PKM2 translocation from nucleus to mitochondria and the consequent interaction with dependent anion channel 3 (VDAC3), which is then degraded and leads to higher mitochondrial permeability and apoptosis in CRC cells.^148^ SIRT5 also desuccinylates the enzymatic respiratory complex II succinate dehydrogenase (SDH), which oxides succinate to fumarate and reduces ubiquinone to ubiquinol and is implicated in both TCA cycle and ETC. SIRT5‐mediated desuccinylation inhibits SDH, thus impairing cellular respiration.^149^ SIRT5 is also implicated in thermogenesis, as showed by a recent study indicating that its knockdown in mice reduces the activity of uncoupling protein 1 (UCP1, also called thermogenin). Shuai et al. also indicated that SIRT5 is pivotal for brown adipogenetic gene expression and for the differentiation of white adipose tissue into brown adipose tissue (BAT).^150^ Given the implication of BAT in glucose metabolism, SIRT5 has been suggested as a promising target for metabolic disorders such as type 2 diabetes and obesity.^151^

Considering its function in controlling metabolism and ROS levels, it is not surprising that SIRT5 is a protective factor in neurodegenerative diseases.^152^ In line with this, SIRT5 was shown to reduce the detrimental effects of the convulsant MPTP and reduce ROS levels in nigrostriatal dopaminergic neurons.^153^ Studies performed in AD mice demonstrated that SIRT5 expression decreases neuronal inflammation and damage.^154^ Furthermore, in vivo studies showed that SIRT5 is a protective factor against epileptic diseases.^155^

SIRT5 plays a pivotal role in cardiac physiology, especially under stress conditions.^28^ A recent study compared wild type mice, SIRT5‐overexpressing mice, and SIRT5 knockout mice, all of them characterized by cardiac hypertrophy and heart failure. The authors demonstrated that SIRT5 activity is positively correlated with a reduction in fibrosis and an enhancement in cardiac function, while an increased susceptibility to cardiac ischemia–reperfusion injury is associated with SIRT5 downregulation. Specifically, SIRT5 was proposed to modulate the inflammatory response and fibroblast activation which affect cardiac function through desuccinylation and activation of PKM2.^156^

Recent studies also suggest an important role of SIRT5 in inflammation. Indeed, SIRT5 overexpression mitigates mitochondrial dysfunction in renal tubular epithelial cells during septic acute kidney injury. Specifically, SIRT5 expression is positively correlated with high phospho‐AMPK levels and ATP production, along with downregulation of proapoptotic factors and decreased ROS production.^157^ On the other hand, SIRT5 seems to induce neuroinflammation and represents a risk factor for ischemic stroke. Xia and colleagues showed that SIRT5 desuccinylates Annexin‐A1 inhibiting its membrane recruitment and secretion. This in turn causes overexpression of inflammatory cytokines, microglial activation and, finally, neuronal damage following ischemic stroke.^158^

SIRT5 was recently indicated to play an important role in the development COVID‐19 caused by SARS‐CoV‐2 by interacting with the SARS‐CoV‐2 nonstructural viral protein 14 (NSP14). Nevertheless, while one study concludes that SIRT5 activity inhibits viral replication,^159^ the other suggests that SIRT5 instead supports it.^160^ Hence, further in‐depth studies will be necessary to shed light on the connection between SIRT5 and COVID‐19.

In cancer, SIRT5 acts as either a tumor promoter or suppressor in a context‐dependent fashion.^161^ Recently, SIRT5 has been reported to act as a tumor promoter in several cancer types, including ovarian and breast cancer,^162^, ^163^ CRC,^164^, ^165^ AML,^166^ and melanoma.^167^ SIRT5 tumor promoting activity is mediated by its substrates involved in metabolic regulation, including serine hydroxy methyltransferase 2 (SHMT2). Specifically, desuccinylation of K280 activates SHMT2, which in turn supports tumorigenesis in osteosarcoma U2OS and CRC HCT116 cell lines.^168^ In line with this, SIRT5 knockout or expression of succinylation mimicking mutant of SHMT2 (K280E) led to the suppression of tumor development in vitro and in vivo.^168^ Moreover, SIRT5 was shown to desuccinylate p53 at K120, thus impairing its transcriptional activity and the apoptosis induction after DNA damage, finally sustaining cancer onset and development.^169^ A recent study showed that SIRT5 demalonylates and activates transketolase, thus sustaining the production of ribose‐5‐phosphate and the consequent biosynthesis of nucleotides, thereby safeguarding CRC cells against DNA damage.^165^ SIRT5 plays a tumor suppressor role in head and neck squamous cell carcinoma,^170^ glioma,^171^ endometrial carcinoma,^172^ PDAC,^173^ and gastric cancer.^174^ In PDAC, SIRT5 was reported to deacetylate glutamic‐oxaloacetic transaminase 1 (GOT1), which converts α‐ketoglutarate and aspartate into glutamate and oxaloacetate, which in turn increased the levels of NADPH and reduced glutathione (GSH) which contribute to the maintenance of cancer cells redox homeostasis. SIRT5‐mediated GOT1 inhibition reduces the levels of NADPH and GSH so disrupting this homeostatic mechanism. In line with this, SIRT5 knockdown determined ROS level reduction and the hyperproliferation of cancer cells. Conversely, pharmacological activation (see compound **5g**, “SIRT Activators” section) reduced GOT1 activity and decreased PDAC cell viability.^173^ Notably, in HCC,^175^, ^176^, ^177^ breast,^142^, ^178^, ^179^ prostate,^180^, ^181^ and lung cancer,^153^, ^182^, ^183^, ^184^ SIRT5 showed contrasting roles, either supporting or repressing tumor development, suggesting that its function is related to both tissue type and disease stage. For instance, in HCC, SIRT5 is highly expressed in primary tumors and its levels are even higher in the metastatic ones, with its activity being mainly associated with cell proliferation and invasion.^176^ Conversely, its tumor suppressor activity was found to be important in healthy or primary HCC cells. SIRT5 desuccinylates and inhibits acyl‐CoA oxidase 1 (ACOX1), a peroxisomal enzyme whose overactivity causes ROS‐mediated DNA damage as well as alteration of fatty acid β‐oxidation and redox homeostasis, ultimately leading to the onset of HCC.^177^ In the case of prostate cancer, one study indicated that SIRT5 promotes cancer cell proliferation and migration by targeting acetyl‐CoA acetyltransferase 1 (ACAT1). This ultimately results in the activation of the MAPK pathway and the upregulation of cyclin D1 and matrix metallopeptidase 9 (MMP9).^180^ In contrast, SIRT5 knockout increases the proliferation, migration, and invasion of prostate cancer cells, according to a recent report, which also proposed that the tumor suppressor activity of SIRT5 is facilitated via the desuccinylation of lactate dehydrogenase A (LDHA).^181^

### 2.6 SIRT6

SIRT6 is a deacetylase, deacylase, and mono ADP‐ribosyltransferase mainly located in the nucleus, whose activity is linked to improved health and longevity.^185^, ^186^ This is most likely due to SIRT6 implication in various pathways such as metabolism, aging, DNA damage response, differentiation, immunity, inflammation, and circadian rhythm control.^186^, ^187^ Mechanistically, SIRT6 deacetylates H3K56, which in turn increases chromatin accessibility and facilitates DNA repair.^188^ Similarly, H3K9 and H3K56 deacetylation at telomeric regions mediates telomeric preservation.^189^, ^190^ Furthermore, H3K9 and H3K56 deacetylation by SIRT6 causes the downregulation of *c*‐Myc target genes, ribosomal proteins, genes involved in early development, and NF‐κB‐dependent proteins, which are involved in inflammation and lipid metabolism.^191^, ^192^, ^193^ Moreover, SIRT6‐mediated mono‐ADP‐ribosylation of the corepressor KAP1 contributes to the downregulation of long interspersed element‐1 (LINE‐1) retrotransposable elements,^194^ a group of retrotransposons linked to mutagenesis and genomic instability. Specifically, SIRT6 facilitates their heterochromatin packaging and suppresses their transposition. Finally, SIRT6‐mediated demyristoylation of TNF‐α triggers its secretion, thereby promoting inflammation.^31^ Nevertheless, its role in inflammation is context‐dependent, since it was shown that, through deacetylation of H3K9 and H3K56 at gene promoters, SIRT6 reduces the expression of HIF‐1α, *c*‐Jun, and NF‐κB, while increasing Nrf2 expression, finally downregulating adhesion factors, including VCAM1 and ICAM1.^195^, ^196^

SIRT6 has a pivotal role in the regulation of aging‐related diseases. Recently, Ji et al. demonstrated that SIRT6 decelerates osteoarthritis progression by inhibiting the IL‐15/JAK3/STAT5 axis.^197^ In particular, SIRT6 was found to deacetylate the transcription factor STAT5 on K163. This stops the IL‐15/JAK3‐mediated STAT5 phosphorylation as well as its translocation inside the nucleus. Consequently, STAT5‐mediated transcription of chondrocyte senescence genes is suppressed, thus decelerating osteoarthritis progression. In line with this, activation of SIRT6 with MDL‐800^198^ (compound **7a** in the “SIRT Activators” section) was shown to prevent chondrocyte senescence and osteoarthritis development.^197^ On the other hand, SIRT6 expression is linked to glucocorticoid‐induced myotube size reduction in mouse models of skeletal muscle atrophy. Mechanistically, SIRT6 deacetylates H3K9 at the promoter of IGF2, thus inhibiting the transcription of *c*‐Jun‐regulated genes, finally repressing the IGF/Akt pathway.^199^ In line with this, SIRT6‐knockout mice exhibited increased Akt signaling and IGF2 expression, with downregulation of FOXO family transcription factors, which are associated with the expression of atrophy‐related genes. The authors found that pharmacological inhibition of SIRT6 with the quinazolidinedione derivative **42a** ^200^ (see “SIRT Inhibitors” section) could repress muscle atrophy development in mice.^199^ Nevertheless, the compound used is a weak and not selective SIRT6 inhibitor since it also targets SIRT2 with an IC_50_ value in the same range of potency (roughly 100 μM).

In the context of cardiovascular diseases, SIRT6 was shown to act as a protective factor against cardiac hypertrophy in both in vitro and in vivo experiments,^201^ and it does so through multiple mechanisms. First, it facilitates the retention of the FOXO3 transcription factor within the nucleus, potentially through the inhibition of Akt signaling, the pathway underlying autophagy activation.^202^ SIRT6 additionally prevents hypertrophy of cardiomyocytes by reducing p300 levels and the acetylation of the p65 subunit of NF‐κB.^203^ Finally, SIRT6 inhibits the development of cardiac hypertrophy and heart failure by deacetylating H3K9 and repressing the transcriptional activity of *c*‐Jun and IGF/Akt signaling.^204^ In line with this, SIRT6 was downregulated in both animal models of heart failure and patient hearts affected by chronic heart failure.^205^ SIRT6 also acts as a protective factor against thoracic aortic aneurysms. Mechanistically, SIRT6 inhibits vascular inflammation by deacetylating H3K9 and H3K56 at the *Il1b* promoter, thereby suppressing IL‐1β expression.^206^

As mentioned above, SIRT6 also has central function in the regulation of the inflammatory response. Specifically, SIRT6 has a protective role against atherosclerosis by preventing DNA damage, inhibiting apoptosis and inflammatory response, finally inhibiting the senescence of VSMCs.^207^ In line with this, SIRT6 was shown to decrease the expression of atherosclerosis‐inducing factors such as TNFSF4 (tumor necrosis factor superfamily member 4), by deacetylating H3K9 at their promoter.^195^ Moreover, SIRT6 was recently found to deacetylate Caveolin‐1, thereby decreasing the transcytosis through endothelial cells of low‐density lipoprotein and delaying the onset of atherosclerosis in diabetic mice.^208^

In the context of nervous system physiology and pathology, SIRT6 has been indicated as a pivotal factor for mitochondrial activity in the central nervous system (CNS) and its knockout in mouse models led to global mitochondrial dysfunction and an alteration of metabolite levels. Moreover, by interacting with the transcription factor YY1, SIRT6 promotes SIRT3 and SIRT4 expression as well as cellular respiration in the brain.^209^ Moreover, SIRT6 can reduce oxidative stress and the expression of pro‐inflammatory factors in the brain and suppress apoptosis, thereby attenuating spinal cord injury.^210^

Analogously to SIRT2 and 3, SIRT6 antagonizes HIF‐1α activity, though with a different mechanism. In this case, SIRT6 inhibits HIF‐1α transcription and also reduces the expression of glycolytic genes via H3K9 deacetylation.^211^ Given its pleiotropic activity at different levels of cellular homeostasis, it is not surprising that dysregulation of SIRT6 is also associated with cancer. Similar to other SIRTs, SIRT6 has a double‐faced role in cancer, depending on the specific context.^212^ Indeed, its protective role toward DNA damage has been shown to defend cancer cells from genotoxic drugs and support their proliferation, as in the case of prostate cancer,^213^ breast cancer,^214^ AML,^215^ ovarian cancer,^216^ and multiple myeloma.^217^ Moreover, it was shown to promote cancer cell‐induced inflammation, angiogenesis, migration and, finally, metastasis in PDAC.^218^ For instance, in AML, SIRT6 binds DNA damage sites where it deacetylates and activates DNA‐dependent protein kinases and the endonuclease CtIP, thereby activating DNA repair mechanisms which promote cell proliferation.^215^ Conversely, SIRT6‐mediated suppression of glycolytic genes and HIF‐1α expression opposes the Warburg effect and has a tumor suppressive role in CRC^219^ and bladder cancer.^220^ SIRT6 also suppresses cancer proliferation in NSCLC, PDAC, glioma, endometrial carcinoma, and melanoma via inhibition of multiple pathways.^212^ For instance, in NSCLC, SIRT6 decreases the expression of Twist1, a transcription factor which promotes EMT and metastasis. In PDAC, SIRT6 knockdown led to increased acetylation of H3K9 and H3K56 at the promoters of oncogenes Lin28b and *c*‐Myc, which resulted in cancer cell proliferation and metastasis.^221^

### 2.7 SIRT7

SIRT7 is a nucleolar sirtuin^32^ possessing lysine deacetylation and desuccinylation activities and is involved in the regulation of rDNA transcription, rRNA expression, genome stability, stress response, and cell proliferation.^32^, ^222^, ^223^, ^224^ Recently, SIRT7 was shown to have a broad spectrum deacylase activity in vitro, targeting hexanoyl, octanoyl, decanoyl, and lauryl‐containing substrates.^34^, ^35^ At the histone protein level, SIRT7 targets acetylated H3K18 and succinylated H3K122.^32^, ^224^ Notably, SIRT7‐mediated H3K122 desuccinylation promotes chromatin condensation and double‐strand break repair, thereby being crucial for DNA‐damage response and cell survival.^32^ Similarly, SIRT7 was found to deacetylate the ataxia‐telangiectasia mutated (ATM) protein, promoting its dephosphorylation by the phosphatase WIP1. This process is crucial for DNA stability and the knockdown of SIRT7 leads to aberrant ATM activity and altered DNA repair.^225^

SIRT7 also has a key role in the regulation of metabolism. Indeed, it inactivates, through deacetylation at K323, phosphoglycerate kinase 1 (PGK1), thereby acting as a regulator of glycolysis.^226^ Differently, under glucose starvation conditions, SIRT7 was indicated to increase glucose‐6‐phosphatase catalytic subunit (G6PC) expression, thereby modulating gluconeogenesis.^227^ Notably, SIRT7 has been recently shown to possess auto‐ADP‐ribosylating activity. Under glucose starvation, mono ADP‐ribosyl‐SIRT7 is recognized by the ADP‐ribose binding protein mH2A1. Upon interaction with mH2A1, SIRT7 is translocated to specific intergenic regions, thus regulating the transcription of genes implicated in cAMP signaling and autophagy and involved in the coordination of the response to calorie restriction.^33^

In the context of lipid metabolism, SIRT7‐knockout mice developed chronic hepatic steatosis as they accumulated greater quantities of triglycerides than wild‐type mice and this phenotype was reverted by SIRT7 restoration in the liver. Here, SIRT7 is recruited by *c*‐Myc and represses the transcription of ribosomal proteins through H3K18 deacetylation, thereby alleviating ER stress.^228^ SIRT7 is also involved in thermogenesis by modulating energy expenditure in BAT, according to a study by Yoshizawa et al. performed in mouse models.^229^ Mechanistically, SIRT7 deacetylates insulin‐like growth factor 2 mRNA‐binding protein 2 (IGF2BP2), which in turn impairs the translation of UCP1, thus decreasing energy consumption.^229^

SIRT7 was recently identified as implicated in PD. In a rat model that possesses the Parkinsonian phenotype, SIRT7 levels underwent an age‐dependent decrease in different regions of the CNS, including the cerebral cortex, cerebellum, basal ganglia, and brain stem.^230^ Moreover, experiments using a cell‐based PD model indicated that chemical oxidative stress following treatment with 1‐methyl‐4‐phenylpyridinium (MPP^+^) or hydrogen peroxide reduced SIRT7 levels.^231^ More recently, Lee and colleagues showed that the F‐box‐only protein 7 (FBXO7) acts as an adaptor protein in the SKP1–Cullin–1–F‐box (SCF) E3 ligase complex that promotes SIRT7 ubiquitination and consequent proteasomal degradation. Notably, treatment with hydrogen peroxide decreased SIRT7 levels through the FBXO7‐mediated pathway and abolished the cytoprotective effects of SIRT7.^232^

In the context of cardiovascular diseases, SIRT7 was recently shown to impair artery calcification by inhibiting, through deacetylation, RUNX2, a transcription factor that promotes osteogenesis. This activity is impaired by the microRNA miR‐125b‐5p which is upregulated under hyperglycemic conditions and impairs SIRT7 translation, thereby leading to coronary calcification in diabetic patients.^233^ SIRT7 was recently indicated to reduce ferroptosis and fibrosis in renal epithelial cells and impair EMT and lipid peroxidation, thereby mitigating renal damage during hypertension. Specifically, in a mouse model of hypertension, SIRT7 overexpression was associated with the activation of the KLF15/NRF2 axis.^234^

SIRT7 also has a role in viral infection. Specifically, SIRT7 was found to desuccinylate H3K122 associated with the covalently closed circular DNA (cccDNA) of hepatitis B virus, thus leading to transcriptional silencing.^235^

SIRT7‐catalyzed H3K18 deacetylation is linked to oncogenic transformation and cancer cell proliferation.^224^ SIRT7 was found overexpressed in HCC,^236^ breast,^237^ and thyroid^238^ tumors, and supports metastasis formation in gastric and prostate cancer.^239^ Specifically, SIRT7 promotes thyroid oncogenesis by inducing phosphorylation of Akt (also known as Protein Kinase B) and ribosomal protein S6 kinase beta‐1 (p70S6K1), whose activation has been shown to promote thyroid tumorigenesis. Mechanistically, SIRT7 suppresses the transcription of deleted in breast cancer‐1 (DBC1), an endogenous inhibitor of SIRT1, through deacetylation of H3K18Ac at its promoter. This leads to increased SIRT1‐mediated deacetylation of Akt and p70S6K1, which in turn triggers their phosphorylation and subsequent activation.^238^ Similar to other sirtuins, SIRT7 has a double‐faced role in cancer. For instance, in CRC, SIRT7 activity impairs cancer cell growth and invasion. Mechanistically, SIRT7 deacetylates the WD repeat domain 77 (WDR77) thereby reducing its interaction with protein arginine methyltransferase 5 (PRMT5) finally leading to diminished methylation of H4R3, which supports cell proliferation and migration.^240^ In the context of oral squamous cell carcinoma (OSCC), SIRT7 was shown to impair EMT in vitro by deacetylating the EMT mediator SMAD4 and its overexpression abolished OSCC lung metastasis in mouse models.^241^ SIRT7 was also found to be downregulated in esophageal squamous cell carcinoma (ESCC). By interacting with the long noncoding RNA LINC008866, SIRT7 deacetylates H3K18 on the promoter region of the transcription factor ELF3, finally suppressing EMT.^242^

## 3 SIRTUIN MODULATION: ACTIVATORS AND INHIBITORS

The multifaceted functions of SIRTs in aging, metabolism, neurodegeneration, and cancer stimulated the development of both sirtuin activators (SIRTa) and inhibitors (SIRTi). Indeed, depending on the specific pathology, it would be necessary to either activate or inhibit specific SIRT isoforms. To date, many SIRTa and SIRTi have been reported, some of them possessing pan‐sirtuin activity, while others exhibiting isoform‐selectivity. Most of them have been used as probes to understand sirtuin biology, but they may also serve as steppingstones for the creation of new drugs. In the next sections, we will illustrate the most significant SIRTa and SIRTi discovered to date.

### 3.1 SIRT activators

**Figure 2:**
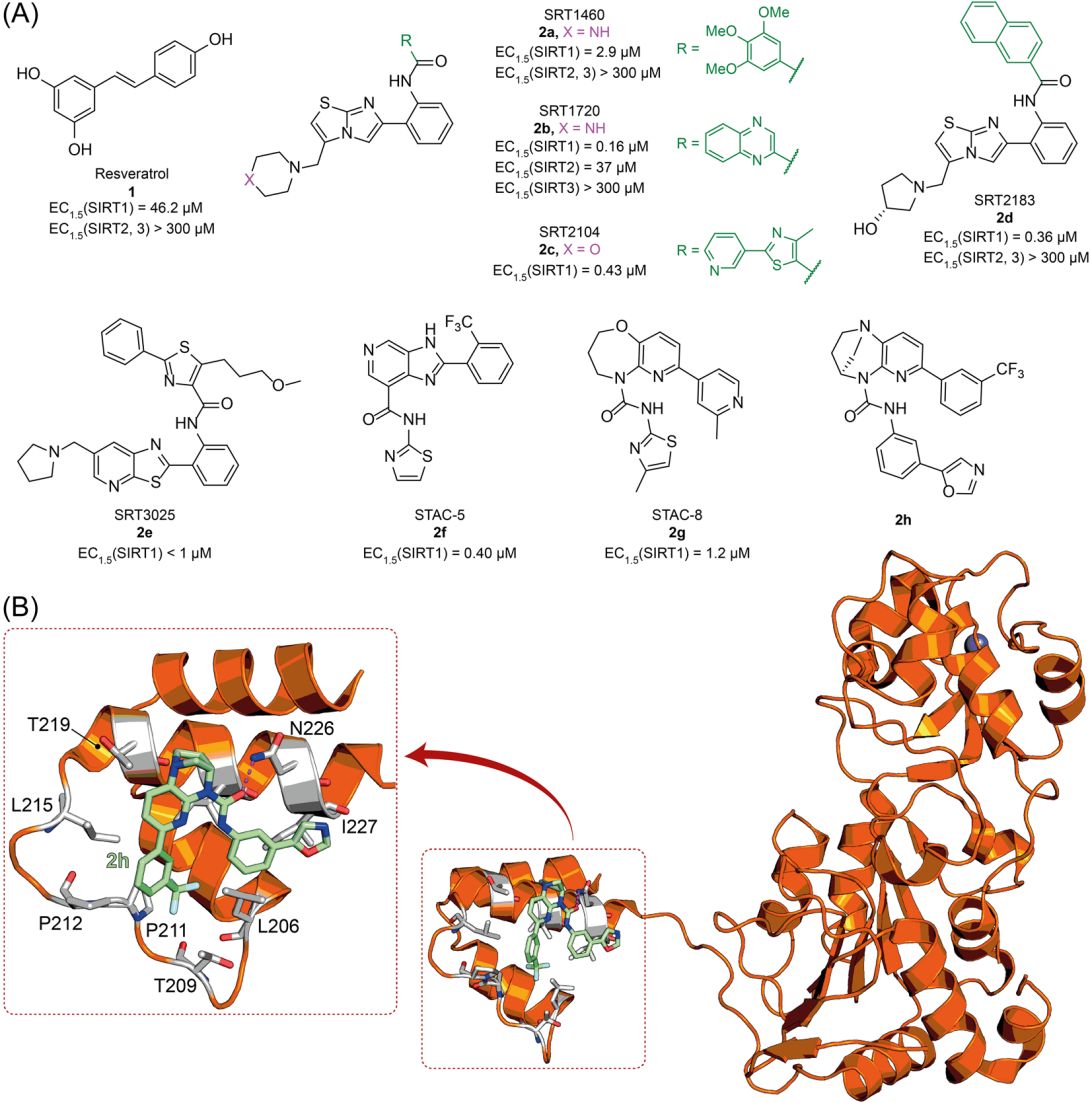
(A) Structures and SIRT modulation data of SIRT1a **1** and **2a‐h**. (B) Mini‐hSIRT1/**2h** co‐crystal structure (PDB ID: 4ZZH) showing the key interactions between the small molecule and the enzyme, including the carbonyl‐N226 hydrogen bond (purple dotted line). Mini‐hSIRT1 is colored in orange with key residues shown as white sticks, **2h** is depicted as green sticks. [Color figure can be viewed at wileyonlinelibrary.com]

The polyphenolic phytoalexin resveratrol (**1**, Figure 2A) is a naturally‐occurring molecule endowed with anti‐inflammatory, antioxidant, anticancer, and cardioprotective activities and it is the first SIRT1a described in literature.^243^ **1** allosterically activates SIRT1, increasing its activity by 50% (EC_1.5_) at 46.2 μM in an assay using an MR121 or TAMRA‐containing p53‐based substrate peptide^244^ and increases longevity in many organisms, including yeast and mammals. In mouse models, **1** enhances mitochondrial functions, exerting a protective role toward fat diet‐induced obesity.^245^ Many studies performed in humans indicate that **1** is highly absorbed after oral administration but quickly metabolized.^246^ It is indeed rapidly conjugated with glucoronate or sulfate in the liver.^247^ The sulfate‐conjugated metabolite, representing the main conjugated form of **1** can activate SIRT1 in vitro,^248^ thereby suggesting that the observed effects of **1** are mediated at least in part by this derivative. Different attempts have been made to improve the oral bioavailability of **1**.^249^ For instance, micronization was employed to obtain a formulation of **1**, called SRT501, characterized by particle size smaller than 5 µm. In an initial clinical trial, SRT501 was well tolerated in CRC patients and displayed a higher maximal plasma concentration (C_max_) than conventional **1**.^250^ Hence, SRT501 was tested in patients with refractory or relapsed multiple myeloma. However, this clinical trial was stopped because of severe adverse effects, such as nephrotoxicity.^251^ Over 70 clinical trials focusing on SIRT1 activation by **1** in a variety of medical conditions (e.g., cancer, metabolic, neurological, and cardiovascular disease) have been undertaken in recent years, with many of them still continuing.^249^ In the majority of completed trials, **1** demonstrated only neutral effects, with its bioavailability being a significant barrier. The only studies that found some benefits employed high doses of **1** (500 mg/day or above), with the greatest outcomes found in a Phase II study on individuals with coronary heart disease and type 2 diabetes.^252^ In this instance, treatment with **1** for 4 weeks (500 mg/day) substantially raised sensitivity to insulin as well as high‐density lipoprotein (HDL) blood levels relative to the placebo group.

It is essential to consider that the effects of **1** may also be attributed to its diverse and nonspecific modes of action. Nevertheless, **1** has stimulated the development of various small molecule sirtuin‐activating compounds (STACs). The most important STACs include SRT1460 (**2a**), SRT1720 (**2b**),^244^, ^253^ SRT2104 (**2c**),^254^ SRT2183 (**2d**), and SRT3025 (**2e**)^255^, ^256^ which selectively activate SIRT1 with EC_1.5_ values between 0.1 and 3 µM (Figure 2A) under the same assay conditions used for **1**. Compounds **2a‐e** were shown to improve glucose tolerance and sensitivity to insulin in diet‐induced and genetically obese mice. They also promoted lipidic metabolism and mitochondrial biogenesis, finally leading to weight decrease.^244^ Among them, **2c** has been assessed in different clinical trials. Disappointingly, Phase I studies indicated that **2c** has poor oral bioavailability^257^ and five out of eight trials investigating clinical responses demonstrated statistically insignificant or neutral outcomes.^249^ Nonetheless, a Phase I trial studying lipopolysaccharide‐triggered inflammation and coagulation indicated that **2c** (at doses of 500 or 2000 mg/day for 28 days) caused anticoagulant and anti‐inflammatory effects.^258^ At a dose of 2000 mg/day for 7 days, **2c** also decreased LDL and cholesterol levels in older subjects.^259^ Moreover, a Phase II clinical trial executed in psoriasis patients indicated that treatment with **2c** for 84 days (250–500–1000 mg/day), despite its inconstant pharmacokinetic profile, resulted in positive outcomes in 35% of patients.^260^ **2e** also underwent a Phase I trial to evaluate its safety and pharmacokinetics; nevertheless, the trial was halted after QT interval prolongation in different patients was observed, a warning indicator of probable lethal proarrhythmia induction.^261^

The precise mode of action of **1** and other STACs has been debated for a long time due to concerns about whether they directly bind and activate SIRT1.^262^, ^263^ In fact, different studies suggested that the measured SIRT1 activation was actually an artifact connected to the fluorophore‐containing peptides used as substrates in the assays.^264^ Subsequent reports established that the fluorescent groups were not required for SIRT1 activation and proved that both **1** and STACs bind SIRT1.^265^ Moreover, peptides obtained from native substrates of SIRT1 (e.g., FOXO3a, PGC‐1α), containing hydrophobic amino acids at positions corresponding to the fluorophores,^243^, ^244^ mediated SIRT1 activation by **1**, **2a**, STAC‐5 (**2f**), and STAC‐8 (**2g**) (Figure 2A).^266^ Hence, activation may depend on substrates, as also suggested by later studies,^267^ which explains the observed contrasting results.^262^, ^263^ By combining hydrogen‐deuterium exchange mass spectrometry (HDX‐MS) and X‐ray crystallography, Dai and colleagues recently demonstrated that STACs allosterically activate SIRT1. Specifically, they solved three structures of an engineered hSIRT1, called mini‐hSIRT1.^268^ In the first one, mini‐hSIRT1 is in complex with **2h**, an analog of **2g** bearing the urea bridging group; in a second structure, mini‐hSIRT1 binds **2h** and a SIRT1 substrate inhibitor; in a third one, mini‐hSIRT1 is bound to **2h**, carba‐NAD (nonhydrolyzable analog of NAD^+^), and an acetylated p53‐derived peptide.^268^ In line with the prerequisite for STACs to have a planar scaffold,^253^ the structure shows that **2h** interacts with an *N*‐terminal flat hydrophobic region, called STAC‐binding domain, with no known endogenous ligand. Specifically, **2h** forms a hydrogen bond with N226 and engages in hydrophobic contacts with L206, Y209, P211, P212, L215, Y219, and I223 (Figure 2B).^268^

**Figure 3:**
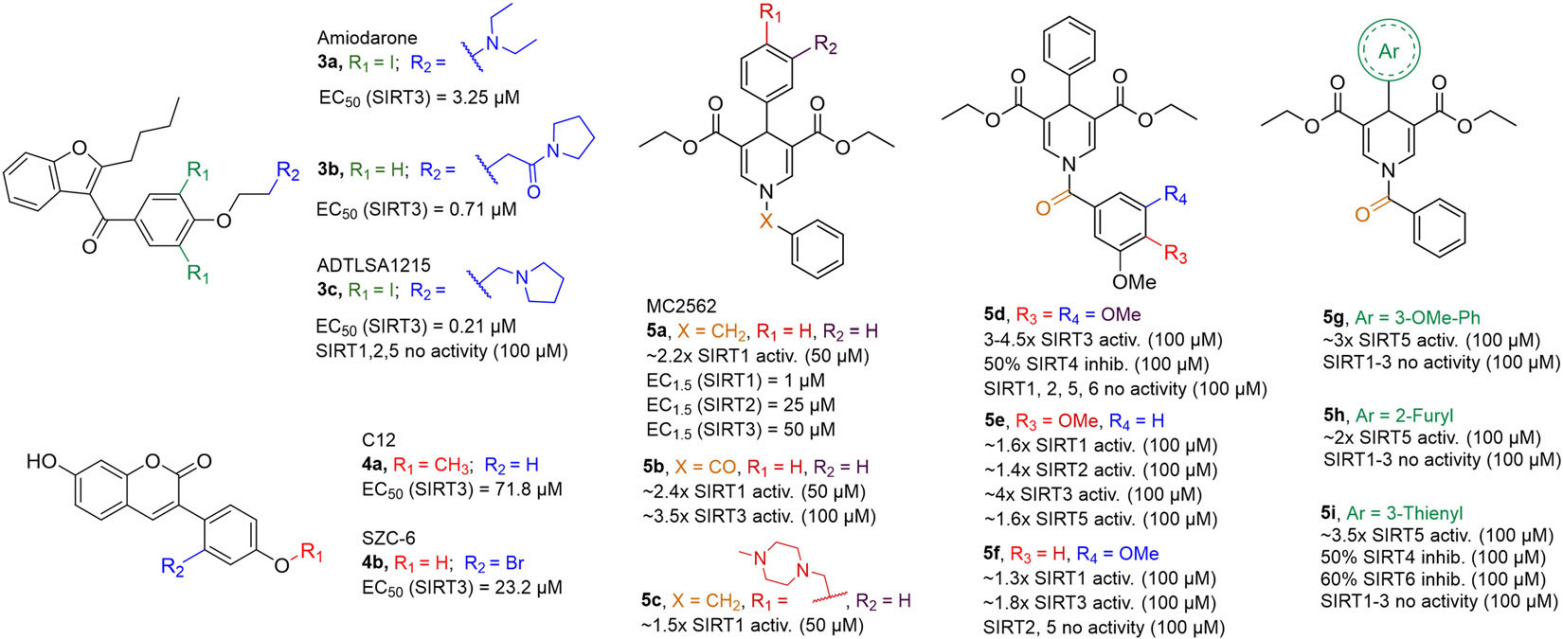
Structures and enzymatic activities of SIRT3a **3a‐c**, **4a,b**, and DHP‐based SIRT activators **5a‐i**. [Color figure can be viewed at wileyonlinelibrary.com]

A structure‐based approach recently led to the discovery of a series of 2‐butylbenzofuran‐based SIRT3 activators (**3a‐c**, Figure 3).^269^ Following docking experiments, Zhang et al. identified amiodarone (**3a**), a known antiarrhythmic drug, as lead molecule and solved the SIRT3/NAD^+^/peptide substrate/**3a** co‐crystal structure. Compound **3a** exhibited an EC_50_ value of 3.25 µM in a Fluor‐de‐Lys (FdL) SIRT3 activity assay using a fluoro‐acetylated peptide substrate based on residues 317‐320 of p53 (Gln‐Pro‐Lys‐Lys(Ac), with no further specification regarding the nature of the fluorophore). Compound **3a** was shown to form a stable complex with SIRT3 at the entrance of its acyl channel and was indicated to induce a conformational change by enhancing the π‐stacking between F157 and the nicotinamide moiety of NAD^+^, thereby bringing them closer to each other. The diethylamine tail engages in key interactions with F157, P176, I179, F180, and F294 and is pivotal for SIRT3 activation. Hence, different chains were explored to increase compound activity, leading to **3b** [EC_50_(SIRT3) = 0.71 µM in the FdL SIRT3 assay] in which the ethyl groups are conformationally restricted within a pyrrolidine ring and possessing a longer linker, while lacking the iodine atoms on the phenyl ring. Further modifications led to ADTL‐SA1215 (**3c**), possessing a propyl linker and in which the iodine atoms were reintroduced. **3c** exhibited an EC_50_ value of 0.21 µM in the same FdL assay, along with selectivity over SIRT1,2,5 (no activity at 100 µM). All three molecules were tested for their antiproliferative activity in triple negative breast cancer (TNBC) MDA‐MB‐231 cells, with **3c** being the most potent (IC_50_ = 2.19 µM, Table 1). **3c** also induced autophagy and mitophagy and impaired cancer cell migration. In the same cells, **3c** increased SIRT3 deacetylase activity and decreased acetylation of SOD2, a known SIRT3 substrate, while not affecting SIRT1,2,5 activity. Furthermore, a massive reduction or absence of antiproliferative activity of **3c** was observed in SIRT3‐knockdown MDA‐MB‐231 cells. Finally, **3c** decreased cancer cell proliferation in an MDA‐MB‐231 mouse xenograft, along with causing autophagy and decreasing the acetylation of whole cell extracts, mitochondrial extracts, and SOD2. However, at higher doses, **3c** caused significant lung toxicity, which is in line with known side effects of parent drug **3a**.^270^

**Table 1:** Most relevant SIRTa.

Compd.	Molecular structure	Enzymatic activity [substrate used]	Cell‐based/in vivo effects	References
**1**	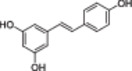	EC_1.5_ (SIRT1) = 46.2 µM [MR121 or TAMRA‐containing p53‐based peptide] EC_1.5_ (SIRT2, 3) > 300 µM	– Mice: enhancement of mitochondrial function and lifespan and protective role against obesity. – Type 2 diabetes and coronary heart disease patients: raise of HDL levels and insulin sensitivity.	[246, 250, 252, 253]
**2c** SRT2104	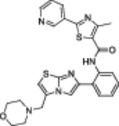	EC_1.5_ (SIRT1) = 0.43 µM [MR121 or TAMRA‐containing p53‐based peptide]	– Obese mice: higher insulin sensitivity and glucose tolerance; enhancement of mitochondrial biogenesis, modulation of lipid metabolism, weight loss. – LPS‐induced inflammation patients: anti‐inflammatory and anticoagulant activity. – Elderly patients: decrease LDL and cholesterol levels. – Psoriasis patients: encouraging outcomes in physician global assessment and histological evaluation in 35% of subjects.	[245, 256, 259, 260, 261, 408]
**3c** ADTL‐SA1215	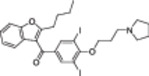	EC_50_(SIRT3) = 0.71 µM [fluoro‐acetylated peptide based on aa 317‐320 of p53 (Gln‐Pro‐Lys‐Lys(Ac))] SIRT1,2,5: no activity at 100 µM	– MDA‐MB‐231 breast cancer cells: increased SIRT3 activity and decreased SOD2 acetylation; antiproliferative activity (IC_50_ = 2.19 µM); induction of autophagy and mitophagy; impairment of cell migration. – MDA‐MB‐231 mouse xenograft: decreased acetylation of whole cell extracts, mitochondrial extracts and SOD2; cell proliferation suppression; autophagy induction; lung toxicity at high doses.	[270]
**5a** MC2562	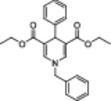	EC_1.5_ (SIRT1) = 1 µM EC_1.5_ (SIRT2) = 25 µM EC_1.5_ (SIRT3) = 50 µM [SIRT1: fluoro‐acetylated peptide based on aa 379‐382 of p53 (Arg‐His‐Lys‐Lys(Ac)SIRT2, 3: fluoro‐acetylated peptide based on aa 317‐320 of p53 (Gln‐Pro‐Lys‐Lys(Ac))]	– Mice: enhancement of wound repair. – HaCat cells: triggering of NO release. – AML U937 cells: low α‐tubulin acetylation. – Cancer cells panel: reduced acetylation of H4K16.	[276]
**5d**	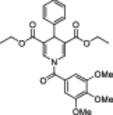	3‐4.5x SIRT3 activation at 100 µM [ACS2‐acK642 peptide] SIRT4: 50% inhibition at 100 µM SIRT1, 2, 5, 6: no activity at 100 µM	– TNBC cells: increased deacetylase activity, higher GDH activity, lower GDH acetylation in MDA‐MB‐231 cells.	[277]
**5 g**	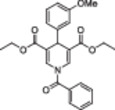	~3x SIRT5 activation at 100 µM [CPS1‐succK537 peptide] SIRT1‐3: no activity at 100 µM	– PDAC cells: reduced GOT1 acetylation, impaired cell viability (IC_50_s = 25.4–236.9 µM); synergistic activity with gemcitabine. – PDAC cells: synergistic activity with gemcitabine in PDAC cells and in vivo. – PDAC mouse PDX: combination with gemcitabine is tolerated and decreases tumor size, weight, and proliferation.	[277, 279]
**6a** UBCS039	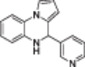	EC_50_ (SIRT6, deac.) = 38 µM 3.5x SIRT6 activation at 100 µM [Acetylated H3K9 peptide] ~ 2x SIRT5 activ. At 100 µM SIRT1‐3 no activity at 100 µM	– NSCLC, fibrosarcoma, colon and epithelial cervix carcinoma: SIRT6 activation and reduced acetylation of H3K9 and H3K56, along with autophagy‐related cell death. – LPS‐treated HPMEC: downregulation of the adhesion protein VCAM1 and impaired monocyte adhesion.	[282, 283]
**7a** MDL‐800	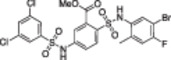	EC_50_ (SIRT6, deac.) = 10.3 μM ×22 SIRT6 activation at 100 µM [AMC‐containing acetylated peptide (RHKK‐ac‐AMC)] IC_50_ (SIRT2, 5, 7) = 100–187 µM SIRT1,3: no activity at 100 µM SIRT4: no activity at 50 µM	– HCC and NSCLC cells: decrease of of H3K9 and H3K56 acetylation and consequent cell cycle arrest. – HCC mouse xenografts: tumor growth inhibition. – Chondrocytes: reduction of senescence and osteoarthritis progression.	[198, 199, 286]
**7c** MDL‐811	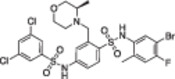	EC_50_ (SIRT6, deac.) = 5.7 µM [AMC‐containing acetylated peptide (RHKK‐ac‐AMC)] SIRT1‐3, 5, 7 no activity at 200 µM	– CRC cells: decrease of H3K9, H3K18, and H3K56 acetylation; proliferation inhibition linked to cell cycle arrest at G0/G1. – CRC PDO, PDX and spontaneous CRC mouse model: cancer growth inhibition.	[292]
**8b**	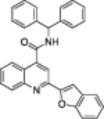	EC_1.5_ (SIRT6, deac.) = 0.58 µM EC_1.5_ (SIRT6, demyr.) = 0.72 µM EC_50_ (SIRT6, deac.) = 5.35 µM EC_50_ (SIRT6, demyr.) = 8.91 µM [AMC‐containing acetylated peptide (Ac‐RYQK(Ac)‐AMC) and AMC‐containing mirystoylated peptide Ac‐EALPKK(Myr)‐AMC] IC_50_ (SIRT1) = 171.20 µM IC_50_ (SIRT2,3,5) > 200 µM	– PDAC cells: proliferation impairment and cell cycle arrest in G2 phase. – PDAC xenograft mouse model: tumor growth inhibition and reduction of acetylation at H3K9.	[293]

Abbreviations: CRC, colorectal cancer; HCC, hepatocellular carcinoma; HDL, high‐density lipoprotein; HPMEC, human pulmonary microvascular endothelial cells; LDL, low‐density lipoprotein; LPS, lipopolysaccharide; NSCLC, non‐small cell lung cancer; PDAC, pancreatic ductal adenocarcinoma; PDO, patient‐derived organoid; PDX, patient‐derived xenograft; TNBC, triple‐negative breast cancer.

Lu et al. reported the discovery of the coumarin‐based C12 (**4a**) as a SIRT3a, showing that it can promote SOD2 deacetylation at K68 in both enzymatic and cell‐based assays. The authors measured the SIRT3‐**4a** dissociation constant via ITC (*K*_*d*_ = 3.9 µM) but did not evaluate the activation potential or isoform selectivity (Figure 3).^271^ Based on this compound, Li and colleagues reported the derivative SZC‐6 (**4b**, Figure 3), characterized by the presence of a bromine moiety at C2 of ring C, as well as an hydroxyl group replacing the methoxy one at C4. Surface plasmon resonance (SPR) experiments indicated a *K*_*d*_ value of 15 µM for **4b**, compared with 24.3 µM for **4a** in the same assay.^272^ The authors measured EC_50_ values for both compounds in a SIRT3 deacetylase activity assay using a fluoro‐acetylated substrate peptide (with no further specification regarding the nature of the fluorophore or sequence), showing that **4b** is threefold more potent than **4a** [EC_50_(**4a**) = 71.8 µM; EC_50_(**4a**) = 23.2 µM]. Moreover, both **4a** and **4b** increased SIRT3 activity by >20‐fold at 100 µM. Compound **4b** was tested in primary cultures of neonatal cardiomyocytes and decreased mitochondrial protein acetylation, including SOD2 without affecting SIRT3 levels. Moreover, **4b** dose‐dependently mitigated the stress‐induced hypertrophic response in the same cells at concentrations between 10 and 40 µM.^272^ Finally, **4b** improved mitochondrial function in mouse models and reduced cardiac hypertrophy, cardiac fibroblast proliferation, and differentiation into myofibroblasts. Nevertheless, selectivity over other isoforms was not tested. Given the coumarin‐based structure, these compounds may also target monoamine oxidase B (MAO‐B), thus contributing to their cellular and in vivo cardiac effects.^273^

MC2562 (**5a**, Figure 3) is a 1,4‐dihydropyridine (DHP) displaying sirtuin activation with EC_1.5_ values of 1, 2.5, and 50 µM for SIRT1, 2, and 3, respectively in FdL fluorescent‐based assays (Table 1), and capable of increasing SIRT1 activity by 2.2‐fold at 50 µM.^274^, ^275^ **5a** was shown to reduce the acetylation of H4K16 in different cancer cell lines and α‐tubulin in leukemia U937 cells, it also triggered NO secretion in HaCat keratinocytes, and promoted wound healing in mice.^275^ **5b**, a derivative of **5a** in which the N1 benzyl is changed to a benzoyl moiety, was able to activate SIRT1 by 2.4‐fold at 50 µM and inhibit the proliferation of CRC cell line LoVo with an IC_50_ = 22 µM. **5c** is the water‐soluble form of **5a**, in which the phenyl ring is replaced by a 4‐(4‐methylpiperazyn‐1‐yl)methylphenyl dihydrochloride group. **5c** enhances SIRT1 activity by 1.5‐fold at 50 µM and exerts antiproliferative activity in many cancer cell lines (IC_50_s = 8–35 μM).^275^

Recent studies uncovered the potential of 1,4‐DHP derivatives as SIRT3 activators. Suenkel et al. reported that compounds **5b** and the newly‐reported **5d**, bearing a 3,4,5‐trimethoxybenzoyl group at N1, increase SIRT3 deacetylase activity by ~3.5 and ~4.5‐fold, respectively, at 100 µM (Figure 3). The assay used in this case was not fluorescence‐based, but rather consisted in a nicotinamidase (PncA)/GDH‐coupled deacylation assay which measured the production of NADP^+^ from NADPH oxidation and used an acetyl‐CoA synthetase 2 (ACS2)‐acK642 SIRT3‐specific substrate peptide.^276^ Compound **5d** was selective over SIRT1, 2, 5, and 6 at 100 µM, but inhibited 50% SIRT4 activity at 100 µM. Moreover, **5d** displayed a *K*_d_ value of 32 µM in a microscale thermophoresis (MST) experiment, while no EC_50_ could be calculated due to solubility limitations. Notably, following a 3 h incubation in TNBC MDA‐MB‐231 cells, both **5b** and **5d** increased the deacetylase activity of cell lysates at 50 and 100 µM. They could also increase GDH activity and decrease its acetylation status, in line with experiments performed following SIRT3 overexpression.^276^ A subsequent study identified compounds **5e** and **5f**, bearing a 3,4‐ or 3,5‐ dimethoxybenzoyl group at N1, respectively (Figure 3), as new SIRT3 activators under the same assay conditions. **5e** and **5f** increased SIRT3 deacetylase activity by ~4‐ and ~1.8‐fold at 100 µM, while **5d** could increase SIRT3 activity by only ~3‐fold in this case.^277^ When tested against SIRT1, 2, and 5 at 100 μM, **5e** increased their activity by maximum 1.6‐fold, while **5f** could activate SIRT1 by 1.3‐fold. Hence, considering the SIRT3 activating potential we can conclude that **5e**, but not **5f**, is a SIRT3‐selective activator. SPR experiments yielded *K*_d_ values of 41, 29, and 79 µM for **5d**, **5e**, and **5f**, respectively, and all three compounds increased GDH activity by ~1.5‐fold in TNBC MDA‐MB‐231 cells at 50 µM. When tested in MDA‐MB‐231 and thyroid anaplastic carcinoma CAL‐62 cells at 50 µM, both **5e** and **5f** induced time‐dependent decrease of cell viability in both normoxia and hypoxia conditions, with **5f** being the most potent. Moreover, both compounds significantly decreased MDA‐MB‐231 cell migration in a scratch assay, with **5f** being again more effective.^277^

Suenkel and colleagues also reported 1,4‐DHP derivatives endowed with SIRT5 activating properties. Among them, they identified compounds **5g**, **5h**, and **5i** as SIRT5 activators in a PncA/GDH‐coupled deacylation assay using CPS1‐succK537 peptide as SIRT5 substrate. These compounds are derivatives of **5b** bearing a 3‐methoxyphenyl, 2‐furyl, and 3‐thienyl moiety at C4 (Figure 3).^276^ They increased SIRT5 desuccinylase activity by 1.5–2‐fold at 10 µM and by 2–3.5‐fold at 100 µM, and **5i** exhibited an EC_50_ value of 40 μM. Compounds **5g**, **5h**, and **5i** were selective over SIRT1‐3 at 100 µM. **5i** was also tested against SIRT4 and SIRT6, demonstrating ~50% and ~60% inhibition, respectively. **5g** and **5i** were also assessed in TNBC MDA‐MB‐231 cells at 50 µM. After 4 and 24 h incubation they both decreased the activity of glutaminase, a SIRT5 substrate whose activity is dependent on the succinylation status.^142^ Likewise, the administration of **5g** and **5i** to PDAC cells S2‐013 and Capan1 at 20 µM for 24 h decreased the acetylation of the SIRT5 substrate GOT1. Conversely, mouse SIRT5‒KO cells KPCS exhibited no apparent effects.^276^ These results are in line with those described by Hu and colleagues.^278^ They showed that treatment with **5g** reduced PDAC cancer cell viability (IC_50_s = 25.4–236.9 µM) and promoted protein deacetylation, analogously to SIRT5 overexpression. Among SIRT5 substrates, **5g** was able to reduce the acetylation levels GOT1 and its enzymatic activity. In addition, the **5g**‐gemcitabine combination exhibited synergistic effects both in vitro and in vivo, where it decreased tumor size, weight, and proliferation. This combination was also well tolerated in PDAC patient‐derived xenograft (PDX) mice models.^278^

**Figure 4:**
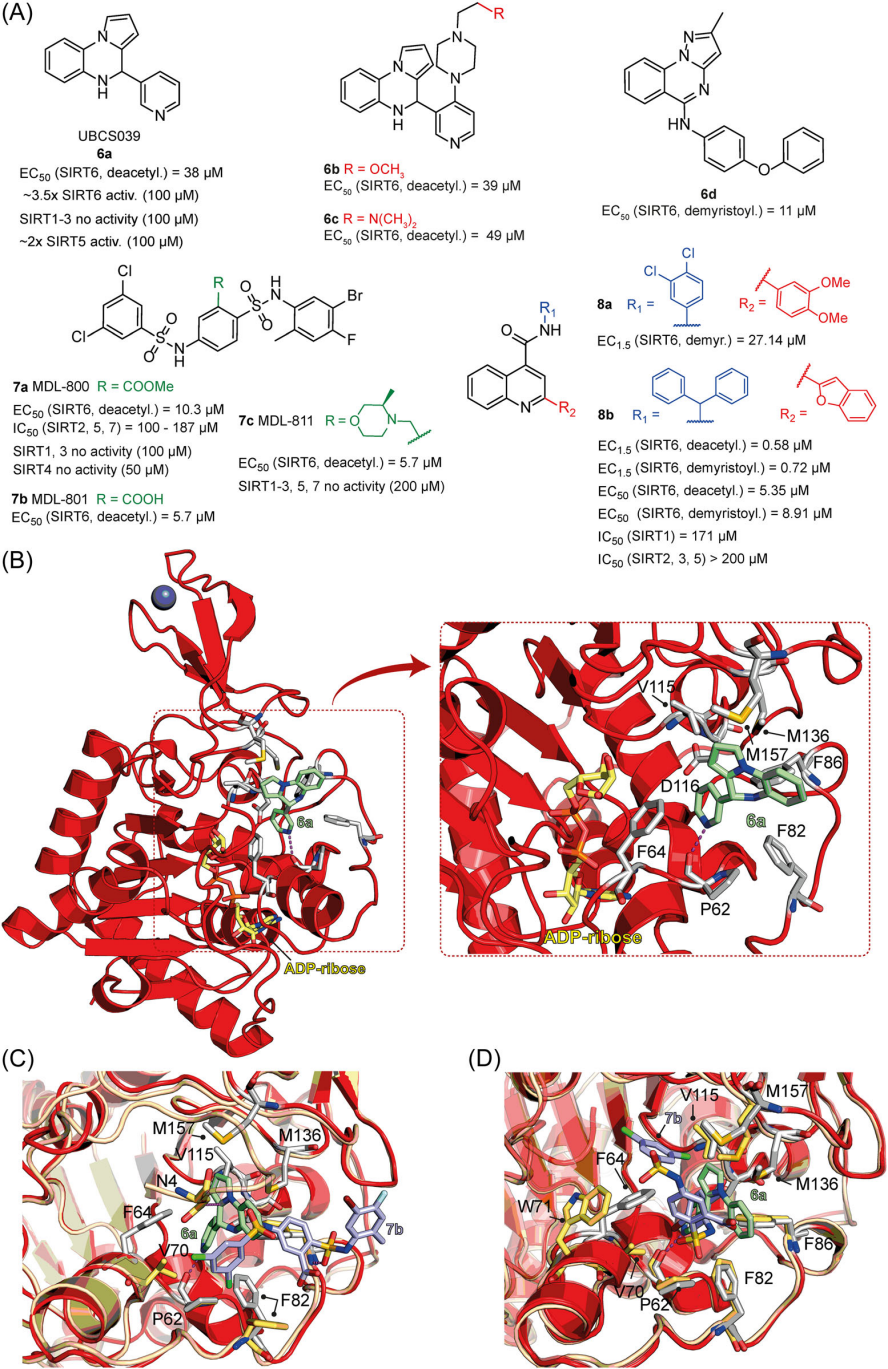
(A) Structures and enzymatic activities of SIRT6a **6a‐d**, **7a‐c**, and **8a,b**. (B) hSIRT6/ADP‐ribose/**6a** co‐crystal structure (PDB ID: 5MF6) showing the key interactions between the small molecule and the enzyme, including the pyridine nitrogen‐P62 hydrogen bond (purple dotted line). (C, D) Superimposition of the co‐crystal structures of hSIRT6 bound to **6a** (PDB ID: 5MF6) or **7b** as reported by as reported by Huang et al. (PDB ID: 5Y2F) (C) or by You and Steegborn (PDB ID: 6XVG) (D). Key residues for compounds' binding are labeled, and polar interactions are shown as dashed magenta lines. hSIRT6 is colored in red with key residues shown as white sticks. Compound **6a** is depicted as green sticks, compound **7b** is depicted as light blue sticks, and ADP‐ribose is depicted as yellow sticks. [Color figure can be viewed at wileyonlinelibrary.com]

UBCS039 (6a, Figure 4A) is the earliest reported synthetic SIRT6a, increasing its deacetylase activity by up to 3.5‐fold at 100 µM and displaying an EC_50_ value of 38 µM in a continuous microplate assay^279^ using an acetylated H3K9 peptide as substrate. 6a was tested as a racemate and could also increase SIRT5 activity twofold at 100 µM, while being selective over SIRT1‐3. The co‐crystal structure of SIRT6 bound to 6a and ADP‐ribose (Figure 4B) indicates that the molecule is placed at the end of the acyl channel, a site where the fatty acyl chains of acylated substrates usually reside. Specifically, the tricyclic core of 6a engages in an aryl group–methionine interaction with M136 and forms hydrophobic interactions with Y71, F82, F86, I185, and M157. The pyridine nitrogen of 6a engages in a key hydrogen bond with P62 backbone carbonyl, while the benzene ring belonging to the quinoxaline core is exposed to the solvent (Figure 4B).^280^ At 100 µM concentration, 6a reduced H3K18 acetylation of physiological substrates such as full‐length histones and HeLa nucleosomes. Moreover, 6a can increase SIRT6 activity, reduce acetylation of H3K9/K56 and stimulate autophagy‐driven apoptosis in different cancer cells, including fibrosarcoma, epithelial cervix carcinoma, CRC, and NSCLC.^281^ Compound **6a** was also recently shown to reduce the inflammatory response in lipopolysaccharide (LPS)‐treated human pulmonary lung microvascular endothelial cells (HPMEC) by decreasing the expression of the adhesion protein VCAM1 and impairing monocyte adhesion.^282^

The discovery of a binding site specific to SIRT6 that could be exploited for activation prompted additional investigations, culminating in the generation of an array of **6a** derivatives that share the pyrrolo[1,2‐*a*]quinoxaline nucleus. Compounds **6b** and **6c**, which have a piperazinyl group linked to position C4 of the pyridine ring^283^ demonstrated the greatest activities in a SIRT6 fluorescence‐based FdL deacetylation assay with EC_50_ values of 38.77 and 48.75 µM, respectively, along with ~6‐ and ~8‐fold activation at 100 µM (Figure 4A). Notably, both compounds were selective over SIRT1‐3 and 5 at 100 µM. Docking studies suggest that **6c** interacts with W188 in a conformation distinct from that of **6a** and retains the pyridine ring oriented away from P62. Additionally, the side chain's protonated nitrogen of the dimethylamino group engages in π‐cation interactions with W188. **6b** was also demonstrated to promote H3K9 deacetylation mediated by SIRT6 in NSCLC (H1299) and HCC (PLC/PRF/5) cell lines and decreased colony formation by 50% at 30 µM. Moreover, **6a** and its derivatives **6b** and **6c** all reduced the expression of pro‐inflammatory genes in LPS‐stimulated BV2 cells.^283^

A docking study based on the SIRT6‐**6a** co‐crystal structure recently led to the development of pyrazolo[1,5‐*a*]quinazoline derivatives among which compound **6d** (Figure 4A) exhibited an EC_50_ value of 11.15 µM and an EC_1.5_ value of 1.85 µM in an FdL‐based SIRT6 demyristoylation assay using a myrisoylated peptide bearing the 7‐amino‐4‐methylcoumarin (AMC) fluorophore (Ac‐EALPKK(Myr)‐AMC). Furthermore, cellular thermal shift assay (CETSA) performed in mouse embryonic fibroblasts (MEFs) demonstrated that **6d** had the ability to stabilize SIRT6, thus suggesting cellular target engagement.^284^

Recently, Huang et al. reported the bis‐benzenesulfonamide MDL‐800 (**7a**) and the corresponding carboxylic acid MDL‐801 (**7b**) as low‐micromolar SIRT6a in an FdL assay employing an AMC‐containing acetylated peptide substrate (RHKK‐ac‐AMC) [EC_50_(**7a**) = 10.3 µM, EC_50_(**7b**) = 5.7 μM, Figure 4A].^198^ **7a** was inactive toward HDAC1‐11 and SIRT1,3,4 and displayed negligible SIRT2 inhibition (IC_50_ = 100.4 μM) along with little SIRT5 and SIRT7 activation (EC_50_ values of 104.6 and 187.1 μM, respectively). Both **7a** and **7b** enhanced SIRT6 activity by more than 22 times at 100 µM and increased the acetylation of nucleosomes in a dose‐dependent manner. Moreover, HPLC experiments indicated that **7b**‐induced SIRT6 deacetylation was not reversed by myristic acid or compound **6a**. Notably, addition of **6a** further increased **7b**‐mediated SIRT6 activation at a concentration of 100 μM of each compound. Overall, these data led the authors to conclude that the two compounds possess different binding sites. Given the high cellular efflux ratio and poor permeability of **7b**, only **7a** was assessed in cancer cells. Specifically, **7a** reduced the acetylation of H3K9 and H3K56, induced cell cycle arrest in HCC cells, and was able to stop the proliferation of both HCC and NSCLC cell lines. Compound **7a** suppressed tumor growth in both HCC and adenocarcinoma xenograft mouse models.^198^, ^285^ Furthermore, **7a** was recently shown to reduce diastolic dysfunction and cardiac lipid accumulation in diabetic mice^286^ and exert anti‐inflammatory, angiogenetic, and wound‐healing action in mouse models.^287^ Recently, compound **7a** was employed as a chemical tool by Ji and colleagues. Specifically, they encapsulated **7a** (at a concentration of 5, 10, and 20 μM) in Cy5.5 labeled‐tgg2‐functionalized PEGylated polyamidoamine (PAMAM) nanoparticles and demonstrated that the formulation containing **7a** at a concentration of 10 μM could reduce chondrocyte senescence and the progression of osteoarthritis.^197^ In another study, compound **7b** was administered to WT and *SIRT6* KO mice at a dose of 100 mg/kg via oral gavage once a day over 4 weeks during a treadmill exercise program. Treatment with **7b** resulted in reduced H3K9 acetylation in the muscle tissues of WT mice, while no effects were observed in *SIRT6* KO mice. Moreover, WT mice treated with **7b** exhibited a higher proportion of slow fibers as well as increased mitochondrial oxidative capacity. These data are in line with SIRT6‐dependent increased expression of *Creb1* and concomitant decreased of *Sox6* expression, thus suggesting that SIRT6 activation may aid cellular reprogramming and adaptation to endurance exercise.^288^

The co‐crystal structure of SIRT6 bound to a H3K9 myristoyl peptide, ADP‐ribose, and **7b**, shows that the molecule binds to a distal region of the acyl‐binding channel, distinct from the binding pocket of **6a** (Figure 4C).^285^ Conversely, a co‐crystal structure of SIRT6 bound to ADP‐ribose and **7b** reported by the Steegborn group suggests that **7b** does instead bind in the internal region of the acyl‐binding channel (Figure 4D).^289^ Consequently, Huang et al. replicated the experiments and still obtained the same crystal structure as the originally published one.^290^ Overall, these differences are probably a consequence of the different conditions employed for crystallization (e.g., the use of the substrate peptide by Huang and colleagues, but not by the Steegborn's team), and the structures may represent two alternative protein conformations.

MDL‐811 (**7c**), a **7a** analog with an *N*‐methyl‐3‐methylmorpholine instead of a methyl carboxylate group (Figure 4A), displayed an EC_50_ value of 5.7 µM in the same FdL SIRT6 deacetylation assay as **7a, b** and was selective over SIRT1‐3, 5, 7, and HDAC1‐11.^291^ When tested in CRC cells, **7c** reduced H3K9, H3K18, and H3K56 acetylation and induced cell cycle arrest at G0/G1 along with inhibition of cell proliferation (IC_50_s = 4.7–61.0 μM). **7c** also impaired CRC growth in a mouse spontaneous CRC model, in PDX, and in patient‐derived organoids (PDO).^291^

Compound **8a** [EC_1.5_(SIRT6, demyristoylation) = 27.14 μM, Figure 4A] is a quinoline‐4‐carboxamide derivative identified following a docking analysis employing the SIRT6‐**6a** co‐crystal structure (PDB ID: 5MF6) as model. According to docking experiments, **8a** binds at the distal portion of the hydrophobic channel in which the quinoline ring engages in π − π contacts with F86 and the 3,4‐dichlorobenzene moiety seems to interact with the amide backbone of A7 via σ − π interactions. Since some space close to the 3,4‐dichlorobenzene and 3,4‐dimethoxybenzene groups appeared not to be occupied, the structure of **8a** was modified to reinforce the interactions with SIRT6. This led to **8b**, in which the 3,4‐dichlorobenzene and the 3,4‐dimethoxybenzene are substituted by a diphenylmethane moiety and a 2‐benzofuranyl portion, respectively (Figure 4A). **8b** is a potent and selective SIRT6a with EC_1.5_s of 0.58 μM (deacetylation) and 0.72 μM (demyristoylation), and EC_50_s equal to 5.35 μM (deacetylation) and 8.91 μM (demyristoylation).^292^ The two assays were both FdL‐based and used different AMC‐containing peptide substrates for the deacetylation [Ac‐RYQK(Ac)‐AMC] and the demyristoylation [Ac‐EALPKK(Myr)‐AMC] reaction evaluation. Compound **8b** displayed weak SIRT1 inhibition (IC_50_ = 171 µM) and did not affect the activity of SIRT2,3,5, HDAC1‐11, and 415 kinases. **8b** suppressed migration and proliferation in a panel of PDAC cells (IC_50_ values = 4.1–9.7 μM) and CETSA executed in PANC‐1 and BXPC‐3 PDAC cells validated **8b** target engagement. Furthermore, **8b** stopped tumor growth in a PDAC mouse xenograft model.^292^

The most important sirtuin activators are indicated in Table 1.

### 3.2 SIRT inhibitors

**Figure 5:**
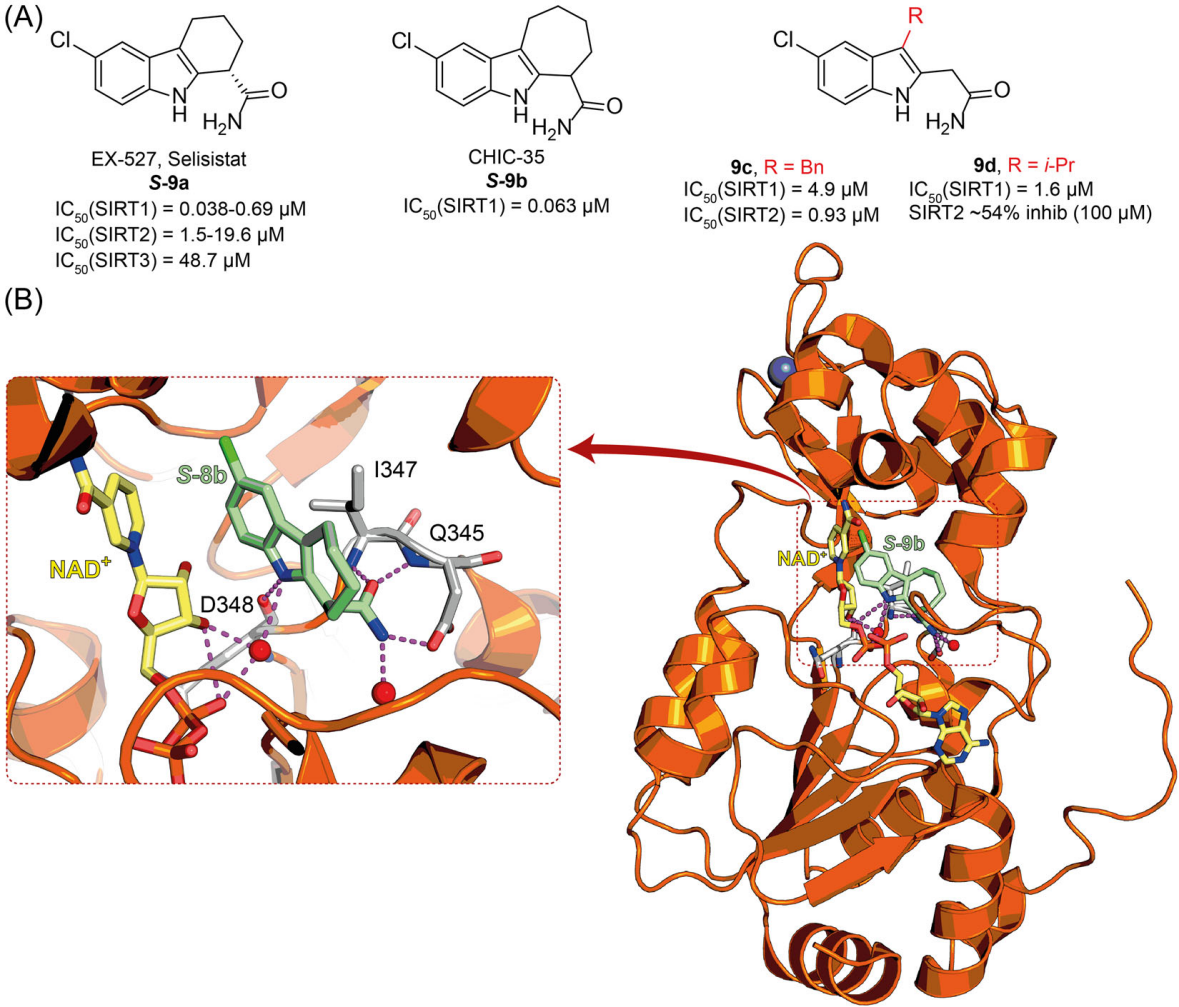
(A) Structures and enzymatic activities of compounds **9a‐d**. (B) SIRT1/CHIC‐35 (***S‐*** **9b**) co‐crystal structure (PDB ID: 4I5I) showing the key interaction between the small molecule (green sticks) and the enzyme (orange cartoon, with key residues shown as white sticks). Hydrogen bonds involving ***S‐*** **9b**, Q345, I347, D348, NAD^+^ (yellow sticks), and conserved water molecules (red spheres) are depicted as purple dotted lines. [Color figure can be viewed at wileyonlinelibrary.com]

EX‐527 (Selisistat, **9a**, Figure 5A) is the first submicromolar SIRT1i endowed with cell permeability to be described. **9a** possesses a stereogenic center, with its *S*‐enantiomer (***S*** **−9a**) being the eutomer [(IC_50_ (***S*** **−9a**) = 0.12 µM; IC_50_ (***R*** **−9a**) > 100 µM]. Initial assays indicated strong selectivity of **9a** over SIRT2 (IC_50_ = 19.6 μM), SIRT3 (IC_50_ = 48.7 μM), class I‐II HDACs, and NAD^+^ glycohydrolase.^293^ Nonetheless, subsequent studies suggested that **9a** potency and selectivity are substrate‐dependent. Specifically, one study indicated that the IC_50_(SIRT1) value was 0.26 µM while the IC_50_(SIRT2) was 2.9 µM.^294^ Subsequently, Solomon et al. measured an IC_50_ value of 0.038 μM against SIRT1,^295^ while Therrien et al. obtained the following values: IC_50_(SIRT1) = 0.5 µM and IC_50_(SIRT2) = 6.5 µM ^296^ and Laaroussi et al.^297^ indicated only twofold selectivity over SIRT2, with IC_50_(SIRT1) being 0.69 µM and IC_50_(SIRT2) being 1.5 µM.

Mechanistically, **9a** seems to interact with the C‐pocket of SIRT1 and a neighboring hydrophobic area. According to kinetic and structural studies, **9a** interacts with SIRT1 after the formation of the alkylimidate intermediate. Binding of **9a** prevents the release of 2′‐*O*‐acetyl‐ADP‐ribose and generates a stable inhibitory complex with SIRT1.^298^ The co‐crystal structure of the catalytic domain of human SIRT1 bound to NAD^+^ and **9b**, a derivative of **9a**, shows that the *S*‐enantiomer of **9b** (***S*** **−9b**, also called CHIC‐35) does indeed bind inside the catalytic pocket (Figure 5B). ***S*** **−9b** engages in hydrophobic interactions and hydrogen bonds such as those between the side chain and the backbone amide of D348 and the primary amide of the inhibitor. This amide also interacts with the backbone amide of I347 through its oxygen atom and with a conserved water molecule through the NH_2_ group. The indole ‐NH also forms hydrogen bonds with Q345 backbone oxygen and with another water molecule which in turn is coordinated by two oxygens from the ribose and phosphate portions of NAD^+^. From the structure, ***S*** **−9b** appears to dislodge NAD^+^ nicotinamide and drive it into an expanded conformation, preventing substrate interaction with the enzyme. However, this proposed mechanism does not entirely match kinetic experiments.^299^

Compound **9a** has been shown to raise the levels of acetylated p53 in various cancer cell lines, consistent with the fact that SIRT1 deacetylates p53 at Lys382. To the best of our knowledge, no further studies were performed to demonstrate target engagement with SIRT1.^295^, ^300^ In terms of anticancer activity, **9a** was shown to increase the activity of cytotoxic drugs.^300^ For instance, it decreased cell survival and migration of HCC cell lines HepG2 and Huh7.^301^ In addition, it impaired migration and EMT of chemotherapy‐resistant esophageal cancer cells^302^ and reduced colony formation of ovarian carcinoma cells.^303^ When tested in vivo, **9a** was able to reduce tumor growth of both lung^304^ and endometrial cancer^305^ xenograft mouse models. Interestingly, although **9a** amplified the cytotoxic effects of gemcitabine in PANC‐1 human pancreatic cancer cells,^306^, ^307^ it promoted tumor development in a xenograft mouse model of pancreatic cancer.^307^ The authors hypothesized that SIRT1 inhibition in other cells within the cancer microenvironment (i.e., fibroblasts, endothelial, and immune cells) may have been involved in the tumor‐promoting activity of **9a**. Nonetheless, this is just a hypothesis that needs to be experimentally validated.

Recent reports have indicated that **9a** modulates the acetylation status of the mutant huntingtin through SIRT1 inhibition, thus leading to a protective role in the onset of HD.^57^ Furthermore, **9a** exhibited good bioavailability, cell permeability, and metabolic stability,^293^ thereby stimulating its assessment in various clinical trials, two of which have been recently completed. The first Phase I clinical trial indicated that **9a** is well tolerated in healthy subjects up to the maximum 600 mg single dose regimen or multiple doses of 300 mg/day.^308^ Another Phase I clinical trial executed in early‐stage HD patients indicated that **9a** is safe at 10 and 100 mg/day and could provide clinical, cognitive, and neuropsychiatric progress from baseline (Day −1) to Day 1, with no additional improvements at Day 14.^309^ Two more clinical trials, specifically a Phase I and a Phase II study, have been performed to investigate the use of **9a** in HD (NCT01485965 and NCT01521585), but the results have not been published yet. Given the involvement of SIRT1 overexpression in the pathogenesis of endometriosis,^310^ a new clinical trial was expected to start in January 2022 for the evaluation of **9a** as a possible treatment of the inflammation associated with endometriosis and of the endometriosis‐mediated in vitro fertilization failure (NCT04184323), but it has been withdrawn due to lack of funding.

Compounds **9c,d** are the result of structure and ligand‐based optimization efforts aimed at obtaining achiral derivatives of **9a**.^297^ To this end, the cycloalkyl ring of **9a,b** was opened, while keeping the carboxyamide moiety crucial for SIRT1 binding. From this study, substitution at C3 of the indole ring seemed to affect compound activity and selectivity. Hence, the optimization of this portion led to **9c**, carrying a benzyl moiety at indole C3, and **9d** which instead has an isopropyl moiety at the same position (Figure 5A). Notably, in **9c** the SIRT1 selectivity was completely lost, with the compound being more potent toward SIRT2 as it exhibited IC_50_ values of 4.9 and 0.93 µM for SIRT1 and SIRT2, respectively. Conversely, in **9d** the SIRT1 selectivity was restored since the compound displayed an IC_50_(SIRT1) of 1.6 µM while only 54% SIRT2 inhibition was observed at 100 µM. Hence, it is apparent that bulky aromatic substitutions at indole C3 shift the activity toward SIRT2. At cellular level, **9c** was more potent than **9a** in terms of cytotoxicity toward HCT‐116 and HT‐29 CRC cell lines, however, target engagement was not investigated.^297^

**Figure 6:**
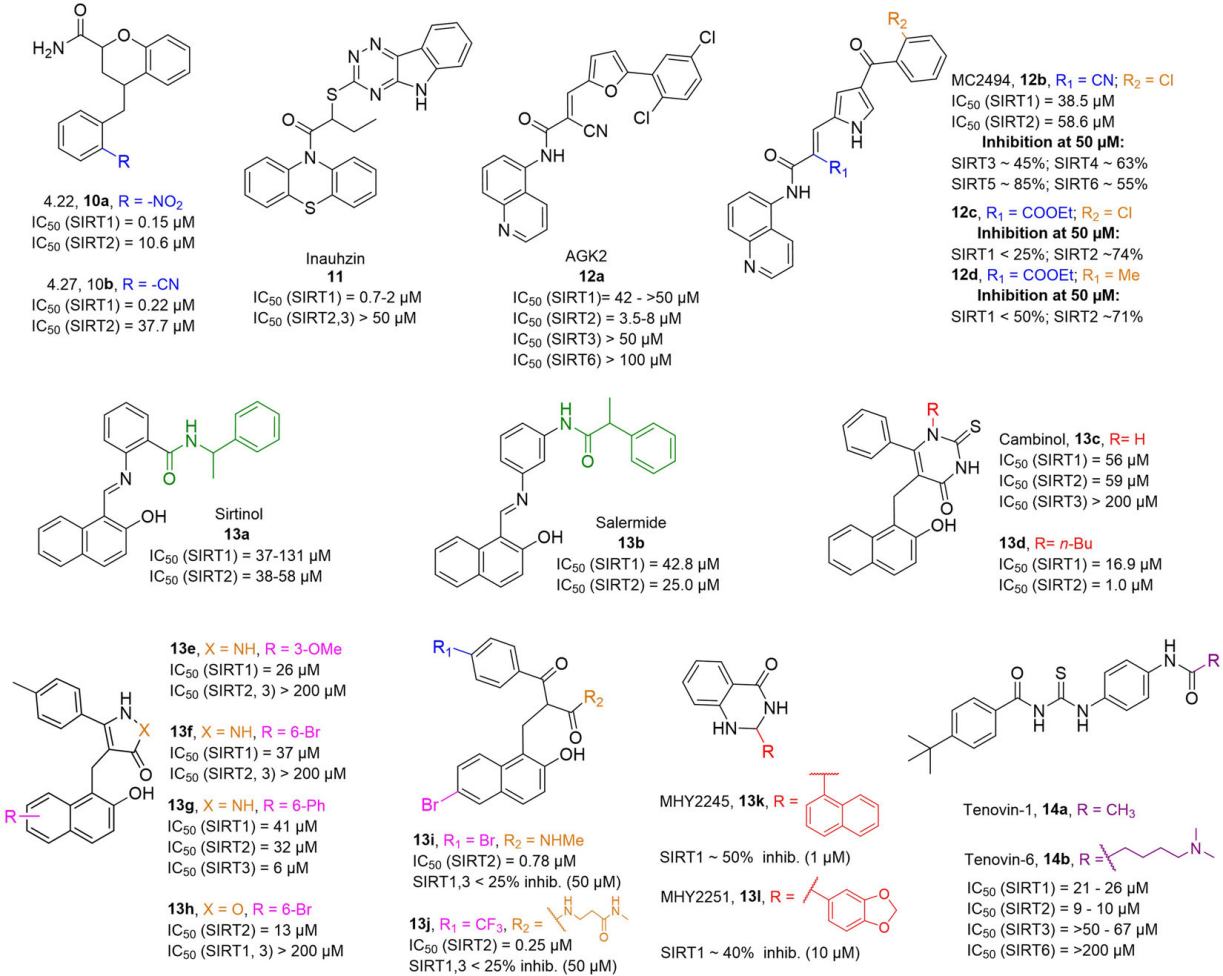
Structures and enzymatic activities of SIRT1/2 inhibitors **10a,b**, **11**, **12a‐d**, sirtinol (**13a**) and its related compounds (**13b‐l**), and Tenovin‐1/6 (**14a,b**). [Color figure can be viewed at wileyonlinelibrary.com]

Recently, Spinck et al. reported a group of dihydro‐1,4‐benzoxazine carboxamides as highly potent and selective inhibitors of SIRT1 (Figure 6).^311^ Among the screened molecules, 4.22 (**10a**) and 4.27 (**10b**) (indicated by the authors as “Sosbo”, acronym for “sirtuin one selective benzoxazines”), emerged as the best inhibitors of the series, with IC_50_ values against SIRT1 of 0.15 and 0.22 μM, respectively, and possessed the same uncompetitive inhibition mechanism as **9a**. In addition, docking studies highlighted that the bicyclic ring of **10a,b** fits perfectly into the SIRT1 binding pocket with great overlap with **9b**. Similar to **9a**, **10b** has a stereogenic center with the *S*‐enantiomer [***S*** **−10b**, IC_50_ (SIRT1) = 0.11 μM] being 400x more potent than the *R*‐enantiomer. IC_50_ values for SIRT2 were 10.6 µM (**10a**), 37.7 µM (**10b**), and 34.0 µM (***S‐*** **10b**), while IC_50_ values for SIRT3 were over 60 µM for all compounds. Both **10a** and **10b** are cell permeable and enhanced acetylation of p53 at Lys382 in TNBC cells MDA‐MB‐231. No further experiments to confirm target engagement were performed.^311^

Inauhzin (**11**) is a SIRT1i bearing both a triazino[5,6‐*b*]‐indole and a phenothiazine moiety (Figure 6).^312^ **11** inhibits SIRT1 with an IC_50_ value between 0.7 and 2 µM, while it does not affect SIRT2,3 and HDAC8 activities. Compound **11** was shown to activate p53 and mediate p53‐dependent cytotoxicity in human lung carcinoma H460 cells. Additionally, **11** increased Lys382 acetylation of p53 in a dose‐dependent manner, which aligns with the inhibition of SIRT1. Moreover, **11** also stabilized p53 and inhibited its MDM2‐mediated ubiquitylation. Consistently with the substantial lack of SIRT2 inhibition, **11** did not affect the acetylation levels of α‐tubulin. Moreover, it induced apoptosis and suppressed tumor growth of H460 xenografts harboring p53.^312^

AGK2 (**12a**, Figure 6) has been reported as a single digit micromolar SIRT2 inhibitor with IC_50_ values of 3.5^80^ or 8 μM,^313^ depending on the study. Inhibition toward other SIRT isoforms was also tested, with an IC_50_ for SIRT1 higher than 50 μM in one study^80^ and 42 μM in another.^313^ Nevertheless, **12a** was over SIRT3 and 6.^80^, ^313^ Compound **12a** raised α‐tubulin acetylation levels in HeLa (Western blot^80^ and immunofluorescence^314^) and breast cancer MCF‐7 cells (immunofluorescence),^313^ consistent with SIRT2 inhibition, however, no further target engagement experiments were performed. **12a** administration was shown to have a positive influence in cellular and animal models of PD by protecting dopaminergic neurons from α‐synuclein damage.^80^ Moreover, **12a** was shown to inhibit the replication of hepatitis B virus both in cells and in vivo,^315^, ^316^ while it did not show anticancer activity at cellular level.^313^

MC2494 (**12b**) is a **12a** derivative, bearing a 4‐(2‐chlorobenzoyl)‐pyrrole in place of the phenyl‐substituted furan (Figure 6), with pan‐SIRT inhibitory activity [IC_50_ (SIRT1) = 38.5 μM; IC_50_ (SIRT2) = 58.6 μM, ~45% SIRT3 inhibition at 50 μM, ~63% SIRT4 inhibition at 50 μM, ~85% SIRT5 inhibition at 50 μM, ~55% SIRT6 inhibition at 50 μM].^317^ In HEK293FT cells, **12b** was shown to augment the acetylation of RIP1 kinase at two sites and CETSA experiments (**12b** concentration of 50 μM) confirmed its target engagement with SIRT1‐3. **12b** also exhibited antiproliferative activity and stimulated apoptosis via the RIP1/caspase‐8 pathway in AML U937 and TNBC MDA‐MB‐231 cells. In U937 cells, **12b** was shown to modulate mitochondrial functions by impairing ATP synthesis, oxidative stress response, and energy metabolism, thereby interfering with cancer cell homeostasis, and blocking tumorigenesis.^318^ Moreover, **12b** displayed promising anticancer activity in leukemic blasts, xenograft and allograft mouse cancer models, and reduced chemically‐induced proliferation of mammary gland in vivo.^317^ Compounds **12c**,**d** are **12b** derivatives obtained by replacing the α‐cyano moiety with a carbethoxy group.^319^ In **12c** the chloride atom at *ortho* position of the benzoyl portion is kept, while it is replaced by a methyl moiety in **12d** (Figure 6). These modifications increased SIRT2 inhibition compared with **12b** (74% and 79% at 50 μM, respectively), while decreasing the activity toward SIRT1 (<25% and <50% inhibition at 50 μM, respectively). This data is in line with the significant rise of α‐tubulin acetylation in U937 AML cells, along with the lack of influence on the acetylation levels of H3K9 and H3K14, two known substrates of SIRT1. Nevertheless, different from the parent molecule **12b**, compounds **12c** and **12d** were not validated via CETSA. Overall, the data indicate that insertion of a carbethoxy group in place of the cyano moiety switches compound activity toward SIRT2 inhibition.^319^

In 2001, following a high‐throughput phenotypic screening on a library of 1600 compounds, Grozinger et al. identified the β‐naphthol derivative Sirtinol (**13a**, Figure 6) as a micromolar SIRT1/2 inhibitor,^320^ with a slight preference for SIRT2, according to the numerous IC_50_s reported in literature [IC_50_(SIRT1) = 37–131 µM; IC_50_(SIRT2) = 38–58 µM].^321^ Compound **13a** was reported to increase p53 acetylation and exert antiproliferative, proapoptotic, and autophagy‐inducing effects in different cell lines.^322^, ^323^, ^324^ However, while no further experiments investigating its cellular target engagement are available, there are studies suggesting a pleiotropic activity for this molecule, whose effects may be due to the modulation of other targets beyond SIRTs. For instance, **13a** was indicated to modulate androgen, estrogen and insulin‐like growth factor‐1 pathways.^325^ In addition, **13a** behaves as an iron chelator both in vitro and in leukemia cell lines, and this action might contribute to its several biological effects.^326^

Another β‐naphthol containing inhibitor is Salermide (**13b**), a **13a** analog possessing a reverse amide structure at the *meta* position of the central benzene ring (Figure 6). **13b** inhibits SIRT1 and SIRT2 with IC_50_ values of 42.8 and 25.0 μM, respectively. The same compound was shown to promote tumor‐specific cell death in a wide range of human cancer cell lines.^327^, ^328^ In cancer cells, **13b** induced p53‐driven apoptosis by reactivating the expression of proapoptotic factors, while it does not affect healthy cells. Indeed, when exposed to 25 μM of **13b**, acute lymphoblastic leukemia (ALL) cells MOLT‐4 were found to overexpress the same subset of genes that were upregulated following SIRT1 silencing by RNA interference. However, no target engagement assays were performed. Finally, **13b** shows antiproliferative activity in glioblastoma multiforme (GBM) and CRC CSCs.^327^, ^329^ **13b** was also tested in *Caenorhabditis elegans* for its ability to revert the toxicity of the mutant polyadenylate‐binding protein nuclear 1 (PABPN1), whose activity is regulated by SIRT1. Mutant PABPN1 causes polyalanine expansion, leading to nuclear collapse and motility defects. In humans, this leads to oculopharyngeal muscular dystrophy. Under these conditions, **13b** was able to rescue *C. elegans* nuclear collapse and restore nematode mobility, in a similar manner to what was observed for SIRT1‐selective inhibitor ***S*** **−9a**, which was tested in the same assays.^328^

Cambinol (**13c**) is another β‐naphthol containing compound (Figure 6) inhibiting both SIRT1 and SIRT2 [IC_50_(SIRT1) = 56 µM, IC_50_(SIRT2) = 59 μM] which displayed weak inhibitory activity toward SIRT5 and substantially no activity versus SIRT3.^330^ **13c** was shown to increase the acetylation of SIRT1 (p53) and SIRT2 substrates (α‐tubulin), but no other target engagement assays were performed. **13c** was also indicated to increase the sensitivity to DNA damaging agents via SIRT1 inhibition and consequent p53 activation. Notably, **13c** decreased tumor growth in a Burkitt lymphoma mouse xenograft model.^330^ Compound **13d**, the *n*‐butyl substituted derivative of **13c**, is a low‐micromolar SIRT2 inhibitor selective over SIRT1 [IC_50_(SIRT1) = 16.9 µM, IC_50_(SIRT2) = 1.0 µM] capable of increasing the levels of acetyl α‐tubulin in NSCLC cell line H1299.^331^ Docking analysis revealed that the β‐naphthol moiety of **13c** is sandwiched between F119 and H187 in the C‐pocket of the catalytic domain, forming π‐stacking interactions that are crucial for a stable binding.^332^ The improvement in terms of activity and selectivity gained with **13d** compared with **13c** comes from the extra hydrophobic contacts between the *n*‐butyl chain and a previously vacant hydrophobic channel in the SIRT2 active site. In particular, the aliphatic carbon chain of **13c** was suggested to fit into a tight lipophilic channel defined by F96, L138, and I169.^331^ NMR‐directed experiments led to the discovery of a new class of isoform‐selective derivatives of **13c**.^333^ The insertion of a pyrazolone ring in place of the 2‐thiouracil of **13c** (Figure 6) increased the selectivity of the molecule, with a general preference for SIRT1, as indicated by compounds **13e** and **13f** exhibiting IC_50_ values against SIRT1 of 26 and 37 µM, respectively, while their activity versus SIRT2,3 was negligible. Notably, the insertion of a phenyl group at the 6‐position of the β‐naphthol ring shifts the selectivity toward SIRT3, as demonstrated by compound **13g** that displays an IC_50_ value for SIRT3 of 6 µM, with 7‐ and 5‐fold selectivity over SIRT1 and 2, respectively. Interestingly, the substitution of the pyrazolone ring with an isoxazol‐5‐one (**13h**) raises the selectivity toward SIRT2 with an IC_50_ value of 13 µM, while IC_50_ values for SIRT1,3 are higher than 200 µM. According to this study, the determining factor for the selectivity profile of this compound series is the presence of either a hydrogen bond donating (such as the pyrazolone NH in **13e**) or accepting (such as the isoxazol‐5‐one oxygen in **13h**) group. Among these four compounds, only **13f** and **13h** were tested for their target‐specific activity in cells and they were shown to dose‐dependently increase the levels of acetylated p53 and α‐tubulin, respectively. These results are in line with the inhibition of SIRT1 by **13f** and SIRT2 by **13h**.^333^ The evaluation of **13e**, **13g**, and **13h** against a panel of tumor cell lines (lymphoma, NSCLC, colon, and breast cancer) suggests SIRT2 as the preferred target for cancer treatment, since the selective SIRT2i **13h** is the most potent in terms of cytotoxicity (IC_50_s = 3–7 µM).^333^ The most recent development of 13a derivatives is represented by its open‐chain analogs such as compounds **13i,j** (Figure 6), that show submicromolar SIRT2 inhibition (IC_50_ value of 0.78 and 0.25 μM, respectively) along with selectivity over SIRT1 and 3.^334^ Compounds **13i,j** have the same 2‐hydroxynaphthyl moiety as **13a**. In this series, the bromine at position C6 of the naphthyl moiety was shown to be crucial for SIRT2 inhibition. The phenyl ring also present in **13a** was kept and decorated with 4‐Br or 4‐CF_3_, since SAR studies revealed that 4‐substitution is favored. **13i,j** were shown to induce apoptosis and exert antiproliferative effects in B‐cell lymphoma cell lines such as the Burkitt lymphoma lines Daudi (IC_50_ values of 7.1 and 11.9 μM, respectively) and Raji (IC_50_ values of 9.1 and 11.9 μM, respectively), and the DLBCL cells OCI (IC_50_ values of 5.7 and 24.9 µM, respectively). **13j** was also demonstrated to increase α‐tubulin acetylation at 5 and 10 μM in the NSCLC cell line NCI‐H460 after 18 h treatment, but no other target engagement studies were performed.^334^

Recently, Kang et al. developed the 2,3‐dihydroquinazolin‐4(1*H*)‐one derivative MHY2245 (**13k**, Figure 6) obtained via a cyclization and molecular simplification strategy applied on the structure of Sirtinol (**13a**).^335^ They also reported the analog MHY2251 (**13l**), characterized by the presence of a benzo[*d*][1,3]dioxol‐5‐yl moiety in place of the naphthalen‐1‐yl group (Figure 6).^336^ Compound **13k** inhibited SIRT1 deacetylation by ~50% at 1 µM, while **13l** could reach 40% inhibition only at 10 µM. Nevertheless, the selectivity over other isoforms was not evaluated for either compound. Interestingly, both **13k** (0.25–1 µM) and **13l** (2.5–10 µM) decreased SIRT1 and SIRT2 expression in CRC HCT116 cells, while they increased the levels of phospho‐H2AX and p53. Moreover, both **13k** and **13l** decreased the viability of multiple CRC cell lines, with the greatest influence on HCT116 cells (50% inhibition after 48 h at 1 µM for **13k** and 10 µM for **13l**). Both molecules were also shown to induce apoptosis in HCT116 cells. Nevertheless, given the absence of selectivity and target engagement assays it is hard to set a causal link between SIRT1 inhibition and the observed effects.^335^

In 2008, Lain et al. reported the hit compound Tenovin‐1 (**14a**), initially identified as a p53 activator in various tumor cell lines. Further optimization to increase its water solubility led to Tenovin‐6 (**14b**, Figure 6) showing improved p53 activation.^71^ Both compounds were shown to inhibit SIRTs, but **14a** was not sufficiently water soluble to allow titration experiments, while the IC_50_ values of **14b** were 21, 10, and 67 µM for SIRT1, 2, and 3, respectively. Another study tested **14b** against SIRT1,2,3, and 6 yielding IC_50_ values of 26, 9, >50, and >200 μM, respectively, substantially confirming the results of Lain and colleagues. Compound **14b** raised p53 acetylation levels in MCF7 and H1299 cells,^71^, ^313^ and increased α‐tubulin acetylation in MCF7 cells,^313^ consistent with SIRT1 and SIRT2 inhibition, respectively. **14b** also induced apoptosis in several gastric and leukemia cancer cell lines and displayed cytotoxic effects on ARN8 melanoma cells, slowing the growth of ARN8‐derived xenograft tumors.^71^ Moreover, **14b** inhibited the cell growth of lymphoma, breast, colon, lung, cervical, and pancreatic cancer cells with GI_50_ values lower than 10 μM, although it also exhibited cytotoxicity against normal mammary epithelial cells.^313^ Finally, **14b** was indicated to impair autophagy independently from SIRT1 inhibition.^337^ In light of the lack of additional research on target engagement and the observation that **14b** produces effects independent of SIRT inhibition, it is challenging to establish a causal relationship between SIRT inhibition and the biological effects that have been observed so far.

**Figure 7:**
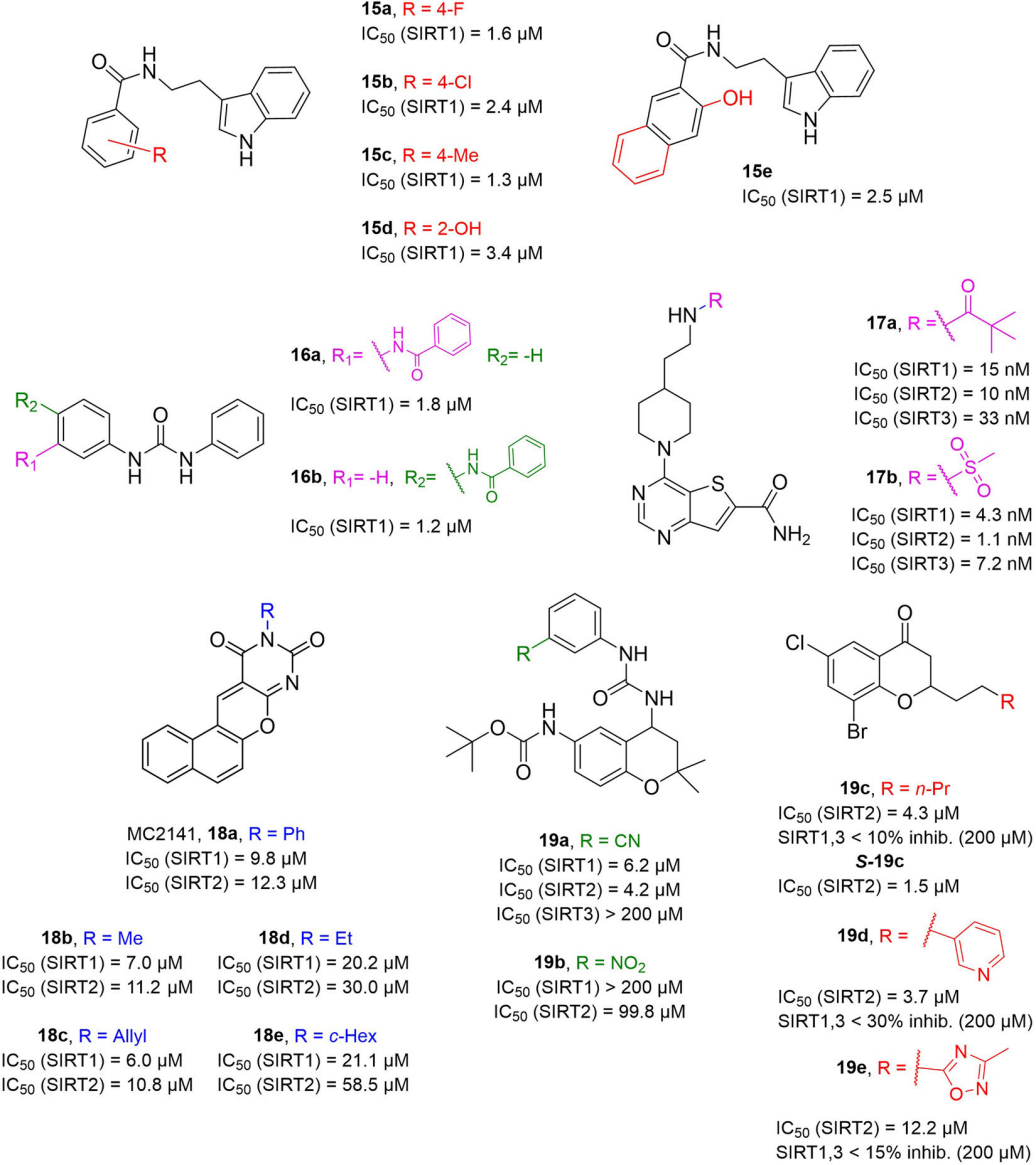
Structures and enzymatic activities of SIRTi **15a‐e**, **16a,b**, **17a,b**, **18a‐e**, and **19a‐e**. [Color figure can be viewed at wileyonlinelibrary.com]

An artificial intelligence (AI)‐driven virtual screening has recently allowed the identification of novel SIRT1 inhibitors. Starting from a library of 2.6 million compounds, Gryniukova and colleagues selected 434 molecules and finally managed to obtain 8 inhibitors with an IC_50_ value ≤ 10 µM.^338^ Among them, the most potent ones were the tryptamine derivatives **15a‐e** with IC_50_ values of 1.2–3.4 µM and the 1,3‐diphenylurea derivatives **16a, b** with IC_50_ values of 1.8 and 1.6 µM, respectively (Figure 7). Molecular dynamics (MD) identified three types of key interactions: the C‐H···F polarized bond between SIRT1 S265, I270 and the 4‐fluorobenzamide moiety of **15a**; hydrogen bonds between R274 and the benzamide oxygen and between V412 backbone carbonyl and the indole NH; hydrophobic interactions formed by F273 and the 4‐fluorobenzamide as well as F297/H363 and the indole core.^338^ Further studies will be necessary to assess the specificity and cellular activity of these compounds.

An interesting class of potent SIRT1‐3 inhibitors is represented by compounds **17a,b**, identified through a DNA‐encoded library screen initially aimed at finding SIRT3 selective inhibitors (Figure 7).^294^ Both compounds display an inhibitory potency in the nanomolar range, with **17a** (IC_50_s of 15, 10, and 33 nM for SIRT1,2,3 respectively), having a pivalamide on the sidechain, being slightly less potent than **17b** (IC_50_s of 4.3, 1.1, and 7.2 nM for SIRT1,2,3 respectively), bearing a methanesulfonamide in the same position. According to SIRT3‐**17a** and SIRT3‐**17b** co‐crystal structures, both compounds interact with the active site pocket between the Zn^2+^ binding domain and the Rossmann fold, with the primary carboxamide binding to the C‐pocket and the aliphatic chain interacting with the substrate binding site. However, no assays were performed in the study to investigate the cellular effects of these compounds.^294^

MC2141 (**18a**) is a benzodeazaoxaflavin derivative inhibiting both SIRT1 (IC_50_ = 9.8 µM) and SIRT2 (IC_50_ = 12.3 µM). Substitution of the *N‐*phenyl portion with allyl [**18b**, IC_50_ (SIRT1) = 6.6 µM, IC_50_ (SIRT2) = 10.8 µM] or methyl [**18c**, IC_50_ (SIRT1) = 7.0 µM, IC_50_ (SIRT2) = 11.2 µM] groups increases the activity toward both enzymes, while ethyl (**18d**) or cyclohexyl (**18e**) groups determine a drop of the inhibitory potency [**18d**: IC_50_ (SIRT1) = 20.2 µM, IC_50_ (SIRT2) = 30.0 µM; **18e**: IC_50_ (SIRT1) = 21.1 µM; IC_50_ (SIRT2) = 58.5 µM]. Notably, when tested at 50 μM in MCF7 cells, compound **18a** was shown to increase p53 acetylation, but not α‐tubulin acetylation, thus suggesting inhibition of SIRT1, but not SIRT2 in cells. No other target engagement assays were performed for **18a** or its derivatives **18b‐e**. All these compounds displayed proapoptotic properties in AML U937 cell lines, with **18a** and **18b** exhibiting higher antiproliferative effects in CRC and GBM CSCs (IC_50_s = 4–10 µM) than **18b** and **18d**. This finding may be attributed to increased cellular permeability caused by the presence of an unsaturated moiety on N10.^339^, ^340^, ^341^

In 2017, Schnekenburger and colleagues tested 1‐(2,2‐dimethylchroman‐4‐yl)−3‐phenylurea derivatives on a panel of different glioblastoma cell lines.^342^ Among the compounds, **19a** and **19b** were the best‐performing ones, indicating that electron‐withdrawing groups positioned in *meta* of the phenyl ureido moiety are preferential for compound activity, with an average GI_50_ value of 8 µM in both cases (Figure 7). The main difference between the two molecules was the selectivity index (ratio between GI_50_ of normal and tumor cells), which was >10 for **19a** and <5 for **19b**. Enzymatic assessment indicated that **19a** is a micromolar SIRT1/2 inhibitor [IC_50_ (SIRT1) = 6.2 µM, IC_50_ (SIRT2) = 4.2 µM, IC_50_ (SIRT3) > 200 µM], and this result was supported by docking studies. In contrast, **19b** exerted only weak SIRT2 inhibition [IC_50_ (SIRT1) > 200 µM, IC_50_ (SIRT2) = 99.8 µM, IC_50_ (SIRT3) not measured], implying that the observed cellular effects are likely the consequence of interactions with other targets. Compound **19a** was also selective over HDAC1‐3, 6, 8, 10, 11 at 100 μM. Moreover, **19a** elicited dose‐dependent increase of α‐tubulin acetylation in the glioblastoma cell line U373, but not in Hs683, even at the highest‐tested concentration of 10 μM. This may be due to a different expression level of SIRT2 in the two cell lines. Regarding SIRT1, **19a** could increase the acetylation levels of its substrates H4 and H3K56 in both cell lines. However, the acetylation of p53 was not examined, and no further tests were conducted to fully assess target engagement.^342^

The 4‐chromanone compound **19c** (Figure 7), synthesized by Friden‐Saxine et al., showed low‐micromolar SIRT2 inhibition (IC_50_ = 4.3 µM as racemate, IC_50_ = 1.5 µM for the *S*‐enantiomer ***S*** **−19c**) with selectivity over SIRT1 and 3. Although no cell‐based assays were performed, this study indicated that electron‐withdrawing groups at C6 and C8 are highly beneficial for compound activity, while the carbonyl portion is essential.^343^ Compound **19c** has been the starting point for the development of novel SIRT2 selective inhibitors such as **19d** and **19e** (Figure 7).^344^ These two molecules, bearing pyridine and oxadiazole moieties connected with an ethyl linker to the chroman‐4‐one core, showed IC_50_ values against SIRT2 (as racemic mixtures) of 3.7 and 12.2 μM, respectively. The molecules kept their selectivity over SIRT1 and SIRT3 and did not inhibit other HDAC isoforms. To evaluate their anticancer potential, **19d** and **19e** were tested in lung (A549) and breast (MCF7) cancer cell lines, where they showed antiproliferative activity. Furthermore, both **19d** and **19e** were able to induce dose‐dependent hyperacetylation of α‐tubulin in the MCF7 cell line, while no other target engagement assays were performed.^344^ According to docking studies, these molecules possess a binding mode similar to the one reported for other SIRTi which occupy the nicotinamide binding site in the C‐pocket, preventing the binding of NAD^+^ in a catalytically active conformation.^344^

**Figure 8:**
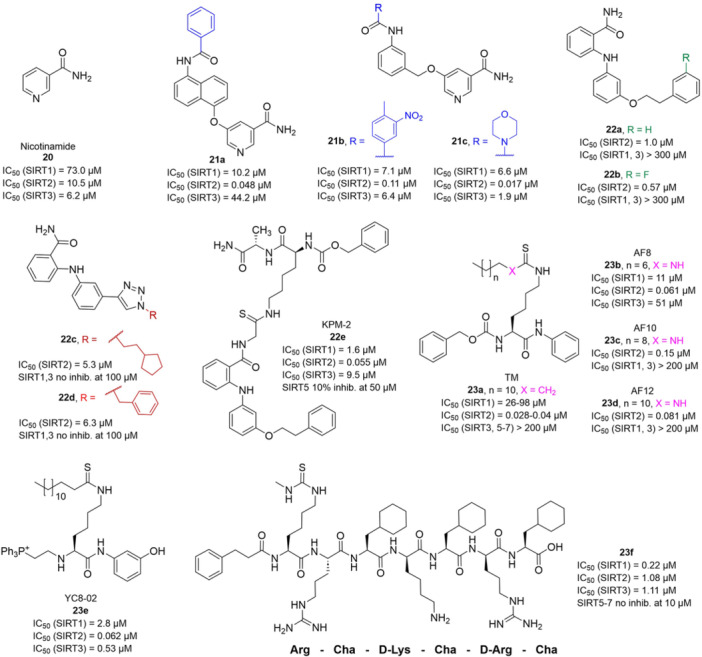
Structures and enzymatic activities of nicotinamide (**20**), its SIRT2‐selective derivatives **21a‐c** and **22a‐e**, the thiomyristoyl lysine compound TM (**23a**), and **23a**‐based compounds **23b‐f**. [Color figure can be viewed at wileyonlinelibrary.com]

Nicotinamide (**20**, Figure 8) is a sirtuin deacylation product and acts as an endogenous inhibitor of all SIRT isoforms, with IC_50_ values spanning in the mid micromolar range.^345^, ^346^, ^347^, ^348^ Its inhibitory mechanism consists of rebinding to the C‐pocket after the release, with a successive nucleophilic attack of the pyridine nitrogen to the *O*‐alkylimidate intermediate. Many biological effects have been reported for **20**, including its ability to induce α‐tubulin hyperacetylation and restore cognitive deficits in an AD mouse model.^349^ From a medicinal chemistry point of view **20** is important mainly as progenitor of various series of SIRTi.^350^

In 2014 Cui et al. developed a series of (5‐benzamidonaphtalen‐1/2‐yloxy)nicotinamide analogs (Figure 8), among which **21a** was the most potent derivative, that selectively inhibited SIRT2 over SIRT1 and SIRT3, with IC_50_ values of 0.048, 10.2, and 44.2 µM, respectively.^351^ Kinetic studies revealed that **21a** is a competitive inhibitor of the peptide substrate and a noncompetitive inhibitor toward NAD^+^.^351^ **21a** raised α‐tubulin acetylation levels in MCF7 breast cancer cells and displayed mild cytotoxic activity against MCF7, prostate (DU145), and CML (K562) cancer cells, with CC_50_ values of 30.6, 33.3, and 26.2 µM, respectively.^351^ The structural optimization of **21a** led to compounds **21b** and **21c** (Figure 8), which selectively inhibited SIRT2 over SIRT1 and 3 acting as competitive inhibitors of both peptide substrate and NAD^+^. In fact, compound **21b** displayed an IC_50_ for SIRT2 of 0.11 µM, with ∼60‐fold selectivity over SIRT1/3, while compound **21c**, bearing a characteristic morpholino ureido function, exhibited nanomolar activity against SIRT2 (IC_50_ = 0.017 µM) along with >390, and 100‐fold selectivity over SIRT1 and 3, respectively. **21c** is endowed with good metabolic stability and blood‐brain barrier permeability and was protective toward α‐synuclein induced cytotoxicity in SH‐SY5Y neuroblastoma cells, thereby representing an interesting starting point for PD drug development. Nevertheless, no target engagement studies were performed for **21b** or **21c**.^349^

Compounds **22a** and **22b** (Figure 8) are (sub)micromolar inhibitors of SIRT2 [IC_50_(**22a**) = 1.0 µM, IC_50_(**22b**) = 0.57 µM] selective over SIRT1 and 3,^352^ with **22a** also being selective over other HDAC isoforms and displaying only weak inhibition toward CYP450 isoforms (e.g., 3A4 or 2D6, IC_50_ values > 10 μM). According to SAR analysis, the phenyl ring at the end of the phenethoxy tail is essential for selectivity, while the ethyl chain is necessary for potent inhibition. When tested in colon cancer cells, **22a** induced an increase in α‐tubulin acetylation levels in a dose‐dependent manner. No other cellular assays were performed for **22a**, while **22b** was not assessed for its cellular activity at all.^352^ Following this study, the same group reported further 2‐anilidobenzamides such as A1B11 (**22c**) and A2B57 (**22d**), possessing a 1,2,3‐triazole ring that replaces the phenethoxy moiety on the 3‐position of the central aniline.^353^ These molecules, obtained through a click chemistry approach, showed IC_50_ values against SIRT2 of 5.3 µM (**22c**) and 6.3 µM (**22d**) and had no effects on SIRT1/3 at 100 µM. However, they were only assessed in purified protein‐based biochemical assays.^353^ A recent study reporting the structure of the complex between **22a** and SIRT2 indicates that **22a** binds to a hydrophobic pocket between a small region near the C‐pocket and the Rossmann‐fold domain.^354^ The 2‐aminobenzamide portion of **22a** points toward the acetyl‐lysine tunnel interacting with conserved water molecules via hydrogen bonds while the phenethoxyphenyl moiety interacts with F131, L134, L138, Y139, P140, F143, and I169 through H–π and π–π interactions. Based on this data, Mellini et al. developed the pseudopeptide KPM‐2 (**22e**, Figure 8) as a mechanism‐based inhibitor of SIRT2 (IC_50_ = 0.055 µM), selective over SIRT1 and 3.^354^ In **22e**, the primary amide of **22a** is linked to a thioacetyllysine pseudopeptide, which was hypothesized to bind to the SIRT2 substrate pocket. This approach is not novel since it has been exploited by other groups in SIRTi drug design over the past 15 years.^355^, ^356^ Mass spectrometry (MS) experiments showed that the thioacetamide group is involved in a nucleophilic attack on NAD^+^, yielding a covalent intermediate with ADP and ribose which affords an in‐situ occupation of both NAD^+^ and substrate pockets. In MDA‐MB‐231 and MCF7 breast cancer cells, **22e** increased α‐tubulin acetylation and displayed antiproliferative activity in the low micromolar range. Notably, in N2a neuroblastoma cells **22e** increased the number of differentiated cells versus control and exhibited neurite outgrowth potential.^354^

The thiomyristoyllysine‐based compound TM (**23a**, Figure 8) is a potent SIRT2i (IC_50_ values of 0.028^357^ or 0.04 µM,^313^ depending on the study) displaying selectivity over SIRT1, 3, and 5‐7.^313^, ^357^ Kinetic and MS‐based experiments indicated that **23a** is a mechanism‐based inhibitor able to compete with the substrate, but not the NAD^+^ co‐substrate. Western blot and immunofluorescence experiments indicated that **23a** dose‐dependently increases α‐tubulin acetylation in breast cancer and CRC cell lines, while it did not affect p53 acetylation.^357^, ^358^ In breast cancer cell lines, and specifically in *c*‐Myc‐driven cancers, **23a** displays potent antiproliferative activity and its effects were similar to SIRT2 knockdown. This is achieved via SIRT2 inhibition, which triggers ubiquitination and consequent degradation of *c*‐Myc. Furthermore, **23a** inhibits tumor growth in immunocompromised mouse models of breast cancer.^357^ Nonetheless, **23a** suffers from low solubility and difficult preparation, which prompted the group to develop new inhibitors possessing improved solubility and an easier synthesis. These compounds possess a thiourea group and shorter alkyl tails and retain SIRT2 inhibitory activity. Specifically, AF8 (**23b**), AF10 (**23c**), and AF12 (**23d**) possess IC_50_ values against SIRT2 in the submicromolar range (0.061, 0.15, and 0.081 µM, respectively) and are selective over SIRT1 (IC_50_ = 11 µM for **23b** and IC_50_s > 200 µM for **23c**,**d**) and SIRT3 (IC_50_ = 51 µM for **23b** and IC_50_s > 200 µM for **23c**,**d**) (Figure 8). Moreover, **23b,c** increased α‐tubulin acetylation in the HCT116 CRC cell line, while **23d** was not assayed. Finally, they displayed anticancer activity in CRC cells, with **23b** being able to reduce tumor growth in a mouse xenograft CRC model.^358^

Another peptide‐based SIRT inhibitor is YC8‐02 (**23e**, Figure 8), A derivative of **23a** bearing a triphenylphosphonium (TPP) moiety for mitochondrial targeting, designed to preferentially inhibit SIRT3 in cells.^121^ Biochemical assays indicated that **23e** inhibits SIRT1, 2, and 3 with IC_50_ values of 2.8, 0.062, and 0.53 µM, respectively, thus being 8.5‐fold more potent against SIRT2 than SIRT3. Selectivity toward other SIRT isoforms was not assayed. YC8‐02 was shown to completely abolish the cell viability of different DLBCL cell lines at 10 µM and to increase mitochondrial protein acetylation, but no other target engagement studies were performed. Moreover, YC8‐02 suppressed lymphoma growth in DLBCL mouse xenograft. Despite the presence of a mitochondria‐targeting moiety, we cannot rule out the possibility that the observed effects are attributable to SIRT2, considering the high in vitro selectivity of YC8‐02 for SIRT2 over SIRT3.^121^

A similar approach was employed by Troelsen and colleagues, who linked a mitochondria‐targeting peptide, characterized by an alternation of cationic and lipophilic amino acids,^359^ to a modified TM residue, yielding the peptide‐based compound **23f** (Figure 8).^360^ In biochemical assays, compound **23f** inhibits SIRT1, 2, and 3 with IC_50_ values of 0.22, 1.08, and 1.11 µM, thereby being fivefold more potent against SIRT1. Moreover, it was shown to be selective over HDAC1‐3 and SIRT5‐7 at 10 µM. CETSA performed in HEK293T cells indicated that **23f** can stabilize in cells both SIRT1 and SIRT3, but not SIRT2, thus suggesting that the presence of the mitochondrial‐targeting peptide does not fully overcome the higher potency toward SIRT1. However, treatment of HEK293T cells with compound **23f** increased the acetylation of the SIRT3 substrate SOD2 while having no effect on p53.^360^ Overall, further studies will be necessary to improve SIRT3 inhibitory potency to yield cellularly selective SIRT3 inhibitors derived from **23f**.

**Figure 9:**
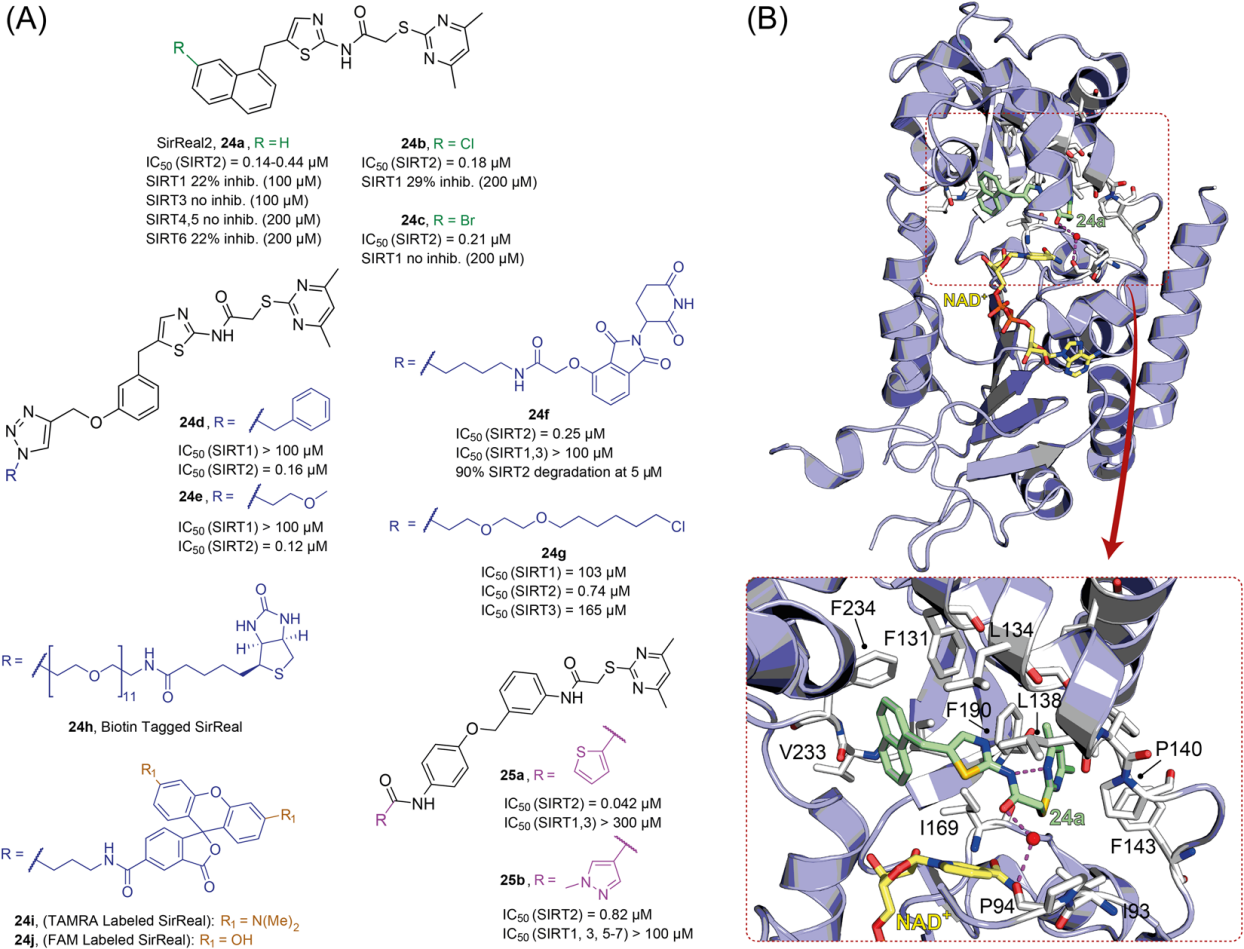
(A) Structures and enzymatic activities of SirReal2 (**24a**) and its most interesting analogs (**24b‐j**, **25a,b**). (B) hSIRT2/NAD^+^/**24a** co‐crystal structure (PDB ID: 4RMG) showing the key interaction between the small molecule (green sticks) and the enzyme (light blue cartoon, with key residues shown in white). NAD^+^ is depicted as yellow sticks; hydrogen bonds involving **24a**, P94, and a conserved water molecule (red sphere) are depicted as purple dotted lines. [Color figure can be viewed at wileyonlinelibrary.com]

SirReal2 (**24a**, Figure 9A) is a SIRT2i with IC_50_ values in the submicromolar range (0.14,^361^ 0.23,^313^ or 0.44 µM,^362^ depending on the study) and negligible effects on SIRT1, and 3‐6 (Table 2).^361^ The SIRT2‐**24a** co‐crystal structure indicates that, upon **24a** binding, SIRT2 undergoes a conformational change toward an open conformation of the catalytic site. **24a** has a rigid conformation in the SIRT2 binding pocket thanks to an intramolecular hydrogen bond between the amide group and one pyrimidine nitrogen. The **24a**‐SIRT2 binding is mostly driven by hydrophobic contacts. The naphtyl portion of **24a** forms hydrophobic interactions with the nicotinamide moiety of NAD^+^ and with residues placed at the entrance of the acyllysine binding site such as F131, L134, I169, I232, V233, and F234. **24a** also interacts with residues located in a previously unexplored site (called “selectivity pocket”) situated close to the Zn^2+^‐binding domain (Figure 9B).^361^ In the selectivity pocket, the dimethylpyrimidine group forms π‐stacking interactions with Y139 and F190 and a conserved water molecule acts as a bridge via hydrogen bonding with P94 and the **24a** carbonyl oxygen. **24a** increased α‐tubulin and microtubule network acetylation in HeLa cells with no cell cycle alterations. A later study indicated that **24a** increases α‐tubulin acetylation in the breast cancer cell line MCF7.^313^ Moreover, another investigation employed CETSA in HEK293T cells and showed that **24a** stabilizes SIRT2, but not SIRT1 and SIRT3 in cells, thus confirming the target engagement.^360^ Compound **24a** was shown to impair the growth of lymphoma, breast, colon, lung, and cervical cancer with GI_50_ values between 11 and 30 μM, while it had no effects on pancreatic cancer cells. Nevertheless, **24a** was also cytotoxic for normal mammary epithelial MCF‐10A cells (GI_50_ = 11 μM).^313^ Substitution of naphthalene C7 position with Cl or Br yielded **24b** [IC_50_(SIRT2) = 0.18 µM] and **24c** [IC_50_(SIRT2) = 0.21 µM], respectively (Figure 9A), which were selective over SIRT1, while no activity was assessed against other SIRT isoforms. Notably, inspection of the structure of the complex between **24c** and SIRT2 indicates a binding mode mainly sustained by hydrophobic contacts like **24a**. Moreover, **24b** increased α‐tubulin acetylation in HeLa cells at 10 μM, while **24a** only at 20 μM, and **24c** was not evaluated in this assay. Nonetheless, the halogenated compounds **24b,c** are poorly soluble in water, thus they were only tested at low concentrations.^362^ To increase the potency and selectivity of **24a**‐based inhibitors, the arylalkyl moiety of **24a** was extended to occupy the SIRT2 acylated lysine binding site. This led to triazole‐based derivatives **24d** and **24e** (Figure 9A), endowed with improved SIRT2 inhibition [IC_50_(**24d**) = 0.16 µM, IC_50_(**24e**) = 0.12 µM], along with SIRT1,3 selectivity. SIRT2/**24d** co‐crystal structure indicates a similar binding mode to **24a** and shows that the triazole moiety points toward the acylated lysine binding site and forms multiple hydrogen bonds with R97 in the cofactor binding loop. Both molecules also displayed higher aqueous solubility and induction of α‐tubulin acetylation compared with the parent compound **24a** and AGK‐2 (**12a**).^314^ The triazole derivatives were further exploited for the development of various tools used to explore SIRT2 biological roles (**24f‐j**). Among them, **24f** consists of a SirReal scaffold coupled through a proper triazole‐containing linker with thalidomide, a ligand of the E3 ubiquitin‐ligase Cereblon (Figure 9A).^363^ This represents the first example of a proteolysis targeting chimera (PROTAC) targeting a SIRT family member.^364^ Indeed, in addition to inhibiting SIRT2, **24f** can induce its selective degradation at low micromolar concentrations (90% of SIRT2 degradation at 5 µM) and promote hyperacetylation of α‐tubulin in HeLa cells at the same doses.^363^ The chloroalkylated SirReal derivative **24g** (Figure 9A) is another SIRT2i [IC_50_(SIRT1) = 103 µM; IC_50_(SIRT2) = 0.74 µM; IC_50_(SIRT3) = 165 µM] developed by the same group that induces the degradation of this enzyme through the binding to the HaloTag 7 (HT7)‐tagged E3 ubiquitin‐ligase Parkin. HT7 is an engineered bacterial dehalogenase that forms covalent bonds with chloroalkanes and it has been used as a protein tag, replacing the native substrate binding domain of the Parkin.^365^ As a result, the tag enabled the E3 ligase to be recruited in proximity of SIRT2 and promoted its ubiquitin/proteasome‐mediated degradation. Hence, this study provided evidence that another E3 ligase beyond Cereblon may be exploited for the development of SirReal‐based SIRT2 chemical degraders.^366^ The biotinylated derivative **24h** (Figure 9A)^314^, ^367^ was described as a valuable tool for SIRT2 pull‐down, chemoproteomic studies, and biolayer interferometry biophysical screenings. Indeed, **24h** was shown to specifically capture SIRT2 from the lysates of the AML cell line HL60. The fluorescent derivatives **24i** and **24j** (Figure 9A) were reported for fluorescence polarization (FP) assays and cellular target engagement analysis.^368^

**Table 2:** Most relevant SIRT1/2 inhibitors.

Compd.	Molecular structure	Enzymatic activity	Cell‐based/in vivo effects	References
***S*** **‐9a** (*S*)‐Selisistat	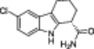	IC_50_ (SIRT1) = 0.038–0.69 µM IC_50_ (SIRT2) = 1.5–19.6 µM IC_50_ (SIRT3) = 48.7 µM	– Early‐stage HD patients: clinical, cognitive, and neuropsychiatric progress. – Cancer cells panel: higher acetyl‐p53 levels and enhancement of cytotoxic drug efficacy. – HepG2 and Huh7 HCC cells: lower survival and migration. – Chemotherapy‐resistant esophageal cancer cells: impairment of EMT and cell migration. – Ovarian carcinoma cells: reduction of colony development. – Pancreatic cancer PANC‐1 cells: double faced role → enhancement of gemcitabine cytotoxicity but also increased tumor growth in PANC‐1 mouse xenograft model.	[294, 295, 297, 301, 302, 303, 304, 305, 306, 307, 308, 309, 310]
**10a** 4.22	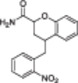	IC_50_ (SIRT1) = 0.15 µM IC_50_ (SIRT2) = 10.6 µM IC_50_ (SIRT3) > 60 µM	Human breast adenocarcinoma cells: increase of p53 acetylation levels.	[312]
**11** Inauhzin	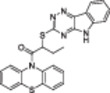	IC_50_ (SIRT1) = 0.7–2 µM IC_50_ (SIRT2, 3) > 50 µM	– Human lung carcinoma cells: p53 activation and higher p53‐dependent cytotoxicity; increased p53 acetylation at Lys382; inhibition of MDM2‐mediated p53 ubiquitination. – Lung carcinoma H460 mouse xenograft: apoptosis induction and tumor growth suppression.	[313]
**12a** AGK2	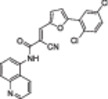	IC_50_ (SIRT2) = 3.5–8 µM IC_50_ (SIRT1) = 42–>50 µM IC_50_ (SIRT3) > 50 µM IC_50_ (SIRT6) > 100 µM	– HeLa cells: higher acetylation of α‐tubulin. – Breast cancer MCF7 cells: higher acetylation of α‐tubulin. – Cellular and animal PD models: opposes α‐synuclein toxicity in dopaminergic neurons.	[80, 314, 315]
**12b** MC2494	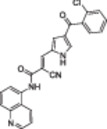	IC_50_ (SIRT1) = 38.5 µM IC_50_ (SIRT2) = 58.6 µM *Inhib. at 50 µM:* SIRT3 ~ 45% SIRT4 ~ 63% SIRT5 ~ 85% SIRT6 ~ 55%	– HEK293FT cells: higher RIP1 acetylation; target engagement with SIRT1‐3 (CETSA). – AML U937 and breast cancer MDA‐MB‐231 cells: increased RIP1/caspase‐8‐mediated apoptosis and decreased cell proliferation. – U937 cells: block of ATP synthesis, oxidative stress response, and energy metabolism. – *Ex vivo* leukemic blasts, allograft, and xenograft mouse cancer models: tumor growth impairment.	[318, 319]
**13b** Salermide	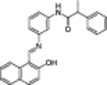	IC_50_ (SIRT1) = 42.8 µM IC_50_ (SIRT2) = 25.0 µM	– Cancer cells panel: tumor‐specific cell death; p53‐dependent apoptosis induction. – ALL cells: upregulation of genes similar to SIRT1 silencing. – GBM and CRC CSCs: antiproliferative activity.	[328, 330]
**13j**	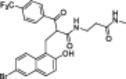	IC_50_ (SIRT2) = 0.25 µM SIRT1, 3 < 25% inhib. at 50 µM	– Burkitt lymphoma cell lines: apoptosis induction and antiproliferative activity [IC_50_ (Daudi) = IC50 (Raji) = 11.9 µM]. – OCI DLBCL cell line: apoptosis induction and antiproliferative activity (IC_50_ = 24.9 µM). – NSCLC NCI‐H460 cell line: increased α‐tubulin acetylation at 5 and 10 μM after 18 h treatment.	[335]
**17b**	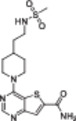	IC_50_ (SIRT1) = 4.3 nM IC_50_ (SIRT2) = 1.1 nM IC_50_ (SIRT3) = 7.2 nM	Not Available	[295]
**18a** MC2141	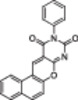	IC_50_ (SIRT1) = 9.8 µM IC_50_ (SIRT2) = 12.3 µM	– U937 AML cells: Apoptosis induction. – GBM and CRC CSCs: antiproliferative activity. – MCF7 breast cancer cells: increased p53 acetylation, but not α‐tubulin acetylation at 50 μM.	[340, 341, 342]
**19d**	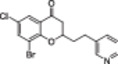	IC_50_ (SIRT2) = 3.7 µM SIRT1, 3 < 10% inhib. at 200 µM	– A549 Lung carcinoma and MCF7 breast cancer cells: antiproliferative activity and augmented α‐tubulin acetylation.	[345]
**21a**	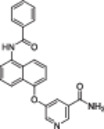	IC_50_ (SIRT1) = 10.2 µM IC_50_ (SIRT2) = 0.048 µM IC_50_ (SIRT3) = 44.2 µM	– MCF7 breast cancer cells: cytotoxicity (CC_50_ = 30.6 µM) and augmented α‐tubulin acetylation. – DU145 prostate cancer cells: cytotoxicity (CC_50_ = 33.3 µM). – K562 chronic myelogenous leukemia cells: cytotoxicity (CC_50_ = 26.2 µM).	[352]
**22e** KPM‐2	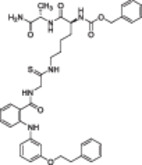	IC_50_ (SIRT1) = 1.6 µM IC_50_ (SIRT2) = 0.055 µM IC_50_ (SIRT3) = 9.5 µM SIRT5 10% inhib. at 50 µM	– MDA‐MB‐231 breast cancer cells: increased α‐tubulin acetylation and antiproliferative activity (GI_50_ = 8.3 µM). – MCF7 breast cancer cells: increased α‐tubulin acetylation and antiproliferative activity (GI_50_ = 6.2 µM). – N2a neuroblastoma cells: higher ratio of differentiated cells and neurite outgrowth activity.	[355]
**23a**	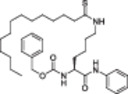	IC_50_ (SIRT1) = 26–98 µM IC_50_ (SIRT2) = 0.028–0.04 µM IC_50_ (SIRT3, 5‐7) > 200 µM	– Breast cancer cells, particularly *c‐*Myc‐driven cancers: induction of ubiquitination and degradation of *c‐*Myc via SIRT2 inhibition and antiproliferative activity. – Breast cancer and CRC cells: dose‐dependent increase of α‐tubulin acetylation with no effect on p53 acetylation. – Immunocompromised mouse models of breast cancer: impairment of tumor growth.	[314, 358]
**24a** SirReal2	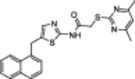	IC_50_ (SIRT2) = 0.14–0.44 µM *Inhib. at 100 µM:* SIRT1 ~ 22% SIRT3 no inhib *Inhib. at 200 µM:* SIRT4, 5 no inhib. SIRT6 ~ 22% inhib	– HeLa cells: increased α‐tubulin and microtubule network acetylation. – MCF7 breast cancer cells: increased α‐tubulin acetylation. – HEK293T cells: SIRT2, but not SIRT1 and 3 stabilization in CETSA. – Lymphoma, breast, colon, lung, and cervical cancer cells: growth inhibition with GI_50_ values between 11 and 30 μM, no effects on pancreatic cancer cells. – Normal mammary epithelial MCF‐10A cells: GI_50_ = 11 μM.	[314, 361, 362, 363]
**24 f**	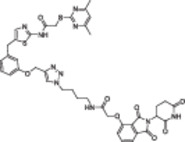	IC_50_ (SIRT2) = 0.25 µM 90% SIRT2 degradation at 5 μM SIRT1,3: no inhib. at 100 μM	– HeLa cells: selective SIRT2 degradation and higher of α‐tubulin acetylation.	[364]
**25a**	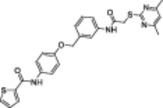	IC_50_ (SIRT2) = 0.042 µM IC_50_ (SIRT1, 3) > 300 µM	– MCF7 breast cancer cells: dose‐dependent reduction of viability of breast cancer cells and raise in α‐tubulin acetylation levels. – Human healthy liver HL‐7702 cells: no detected cytotoxicity.	[371]
**26** FLS‐359	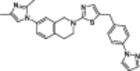	IC_50_ (SIRT2) = 3–7 µM IC_50_ (SIRT1,3) > 100 µM	– HCC HepG2 cells: increased levels of acetylated α‐tubulin. – TNBC MDA‐MB‐231 cells: decreased levels of c‐Myc. – RNA and DNA viruses (herpesviridae, coronaviridae, orthomyxoviridae, flaviviridae, and hepadnaviridae): replication inhibition with IC_50_ values between 0.3 μM (SARS‐CoV‐2) 6.7 μM (respiratory syncytial virus). – Humanized mouse models of human cytomegalovirus: antiviral action by inducing apoptosis and necroptosis of infected monocytes.	[373]
**27a** MC3465	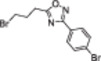	IC_50_ (SIRT2) = 1.5 µM SIRT1,3,5: no inhib. at 100 μM	– AML cell lines panel: α‐tubulin hyperacetylation, induction of apoptosis, antiproliferative activity [IC_50_ (Karpass299) = 25 µM].	[375]
**28** SR86	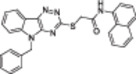	IC_50_ (SIRT2) = 1.3 µM IC_50_ (SIRT1, 3) > 300 µM	– MCF7 breast cancer cells: higher α‐tubulin acetylation and dose‐dependent antiproliferative activity.	[376]

Abbreviations: ALL, acute lymphoblastic leukemia; AML, acute myeloid leukemia; CETSA, cellular thermal shift assay; CRC, colorectal carcinoma; CSC, cancer stem cell; DLBCL, diffuse large B‐cell lymphoma; EMT, epithelial‐mesenchymal transition; GBM, glioblastoma multiforme; HCC, hepatocellular carcinoma; HD, Huntington's disease; NSCLC, non‐small cell lung cancer; PD, Parkinson's disease; TNBC, triple negative breast cancer.

Yang and colleagues performed a structure‐based study that resulted in the synthesis of many derivatives bearing the *N*‐aryl 2‐((4,6‐dimethylpyrimidin‐2‐yl)thio)acetamide moiety of **24a** after an in silico lead optimization campaign employing the in‐house developed tool LEADOPT^369^ and using the SIRT2/**24a** complex structure as model. The most potent identified compound was **25a** (Figure 9A), with an IC_50_ value for SIRT2 inhibition of 0.042 µM and great selectivity over SIRT1 and 3 (both IC_50_s > 300 µM). When tested in cells, **25a** reduced the viability of breast cancer MCF7 cells and augmented the acetylation of α‐tubulin in a dose‐dependent manner, while showing no cytotoxicity in human healthy liver HL‐7702 cells.^370^ In a subsequent study, through a structure‐based approach, the same group identified **25b**, a derivative of **25a** bearing a 1‐methyl‐1*H*‐pyrazole group in place of the thiophene moiety (Figure 9A).^371^ Although almost 20‐fold less potent than **25a** against SIRT2 [IC_50_(**25b**) = 0.82 µM], **25b** was highly selective against all available SIRT isoforms (SIRT1, 3, and 5‐7). The SIRT2/**25b** co‐crystal structure showed that **25b** causes an expansion of the hydrophobic pocket and resembles the binding of acyllysine substrates. When tested in NSCLC H441 cells, **25b** induced a dose‐dependent rise in α‐tubulin acetylation and stopped cell migration, invasion, and proliferation (IC_50_ = 3.93 µM).^371^ No further target engagement studies were performed for **25a** or **25b**.

**Figure 10:**
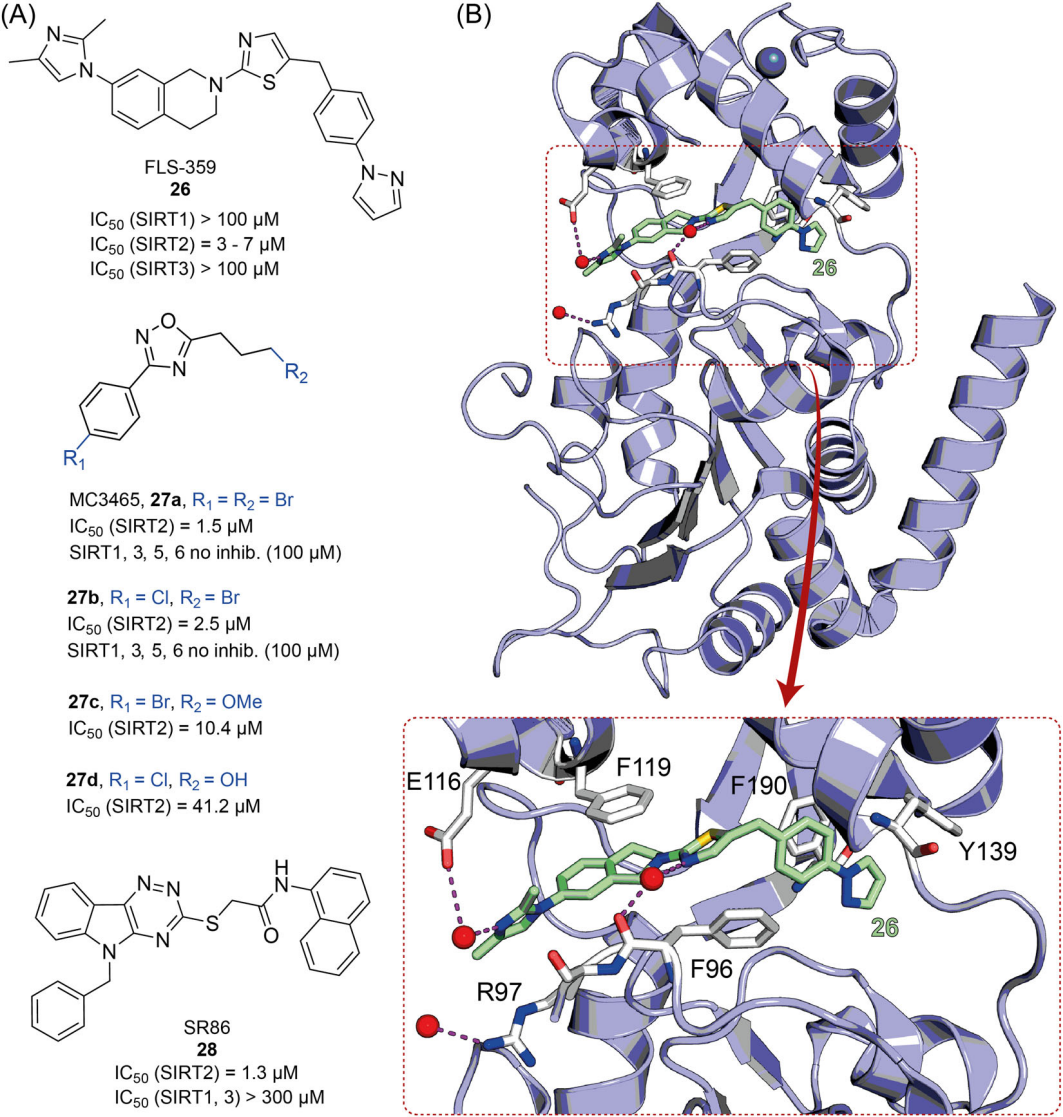
(A) Structures and enzymatic activities of SIRT2i **26**, **27a‐d** and **28**. (B) hSIRT2/**26** co‐crystal structure (PDB ID: 7T1D) showing the key interaction between the small molecule (green sticks) and the enzyme (light blue cartoon) The key interacting residues F96, R97, E116, F119, F190, and Y139 are shown as white sticks. Hydrogen bonds involving **26**, F96, E116, and conserved water molecules (red spheres) are depicted as purple dotted lines. [Color figure can be viewed at wileyonlinelibrary.com]

A recent screen aimed at finding new SIRT2 inhibitors led to FLS‐359 (**26**, Figure 10), presenting the same thiazolyl core as **24a**. FLS‐359 exhibited an IC_50_ value of 3 µM in a MS‐based SIRT2 deacetylation assay using a substrate peptide concentration of 5 µM, while not inhibiting SIRT2‐mediated demyristoylase activity and being selective over SIRT1 and SIRT3 (IC_50_ values > 100 µM).^372^ In an assay using 50 µM substrate peptide, the IC_50_ rose to 7 µM. Moreover, saturating compound concentrations did not abolish SIRT2 activity. Overall, these results suggest that **26** partially inhibits SIRT2. The co‐crystal structure of the SIRT2‐**26** complex (Figure 10B) shows the presence of three distinct sets of π‐π interactions, specifically between F119 and the thiophenyl core, F190 and the phenyl moiety, and Y139 and the pyrazole portion of **26**. Furthermore, a conserved water molecule acts as a bridge between the thiazole nitrogen and the backbone carbonyl of F96. Similarly, a network of water molecules mediates the interaction between the dimethyl imidazole of **26** and the side chains of E116 and R97. When tested in HCC HepG2 cells, **26** SIRT2 increased the levels of acetylated α‐tubulin. No further target engagement assays were performed. In TNBC MDA‐MB‐231 cells, **26** decreased the levels of *c*‐Myc, in line with the known effects of SIRT2 inhibition or knockdown.^372^ Notably, **26** exhibits inhibitory effects on the proliferation of RNA and DNA viruses, including *herpesviridae*, *coronaviridae*, *orthomyxoviridae*, *flaviviridae*, and *hepadnaviridae*. The IC_50_ values for the various viruses that were assessed vary in value, starting at 0.3 μM for SARS‐CoV‐2 and reaching 6.7 μM for respiratory syncytial virus. **26** also exhibited antiviral action in humanized mouse models of human cytomegalovirus and was later shown to act by inducing apoptosis and necroptosis in infected monocytes. The authors showed that SIRT2 inhibition impairs the deacetylation of Akt, required for the activation of the proapoptotic factor Mcl‐1.^373^

MC3465 (**27a**, Figure 10A) is an uncompetitive SIRT2i with an IC_50_ value of 1.5 µM, selective over SIRT1, 3, 5, and 6. By inhibiting SIRT2, **27a** increased α‐tubulin acetylation and induced apoptosis in various AML cell lines. Compound **27a** also displayed antiproliferative activity in the 25–100 µM range in a wide panel of AML cell lines, with preferential efficacy against Karpass299 (IC_50_ = 25 µM).^374^ Compound **27b**, which differed from **27a** only by having a chlorine at C4 of the phenyl ring instead of a bromine atom (Figure 10A), was slightly less potent [IC_50_ (SIRT2) = 2.5 µM] while remaining SIRT2 isoform‐selective. However, **27b** could not lead to apoptosis of the assessed cancer cell lines. Intriguingly, compound **27c**, bearing a methoxy moiety at phenyl C4 and showing an IC_50_ value of 10.4 µM, exhibited higher antiproliferative activity in the same cell lines, with IC_50_ values ranging from 10.3 to 62 µM and augmented α‐tubulin acetylation in AML cell lines NB4 and U937. The **27b** derivative bearing a hydroxyl group in place of the bromine at the end of the C5 side chain (**27d**, Figure 10A) was noted bound to SIRT2 during crystallization experiments aimed at yielding the SIRT2/**27b**/ADP‐ribose co‐crystal structure. This was probably due to the hydrolysis of the bromoalkyl tail triggered by the heating, freeze−thawing, and sonication treatments used during crystal soaking. The structure indicates that the **27b** interacts with the acyl‐lysine channel with the alkyl portion bound to the C‐pocket and the 4‐chlorophenyl ring placed in a small cavity within the hydrophobic pocket.^374^

Following a docking‐based virtual screening campaign, Huang et al. identified SR86 (**28**), a 5*H*‐[1,2,4]triazino[5,6‐*b*]indole derivative (Figure 10A) with high SIRT2 inhibition (IC_50_ = 1.3 µM) and no activity toward SIRT1 and 3.^375^ To elucidate the binding mode of **28**, the authors docked the molecule with SIRT2 and observed that the naphthalene of **28** fits perfectly in the selectivity pocket and the 5*H*‐[1,2,4]triazino[5,6‐*b*]indole moiety engages in hydrophobic interactions with three phenylalanine residues (F96, F119, and F235). **28** increased α‐tubulin acetylation and demonstrated dose‐dependent antiproliferative activity in breast cancer MCF7 cells.^375^

The most important SIRT1/2 inhibitors reported in literature so far are described in Table 2.

**Figure 11:**
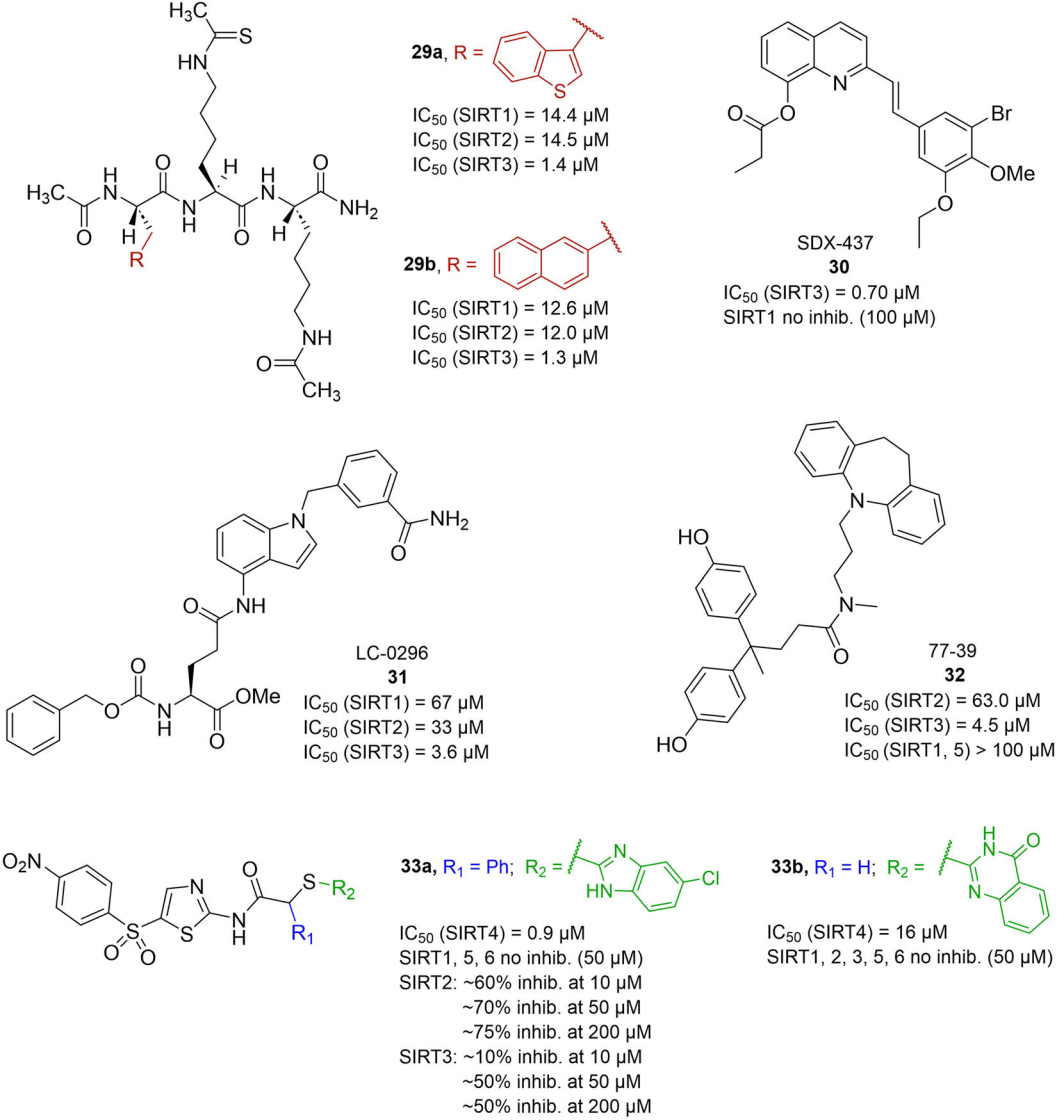
Structures and enzymatic activities of SIRT3i **29a,b**, **30‐32**, and SIRT4i **33a,b**. [Color figure can be viewed at wileyonlinelibrary.com]

Compounds **29a** and **29b** are tripeptidic single‐digit micromolar SIRT3i (Figure 11) with IC_50_ values of 1.4 and 1.3 µM, respectively, and with about 10‐fold selectivity over SIRT1 and 2.^376^ The two molecules differ in the substitution at the Cα of the *N*‐terminal residue, presenting a benzo[*b*]thiophen‐3‐yl moiety in **29a** and a naphtalen‐2‐yl substituent in **29b**. Both molecules bear the typical mechanism‐based SIRT inhibitory moiety *ε‐N*‐thioacetyl‐lysine, which is responsible for the formation of the intermediate α−1’‐*S*‐alkylamidate.^376^ Although promising, no cell‐based studies were performed for these compounds.

In 2015, Patel et al. reported the quinoline derivative SDX‐437 (**30**, Figure 11) as the first‐in‐class submicromolar small molecule SIRT3i, although they did not validate it in cells.^377^ The compound, discovered through a high‐throughput screening using self‐assembled monolayer desorption/ionization mass spectrometry (SAMDI‐MS), exhibited an IC_50_ of 0.70 µM against SIRT3 and was >100‐fold selective over SIRT1.^377^ More studies are needed to confirm the isoform‐specificity and assess the cellular activity of this molecule.

LC‐0296 (**31**, Figure 11) is an amino acid derivative displaying an IC_50_ value of 3.6 µM for SIRT3 while showing 9 and 18‐fold higher IC_50_s for SIRT1 and SIRT2, respectively. **31** was shown to inhibit the proliferation of UM‐SCC‐1 and UM‐SCC‐17B head and neck squamous cell carcinoma cells concentration‐dependently. Mechanistically, it promoted apoptosis by raising the acetylation of mitochondrial proteins and promoting ROS formation. Among SIRT3 targets, **31** reduced the acetylation of GAPDH and NDUFA9.^378^

Compound 77‐39 (**32**, Figure 11) is a low micromolar selective inhibitor of SIRT3 identified through a DNA‐encoded chemical library screening.^379^ **32** has an IC_50_ value toward SIRT3 of 4.5 μM and no activity against SIRT1, 2, and 5. **32** is well tolerated in HeLa cells where it augments the total acetylation of mitochondrial proteins and depletes ATP production. Nonetheless, target engagement studies would be necessary to further validate this compound.^379^

A target‐based virtual screening recently led to the development of compounds **33a** and **33b**, reported by Pannek and colleagues as first‐in‐class SIRT4 inhibitors endowed with IC_50_ values of 0.9 and 16 μM, respectively.^380^ These compounds showed preference for SIRT4 over other isoforms, with **33b** being the most selective over SIRT1, 2, 3, 5, and 6 (Figure 11). Notably, when tested in C2C12 mouse myoblast cells at 5, 10, and 25 μM both inhibitors dose‐dependently increased GDH activity in both whole cells and mitochondrial extracts. Moreover, they were able to rescue PDH activity after treatment of C2C12 cells with the glutamine supplement Glutamax (4 mM), which is known to inhibit PDH activity. Similar results were also obtained when mitochondrial lysates were treated with **33a** and **33b**. These results are consistent with previous studies demonstrating that SIRT4 negatively regulates GDH and PDH activities.^126^, ^127^ Finally, given the role of SIRT4 in regulating preadipocyte proliferation and differentiation, compound **33b** was tested in 3T3‐L1 adipocytes and suppressed the development of both wild type and SIRT4‐overexpressing cells at a concentration of 100 μM.^380^

The most important SIRT3i and SIRT4i identified so far are shown in Table 3.

**Table 3:** Most relevant SIRT3‐7 inhibitors.

Compd.	Molecular structure	Enzymatic activity	Cell‐based/in vivo effects	References
**31** LC‐0296	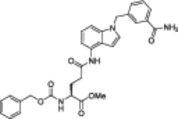	IC_50_ (SIRT1) = 67 µM IC_50_ (SIRT2) = 33 µM IC_50_ (SIRT3) = 3.6 µM	– UM‐SCC‐1 and UM‐SCC‐17B head and neck squamous cell carcinoma cells: antiproliferative activity and apoptosis induction joined to higher mitochondrial total protein acetylation and ROS levels.	[379]
**32** 77‐39	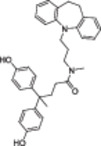	IC_50_ (SIRT2) = 63.0 µM IC_50_ (SIRT3) = 4.5 µM IC_50_ (SIRT1,5) > 100 µM	– HeLa cells: augmentation of mitochondrial proteins acetylation, depletion of ATP production.	[380]
**33b**	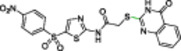	IC_50_ (SIRT4) = 16 µM SIRT1, 2, 3, 5, 6: no inhib. at 50 µM	– C2C12 mouse myoblast cells: dose‐dependent increase of GDH activity in both whole cells and mitochondrial extracts; rescue of PDH activity after treatment with the glutamine supplement Glutamax (4 mM), in both whole cells and mitochondrial extracts. – 3T3‐L1 adipocytes: suppression of the development of both wild type cells and SIRT4‐overexpressing cells at a concentration of 100 μM.	[381]
**34** MC3482	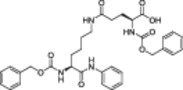	Not available	– MDA‐MB‐231 breast cancer cells: Inhibition of SIRT5 desuccinylase activity (42% inhibition at 50 µM) and mouse myoblasts without affecting its expression; no SIRT1 inhibition and 8% SIRT3 inhibition at 50 µM. – MDA‐MB‐231 breast cancer cells: and C2C12 mouse myoblasts: Increase of total protein succinylation, but not acetylation; increase of cellular ammonia and glutamate levels; autophagy and mitophagy induction.	[142]
**35 g**	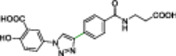	IC_50_ (SIRT5) = 7.2 µM SIRT5 ~ 75% inhib. at 50 µM SIRT1‐3 < 15% inhib. at 50 µM	HeLa: SIRT5 inhibition in cell at 250 μM according to live cell imaging experiments.	[385]
**40b**	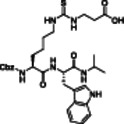	IC_50_ (SIRT5, deglutaryl.) = 0.37 µM *K*_i_ (SIRT5, deglutaryl.) = 40 nM SIRT1‐3, 6 no inhib. at 10 µM	Tested as ethyl ester (***Et‐*** **32b**). – HEK293 cells expressing FLAG‐tagged SOD1: increased succinylation of SOD1 at Lys122. – SIRT5‐dependent (OCI‐AML2 and SKM‐1) AML cells: Antiproliferative activity [IC_50_ (SKM‐1) ~ 5 µM; IC_50_(OCI‐AML2) ~ 8 µM]; >80% apoptosis induction at 5 µM (SKM‐1) and 10 µM (OCI‐AML2). – SIRT5‐independent (KG1a and Marimo) AML cells: no activity.	[166, 393]
**40d**	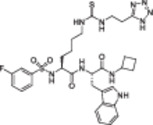	IC_50_ (SIRT5, deglutaryl.) ≤ 0.05 µM *K*_i_ (SIRT5, deglutaryl.) = 0.5 nM SIRT1‐3, 6 no inhib. at 10 µM ***He‐*** **32d** prodrug: 76% SIRT1 inhibition at 1 µM.	Tested as O‐*tert*‐butyloxycarbonyl‐N,O‐isobutyl hemiaminal (***He‐*** **32d**) prodrug. – HEK293T cells: target engagement with SIRT5 (ITDRF‐CETSA IC50 value of 0.15 μM), but also with SIRT1 (full melting curve CETSA). – SIRT5‐dependent AML cells: antiproliferative activity [IC_50_(SKM‐1) = 9 µM; IC_50_(OCI‐AML2) = 20 µM; IC_50_(MOLM‐13) = 24 µM].	[394]
**41c** DK1‐04	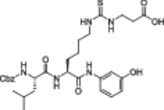	IC_50_ (SIRT5, desuccinyl.) = 0.34 µM SIRT1‐3, 6 no inhib. at 83.3 µM	Tested as ethyl ester (***Et‐*** **33c**) or aceto‐methoxy (***Ac‐*** **33c**) prodrug. – MCF7 breast cancer cells: raise of global succinylation levels induced by both prodrugs. – MCF7 and MDA‐MB‐231 breast cancer cells: antiproliferative activity and suppression of anchorage‐independent growth by both prodrugs, with ***Et‐*** **33c** being the most active. – Genetically engineered and xenograft (MDA‐MB‐231) breast cancer mouse models: tumor growth inhibition by ***Et‐*** **33c** (only tested molecule).	[396]
**44b**	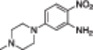	IC_50_ (SIRT6, deacetyl.) = 4.93 µM SIRT1,3 no inhib. at 200 µM	– BxPC‐3 PDAC cells: Dose‐dependent increase of H3K9/K18 acetylation. – Mouse model of type 2 diabetes: higher GLUT‐1 expression and reduced blood glucose levels.	[401]
**45c**	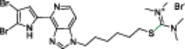	IC_50_ (SIRT6, deacetyl.) = 7.46 µM IC_50_ (SIRT1) = 80.5 µM IC_50_ (SIRT2) = 92.2 µM SIRT3,5 no inhib. at 200 µM	– HeLa cells: confirmed SIRT6 target engagement at 25 μM (CETSA). – PDAC cells: cell cycle arrest, apoptosis, and block of cell proliferation with IC_50_ values of 7–9 μM. – HUVECs: migration inhibition, downregulation of angiogenesis‐related proteins (N‐cadherin, p‐VEGFR2, VEGF, and HIF‐1α – PDAC mouse xenografts: increases gemcitabine efficacy and impairs cancer angiogenesis by downregulating HIF‐1α and CD31.	[402, 403]
**46** JYQ‐42	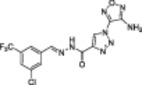	IC_50_ (SIRT6, deacetyl.) = 2.33 µM IC_50_ (SIRT2) = 87.2 µM SIRT1,3,5,7 no inhib. at 100 µM	– PDAC cells: dose‐dependent increase of H3K9, H3K18, and H3K56 acetylation without affecting SIRT6 expression; impaired cell migration and secretion of pro‐inflammatory cytokines (IL6, IL8, and TNF‐α), while not affecting cell proliferation at concentrations up to 20 μM.	[404]
**47b** A127‐(CONHPr)‐B178	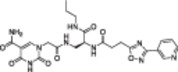	IC_50_ (SIRT6, demyristoyl.) = 6.7 µM SIRT1‐3,5,7 < 10% inhib. at 10 µM	– HUVECs: senescence promotion and dose‐dependent increase in TNF‐α levels.	[405]
**48a**	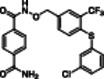	IC_50_ (SIRT6, deacetyl.) = 0.98 µM *K*_*d*_ (SIRT6) = 9.46 μM SIRT1‐3 < 25% inhib. at 100 µM	– Pancreatic cancer cells: dose‐dependent increase of acetylated H3K9Ac, H3K18Ac, and H3K56Ac levels with no cytotoxic effects at 100 μM. – Pancreatic cancer mouse xenograft models: reduction of liver metastatic nodules and increase H3K9Ac, H3K18Ac, and H3K56Ac levels.	[406]
**50** ID:97491	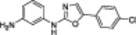	IC_50_ (SIRT7) = 0.325 µM	– MES‐SA human uterine sarcoma cells: dose‐dependent raise in p53 acetylation and phosphorylation; promotion of caspase‐induced apoptosis by raising the Bax and p21 levels; antiproliferative activity. – Uterine sarcoma xenograft mouse models: dose‐dependent tumor growth suppression.	[408]
**52**	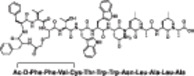	IC_50_ (SIRT7) = 2.7 μM *K*_*i*_ (SIRT7) = 0.53 μM IC_50_ (SIRT6) ~ 149 μM SIRT1,2,3,5,6: no inhib. (10 µM)	– HEK293T cells: confirmed target engagement through CETSA (10 μM); increase of H3K18 acetylation at 1 and 10 μM with no influence on H3K27 and H3K36 acetylation status at the same concentrations.	[35]

Abbreviations: AML, acute myeloid leukemia; Bax, Bcl2‐associated X protein; CETSA, cellular thermal shift assay; HUVEC, human umbilical venous endothelial cell; ITDRF‐CETSA, isothermal dose–response fingerprinting cellular thermal shift assay; ROS, reactive oxygen species; SOD1, superoxide dismutase 1; TNF‐α, tumor necrosis factor α.

**Figure 12:**
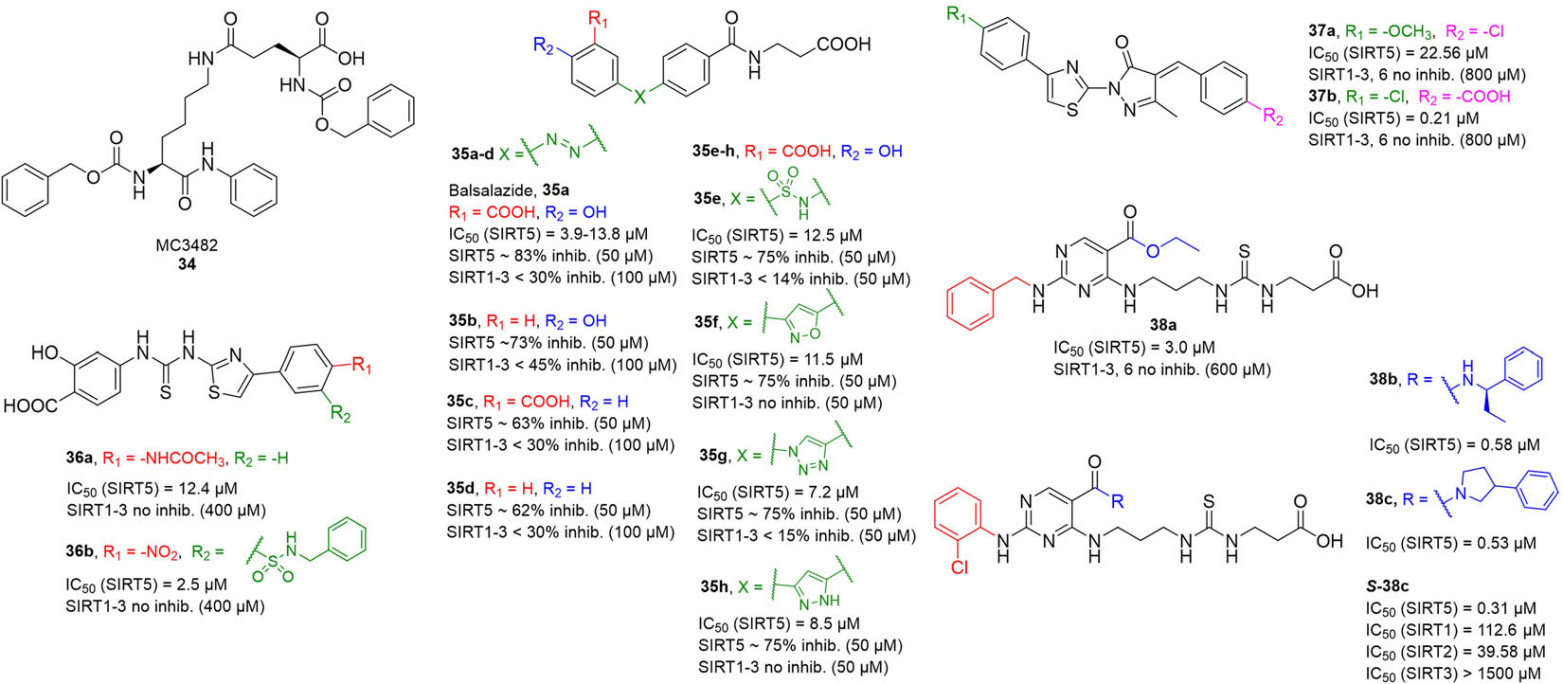
Structures and enzymatic activities of SIRT5i **34**, **35a‐h**, **36a,b**, **37a,b**, and **38a‐c**. [Color figure can be viewed at wileyonlinelibrary.com]

MC3482 (**34**, Figure 12) is a *ε‐N*‐glutaryllysine derivative inhibiting SIRT5 desuccinylase activity in both mouse myoblasts (C2C12) and human TNBC cells (MDA‐MB‐231), with no influence on its expression.^142^ In MDA‐MB‐231, **34** inhibited SIRT5 dose‐dependently, reaching 42% inhibition at 50 µM. Its specificity was also assessed in the same cell line, indicating that **34** does not inhibit SIRT1 and displays only 8% SIRT3 inhibition at 50 µM. In both MDA‐MB‐231 and C2C12 cell lines, **34** determined an increase in protein succinylation at 50 µM, while not affecting acetylation levels. Compound **34** also raised cellular glutamate and ammonia levels, because of increased succinylation and resultant activation of glutaminase. Notably, augmented glutamate levels triggered mitophagy and autophagy.^142^ Administration of **34** in the initial phases of preadipocyte differentiation was recently found to stimulate the expression of mitochondrial biogenesis and brown adipocyte factors.^381^ Overall, this study indicates that SIRT5 inhibition enhances brown adipogenesis and it might be a strategy to stimulate BAT and counteract obesity.

Balsalazide (**35a**, Figure 12) was originally identified as a low micromolar SIRT5i (IC_50_ = 3.9 µM) via a high‐throughput screening by Guetschow et al.^382^ **35a** is an approved anti‐inflammatory drug currently used for the treatment of inflammatory bowel disease. To clarify how this compound inhibits SIRT5, Glas and co‐workers performed a docking analysis comparing the predicted binding mode of **35a** with the interactions of a co‐crystallized succinyl‐lysine based peptide^27^ in the presence of NAD^+^. This analysis indicated that the β‐alanine‐derived side chain of **35a** and its carboxylate group strongly contribute to the affinity and, consequently, to SIRT5 inhibition.^383^ Subsequently, they synthesized a series of 13 analogs through the introduction of different modifications to the *N*‐aroyl‐β‐alanine side chain and by removing various functional groups, such as the hydroxy and carboxy ones, from the salicylic portion. These analogs were tested through a Fluor de Lys assay. Under these conditions, **35a** showed an IC_50_ for SIRT5 desuccynilase activity of 5.3 μM and 83% inhibition at 50 µM, along with selectivity over SIRT1‐3, but its derivatives were all less potent than the parent compound. SAR analysis indicates that the modifications of the salicylic acid moiety are partially tolerated, since compounds **35b**, **35c**, and **35d** (Figure 12) show 73%, 63%, and 62% SIRT5 inhibition at 50 µM, respectively. However, even small modifications of the β‐alanine side chain led to a complete loss of potency.^383^ Furthermore, biochemical assays indicated that **35a** and **35b** do not compete with NAD^+^ nor with the synthetic substrate ZKsA. Despite poorly soluble in water and prone to enzymatic degradation, **35a** still represents a lead molecule for SIRT5i development.^383^ The same group recently implemented additional modifications to the core of **35a** to improve its pharmacokinetics, resulting in the preparation of derivatives **35e–h**.^384^ The azo group was replaced by a sulfonamide (**34e**) or the heteroaryl groups isoxazole (**35f**), 1,2,3‐triazole (**35g**), and pyrazole (**35h**) (Figure 12). When tested at 50 µM, the compounds reduced SIRT5 desuccinylase activity by 75% (**35e**), 80% (**35f**), and 84% (**35g** and **35 h**), while **35a** demonstrated 89% SIRT5 inhibition under the same assay conditions. dose–response curves were also generated for **35e**, **35f**, **35g**, and **35h** which revealed IC_50_ values of 12.5, 11.5, 7.2, and 8.5 µM, respectively, whereas **35a** exhibited an IC_50_ value of 13.8 µM. This data indicates that substituting the azo group with sulfonamide or nitrogen‐rich heteroaryl rings enhances the inhibitory effect. Compounds **35a** and **35e‐h** demonstrated selectivity toward SIRT1‐3 at 50 µM. However, chemoproteomic analyses indicated that **35a**, **35g**, and **35h** bind to enzymes other than SIRT5, specifically glutaryl‐CoA‐dehydrogenase (GCDH) and nucleoside diphosphate kinase (NME4). Chemoproteomic competition assays indicated that the most selective compound is **35g**, with EC_50_ values for SIRT5 ~49.5‐ and ~6.5‐fold lower than the ones for GCDH and NME4, respectively.^384^ Finally, live cell imaging in HeLa^385^ cell line demonstrated that both **35g** and **35a** inhibit SIRT5 in cells, with **34a** being active at 600 μM, while **35g** was active at 250 μM. This data indicates that **35g** is likely more cell permeable than **35a**, thus suggesting that the triazole moiety increases cell permeability compared with the azo linker. No target engagement studies were performed for compounds **35b‐f**.^384^

The recently reported SIRT5i **36a, b** share the same salicylic acid moiety as **35a‐g** (Figure 12). These compounds are the most potent ones of a series for which selectivity over other SIRT isoforms was tested.^386^ Specifically, **36a,b** exhibited an IC_50_ value for SIRT5‐mediated desuccinylation of 12.4 and 2.5 μM, respectively, while not showing any influence on SIRT1‐3 activity at concentrations up to 400 μM. Nevertheless, no cellular studies were conducted with these inhibitors, so further investigations are needed.^386^

The Yanghan group later developed a series of pyrazolone derivatives exhibiting SIRT5‐inhibiting properties. Among them, the hit compound **37a** and its derivative **37b** showed selective inhibition of SIRT5 desuccinylase activity, with **37b** being 100‐fold more potent than **37a** [IC_50_(**37a**) = 22.56 μM; IC_50_(**37b**) = 0.21 μM)] (Figure 12).^387^ Neither molecule affected SIRT1‐3 and SIRT6 activity at concentrations up to 800 μM. Notably, the activity of **37b** decreases as the concentration of succinyl‐lysine substrate increases, while it is not affected by NAD^+^ concentrations, thus indicating that **37b** competes with SIRT5 succinylated substrate, but not with NAD^+^. Also in this case, cell‐based studies were not performed, hence further validation is necessary.^387^

Recently, the Yang group developed a series of thioureido‐propanoic acid analogs, among which the 2,4,5‐trisubstituted pyrimidine derivative **38a** was the most potent and selective, with an IC_50_ for SIRT5 desuccinylation of 3.0 μM and no inhibition of SIRT1‐3 and 6 at 600 μM (Figure 12). No cell‐based assays were performed for this compound.^388^ Based on this structure, they developed further 2,4,5‐trisubstituted pyrimidine analogs, yielding SIRT5i endowed with low‐micromolar^389^ to submicromolar inhibition.^390^ These include **38b** (IC_50_ = 0.58 μM), with an α‐ethyl‐benzylamide moiety at the C5 position of the pyrimidine, and **38c** (IC_50_ = 0.53 μM), which has a 2‐phenyl‐pirrolidine‐amide function at pyrimidine C5, and its *S*‐enantiomer ***S*** **−38c** (IC_50_ = 0.31 μM) (Figure 12). ***S*** **−38c** was tested for its selectivity over SIRT1‐3, with IC_50_ values of 112.6 μM, 39.58 μM, and >1.5 mM, respectively. Mechanistic studies indicated that ***S*** **−38c** competes with the succinylated substrate rather than NAD^+^. No cell‐based experiments were reported; thus, target engagement was not confirmed. Nevertheless, the authors showed that ***S*** **−38c**, at doses of 20–60 mg/kg, could mitigate kidney dysfunction and pathological injury in both lipopolysaccharide (LPS)‐ and cecal ligation/perforation (CLP)‐induced septic AKI mice, although the effect on global protein succinylation was not evident. Finally, ***S*** **−38c** exhibited favorable pharmacokinetic characteristics when administered intravenously.^390^

**Figure 13:**
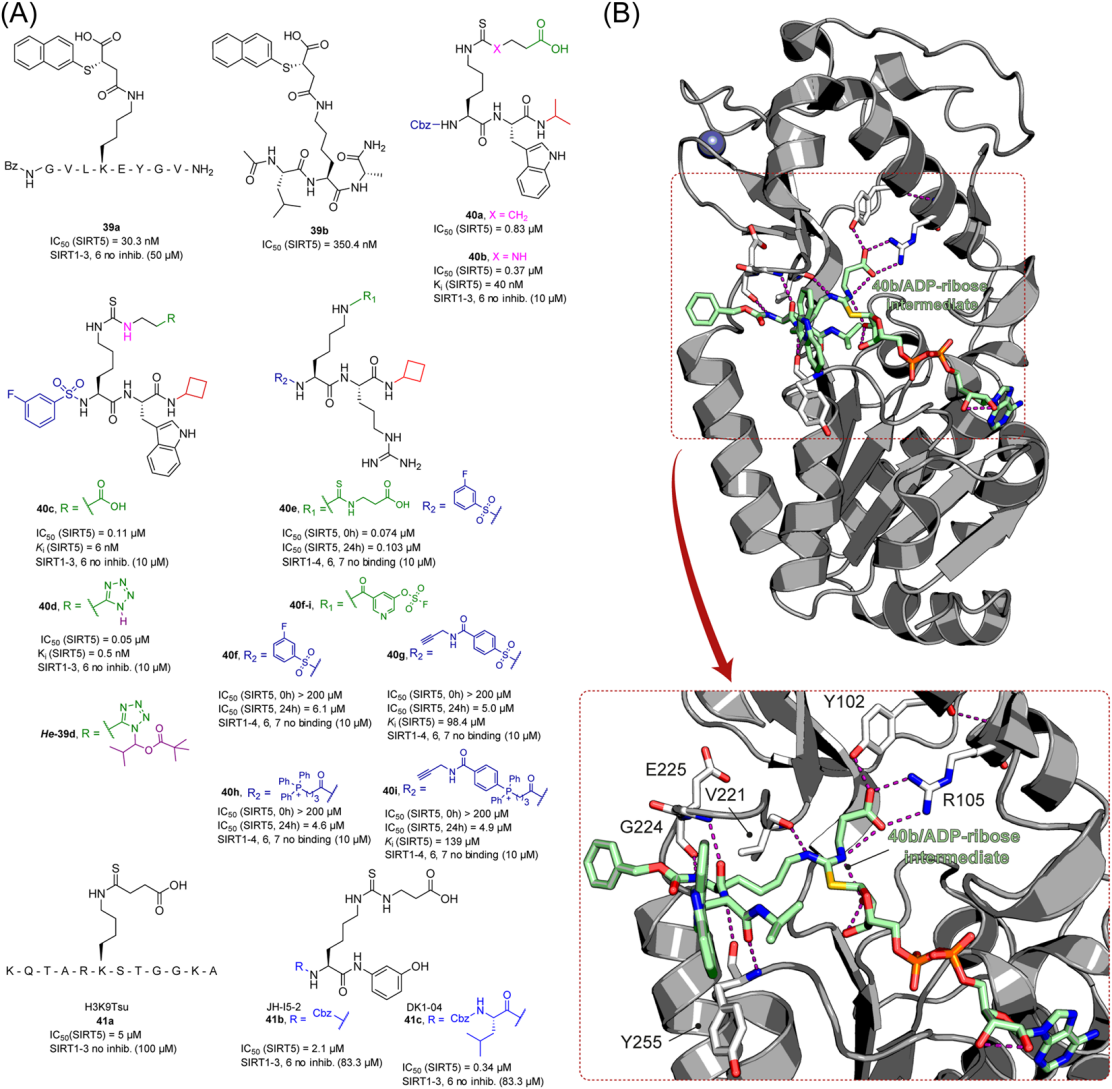
(A) Structures and enzymatic activities of SIRT5i **39a,b**, **40a‐i**, and **41a‐c**. (B) hSIRT5/ADP‐ribose/**40b** co‐crystal structure (PDB ID: 6EQS) showing the formation of the **40b**/ADP‐ribose‐1′‐thioimidate adduct (green sticks). The enzyme is shown as gray cartoon and the key interacting residues are shown as white sticks. Hydrogen bonds involving **40b**/ADP‐ribose and residues Y102, R105, V221, G224, E225, Y255 are depicted as purple dotted lines. [Color figure can be viewed at wileyonlinelibrary.com]

Recently, Kalbas and colleagues developed novel peptide‐based nanomolar SIRT5i via a structure‐based optimization effort.^391^ Starting from the inspection of the crystal structure of zebrafish SIRT5 bound to the 3‐phenylsuccinyl‐carbamoyl phosphate synthetase 1 (CPS1)‐derived peptide substrate, different (*S*)−3‐(2‐naphthylthio)succinyl derivatives were prepared and assessed. Among them, the peptide **39a** (Figure 13A) showed a potent SIRT5 inhibition (IC_50_ = 30.3 nM) and was competitive against the peptide substrate, while it did not inhibit SIRT1‐3 and 6 up to 50 µM. The shortened tripeptide derivative **39b** (Figure 13A) was less potent [IC_50_(SIRT5) = 350.4 nM], but possessed a more drug‐like structure and, although not tested in cell‐based assays, it represents a good starting point for further drug development.^391^

The Olsen group recently developed a series of submicromolar SIRT5i starting from a ε‐*N*‐thioglutaryllysine core (Figure 13A).^392^ Among the synthesized derivatives, the *N*‐terminal carbobenzyloxy (Cbz)‐protected **40a** and **40b** were co‐crystallized with zebrafish (**40a** and **40b**) and human SIRT5 (**40b** only). The two molecules differ in the chemistry of lysine derivatization, consisting of a thioamide in **40a** and a thiourea in **40b**. The measured IC_50_ values for SIRT5 deglutarylase activity were 0.83 (**40a**) and 0.37 µM (**40b**). However, these should be taken with caution since IC_50_ determinations are based on equilibrium experiments while these molecules are mechanism‐based inhibitors requiring the formation of a stable covalent intermediate. For the most promising compounds, the authors also obtained *K*_*i*_ values via continuous flow measurements, which provide a kinetic evaluation and a better estimate of the inhibitor potency. Specifically, the measured *K*_*i*_ for **40a** was 20 nM, while it was 40 nM for **40b**, with both compounds exhibiting a slow and tight binding. From the SIRT5/ADP‐ribose/**40b** co‐crystal structure (Figure 13B), it is apparent that both compounds form a stalled intermediate with ADP‐ribose and indicate that the carboxylate group forms crucial hydrogen bonds with Y102 and R105, while the Cbz portion is not involved in any interaction. Hence, this was replaced by various groups, including a 3‐fluorobenzenesulfonamide that led to the compound **40c** (Figure 13A), that resulted the most potent of the series (IC_50_ = 0.11 μM and *K*_*i*_ = 6 nM). **40b** and **40c** were also selective over SIRT1‐3 and 6, while the selectivity evaluation was not performed for **40a**.^392^ To mask the negative charge of the carboxylic group and increase cellular permeability, ethyl ester prodrugs of **40b** and **40c** were subsequently prepared leading to compounds ***Et‐*** **40b** (called NRD167) and ***Et‐*** **40c** (called NRD139). To confirm cellular inhibition of SIRT5, HEK293 cells expressing FLAG‐tagged SOD1, a known SIRT5 substrate, were incubated for 18 h with ***Et‐*** **40b** (10 μM). Immunoprecipitation with an antibody specific for SOD1‐K122succinyl demonstrated an increase of succinylation, in line with SIRT5 inhibition.^166^ ***Et‐*** **40c** was later tested in an isothermal dose–response fingerprinting cellular thermal shift assay (ITDRF‐CETSA) in HEK293T cells and was shown to stabilize SIRT5 with an EC_50_ value of 0.25 μM, thus confirming the target engagement.^393^ ***Et‐*** **40b** and ***Et‐*** **40c** were evaluated on a panel of different AML cell lines, including both cells (e.g., OCI‐AML2 and SKM‐1) where the proliferation was dependent on SIRT5 activity, and others (e.g., KG1a and Marimo) where the proliferation was SIRT5‐independent. Both molecules reduced cell proliferation only in SIRT5‐dependent cells.^166^ Specifically, ***Et‐*** **40b** exhibited IC_50_ values of 5–8 µM, lower than ***Et‐*** **40c** which possessed IC_50_ values between 10 and 20 µM. Consistent with this, both compounds triggered apoptosis only in SIRT5‐dependent cells, with ***Et‐*** **40b** inducing more than 80% apoptosis at 5 or 10 µM (in SKM‐1 and OCI‐AML2, respectively), while ***Et‐*** **40c** reached the same result only in SKM‐1 cells at 20 µM. Notably, ***Et‐*** **40b** administration phenocopied SIRT5 knockdown in the tested cell lines. Furthermore, mice injected with cells from AML patients that were pretreated with ***Et‐*** **40b** exhibited higher survival than control.^166^ They further developed a series of compounds by applying bioisosteric substitution to the carboxylic acid moiety of **40 c**.^393^ This led to compound **40d**, bearing a tetrazole ring as carboxylic acid bioisostere (Figure 13A), which was tested for its inhibition of SIRT5 deglutarylase activity and exhibited an IC_50_ ≤ 0.05 µM, while no activity toward SIRT1‐3 and 6 was detected up to 10 µM. Kinetic measurements indicated slow, tight‐binding kinetics with a *K*_i_ value of 0.5 nM. Given its low cellular permeability, the authors prepared a prodrug by masking the tetrazole with an *O*‐*tert*‐butyloxycarbonyl‐*N,O*‐isobutyl hemiaminal moiety, yielding compound ***He*** **−40d**. Interestingly, this prodrug showed 76% SIRT1 inhibition at 1 µM, hence the masking moiety decreases the compound selectivity. ITDRF‐CETSA yielded an EC_50_ value for SIRT5 stabilization in HEK293T cells of 0.15 μM, indicating target engagement. Full melting curve CETSA run in HEK293T cells confirmed SIRT5 cellular engagement of ***He*** **−40d**, but also demonstrated significant engagement with SIRT1, but not SIRT3. The observed SIRT1 binding in cells suggests only a partial hydrolysis of the tetrazole‐based masking group. ***He*** **−40d** was then tested in SIRT5‐dependent SKM‐1 AML, OCI‐AML2, and MOLM‐13 cells, showing higher antiproliferative efficacy than ***Et*** **−40c** in all cell lines, with IC_50_ values between 9 and 24 µM.^393^

To obtain SIRT5 covalent inhibitors, the Olsen group has developed aryl fluorosulfate‐containing peptidomimetics based on the structure of compound **40c**.^394^ To increase the water solubility, the Trp group was replaced with Arg, yielding compound **40e**. Compound **40f** was obtained by replacing the thiourea‐containing moiety of **40e** with a pyridin‐3‐yl fluorosulfate group, whereas **40g‐i** possess the same pyridin‐3‐yl fluorosulfate moiety, but with different substitutions at the *N*‐terminus (Figure 13A). Notably, **40h** has an *N*‐terminal mitochondria‐targeting TPP group, while **40i** has a combination of the **40g** and **40h** substitutions at the *N*‐terminus. While compound **40e** did not exhibit time‐dependent inhibition of SIRT‐mediated deglutarylation, **40f‐i** were inactive at *t* = 0 h, while they all reached low‐micromolar inhibition after 24 h incubation, thus suggesting a covalent binding mode (Figure 13A). LC‐MS experiments confirmed that **40f‐i** form covalent adducts with SIRT5 and the presence of NAD^+^ was shown to augment the covalent adduct formation rate, indicating that these inhibitors function via a process that involves the SIRT5 active site. Kinetic analyses yielded *K*_i_ values for on **40g** and **40i** of 98.4 and 139 μM, respectively. Further LC‐MS analyses using compounds **40f** and **40g** and the Y102F, Y104F, and R105A SIRT5 mutants suggested that R105 is essential for covalent adduct formation, while both Y102F and Y104F mutants were still able to form covalent adducts. Finally, LC‐MS/MS experiments using **40f** indicated that the compound preferentially forms covalent adducts with Y76 and Y102, while Y104 is targeted only in the case of the SIRT5 Y102F mutant. In‐gel fluorescence imaging investigations revealed that **40g** and **40i** were exclusively capable of forming covalent adducts with SIRT5, but not SIRT1‐4, SIRT6, or SIRT7 (at 10 μM). Compounds **40g** and **40i** were also able to pull down SIRT5 from HEK293T cells in a click chemistry‐based experiment using azide‐containing biotin followed by enrichment with streptavidin‐coated beads.^394^ Furthermore, evaluation in HeLa cells revealed that both compounds **40f** and **40g** inhibited SIRT5 activity in cells at a concentration of 200 µM, with **40g** being more potent than **40f**. The latter was tested at 200 μM and exhibited negligible impact on cell viability in various AML cell lines, except for the Jurkat cell line. Finally, **40g** was well tolerated in mice at a dose of 12 mg/kg, while **40i** was toxic, most likely due to the mitochondria‐targeting moiety. Despite the very rapid clearance of **40g**, SIRT5 could be pulled down from mouse hearts treated with **40g** using an azide streptavidin/biotin‐based assay, similar to the method described above for HEK293T cells. These results suggest that **40g** may covalently bind SIRT5 in mouse hearts.^394^

A recent report described the development of peptide‐based SIRT5i possessing a thiourea moiety.^395^ Starting from H3K9 thiosuccinylated peptide H3K9Tsu (**41a**), which selectively inhibited SIRT5 in vitro [IC_50_(SIRT5) = 5 µM, no inhibition of SIRT1‐3 at 100 µM],^396^ Abril and colleagues gradually compacted the peptide to the thiourea derivative JH‐I5‐2 (**41b**), also protected with a Cbz group at the *N‐*terminus [IC_50_(SIRT5) = 2.1 μM] (Figure 13A). The addition of a Cbz‐protected leucine residue on the *N*‐terminus yielded DK1‐04 (**41c**), which exhibited an IC_50_ value against SIRT5 of 0.34 μM.^395^ According to kinetic studies, the molecules inhibit SIRT5 forming a covalent 1’‐*S*‐alkylamidate intermediate, while no inhibition of SIRT1‐3 and SIRT6 was observed up to 83.3 μM. In this case, to increase cell permeability, two different pro‐drug approaches were explored by masking the carboxylic group with ethyl ester (***Et‐*** **41b** and ***Et‐*** **41c**) or aceto‐methoxy (***Am‐*** **41b** and ***Am‐*** **41c**) groups. In MCF7 breast cancer cells, all compounds raised global lysine succinylation, but no specific studies were performed to confirm the SIRT5 binding of these compounds at cellular level. ***Et‐*** **41c** was the most active molecule in terms of suppression of anchorage‐independent growth of MCF7 and MDA‐MB‐231 breast cancer cells. ***Et‐*** **41c** also suppressed tumor growth in genetically engineered and MDA‐MB‐231 xenograft mouse models of breast cancer.^395^

The most significant SIRT5i identified so far are reported in Table 3.

**Figure 14:**
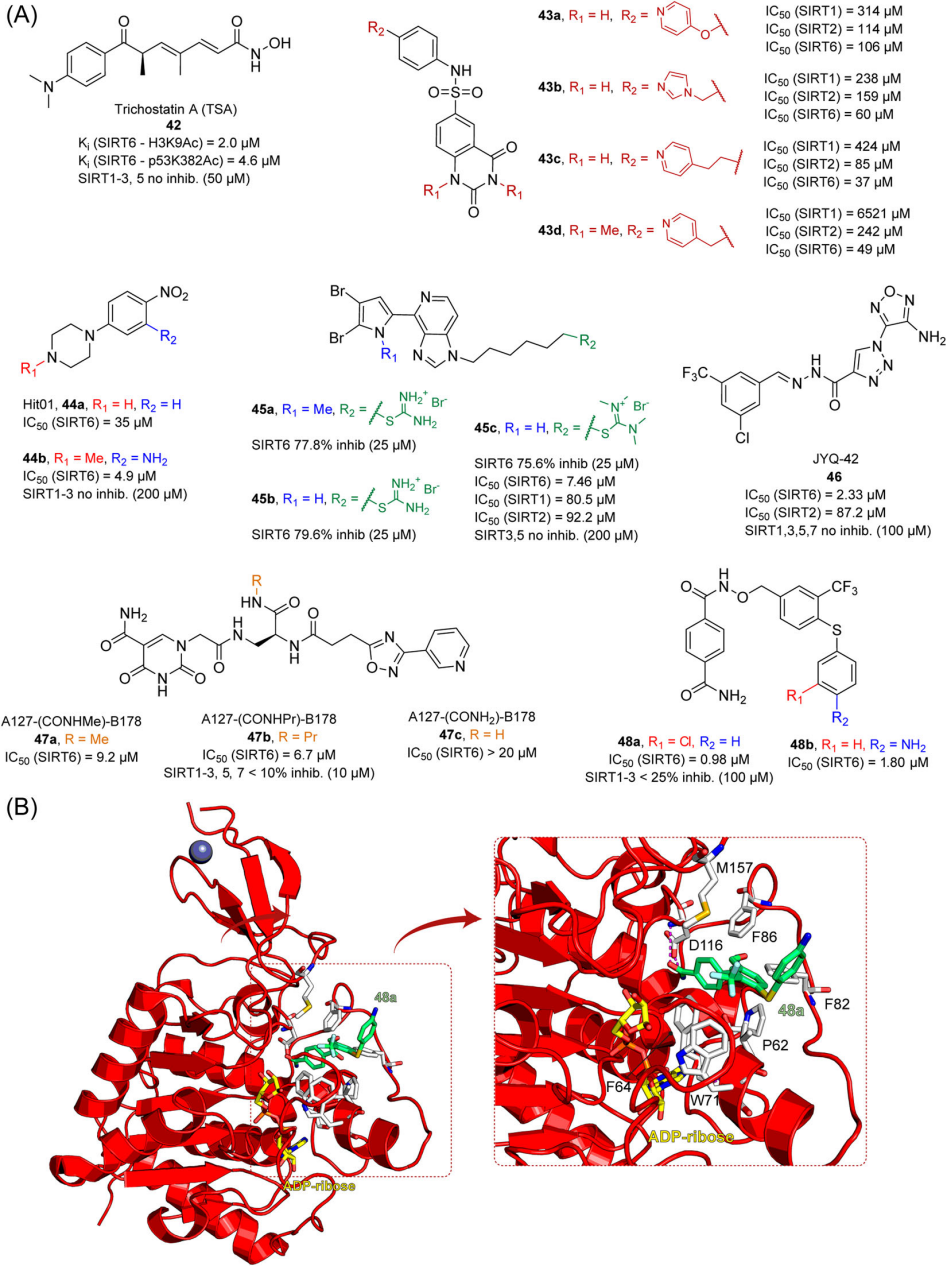
(A) Structures and enzymatic activities of SIRT6i **42**, **43a‐d**, **44a,b**, **45a‐c**, **46**, **47a‐c**, and **48a,b**. (B) hSIRT6/ADP‐ribose/**48b** co‐crystal structure (PDB ID: 8I2B) showing the key interactions between the small molecule and the enzyme, including the hydrogen bond between the amide group of **48b** and the carboxylic acid of D116 side‐chain (purple dotted line). hSIRT6 is colored in red with key residues shown as white sticks. Compound **48b** is depicted as green sticks, and ADP‐ribose is depicted as yellow sticks. [Color figure can be viewed at wileyonlinelibrary.com]

Trichostatin A (TSA, **42**, Figure 14A) has been recently reported as a SIRT6i, showing selectivity over SIRT1‐3 and 5, although it possesses nanomolar inhibitory activity toward Zn^2+^‐dependent HDACs. Its inhibitory potency against SIRT6 has been evaluated in terms of *K*_*i*_. When using H3K9Ac peptide, the *K*_*i*_ against SIRT6 deacetylation was 2.02 μM, while it was 4.62 μM when using p53K382Ac peptide as a substrate.^397^ According to kinetic analysis, **42** is a competitive inhibitor of the acetylated peptide, but does not compete with NAD^+^. You et al. also shed light on the **42**‐SIRT6 interaction by resolving the crystal structure of the ternary complex SIRT6/ADP‐ribose/**42**, revealing that **42** binds to the nicotinamide pocket and the acyl channel of the enzyme active site.^398^ Incubation of HEK293T cells with **42** indicated that it dose‐dependently increases the acetylation of the SIRT6 substrate p53 at Lys382 as well as H3K9, albeit to a lesser extent.^397^

Quinazolinedione derivatives **43a‐d** are SIRT6i active in the mid‐micromolar range (IC_50_ values of 106, 60, 37, and 49 µM, respectively) (Figure 14A).^200^, ^399^ Among them, **43d** was the most selective molecule, with 133‐fold selectivity over SIRT1, fivefold selectivity over SIRT2, and no inhibitory activity against Zn^2+^‐dependent HDACs. **43c** is the most potent inhibitor of the series, indicating that possessing a longer aliphatic spacer between the aromatic groups is favorable for SIRT6 inhibition, though slightly impairing selectivity, since **43c** is only 11‐fold selective over SIRT1 and 2.3‐fold selective over SIRT2. When tested in cells, these derivatives increased H3K9 acetylation in BxPC3 PDAC cells, but only **43a**, **43c** and **43d** augmented glucose uptake. In addition, **43b** and **43c** increased the sensitivity of BxPC3 cells to gemcitabine, a first‐in‐line drug approved for PDAC treatment. Compound **43c** also increased the sensitivity of the PDAC cell line Capan‐1 to the treatment with the poly(ADP‐ribose) polymerase (PARP) inhibitor olaparib.^399^ Finally, **43a** was shown to repress muscle atrophy development in mice.^199^

To obtain new potential SIRT6i, Sun et al. screened a chemical library containing about 2000 compounds, identifying 1‐(4‐nitrophenyl)piperazine (Hit01, **44a**) as a hit fragment compound [IC_50_(SIRT6) = 35 μM].^400^ Structural optimization on **44a** led to the identification of 5‐(4‐methylpiperazin‐1‐yl)−2‐nitroaniline (**44b**, Figure 14A), which bears an amine and a nitro group at the C3 and C4 positions of the phenyl ring, respectively, and a methyl group on the *N*1 of the piperazine. **44b** showed an IC_50_ against SIRT6 of 4.9 μM along with selectivity over SIRT1‐3 and HDAC1‐11 at 200 µM. When tested in BxPC‐3 PDAC cells, this molecule increased both H3K9 and H3K18 acetylation levels in a concentration‐dependent fashion. Furthermore, **44b** reduces glucose blood levels in a type 2 diabetes mouse model by increasing the expression of the glucose transporter GLUT1.^400^

Ageladine A, a marine‐derived inhibitor of metalloproteinases, was the starting scaffold for the development of SIRT6i **45a‐c** (Figure 14A).^401^ In an initial screening, **45a‐c** were shown to inhibit SIRT6 deacetylase activity by 77.8%, 79.6%, and 75.6%, respectively, at 25 μM. Nevertheless, **45c** was the only compound that could increase H3K9 acetylation in HeLa cells at 2.5 and 5 μM, thus suggesting that it is the only cellularly active inhibitor. The calculated IC_50_ and *K*_*d*_ values for **45c** are 7.46 and 16 μM, respectively, and it was shown that **45c** does not compete with acetylated substrate nor NAD^+^, thus acting as a noncompetitive inhibitor. Moreover, **45c** is selective over SIRT1‐3, 5 and HDAC1‐11. Specifically, it did not affect the activity of SIRT3, 5 and HDAC1, 2, 4, 5, 7, and 9–11 at 200 μM, while exhibited IC_50_ values for SIRT1, 2 and HDAC3, 6, 8 between 80 and 112 μM. CETSA experiments performed at 25 μM in HeLa cells confirmed target engagement.^401^ Further experiments in PDAC cells showed that **45c** causes cell cycle arrest and apoptosis, blocks cell proliferation with IC_50_ values of 7–9 μM,^401^ and inhibits the migration of human umbilical venous endothelial cells (HUVECs) by downregulating angiogenesis‐related proteins such as *N*‐cadherin, p‐VEGFR2, VEGF, and HIF‐1α.^402^ Finally, **45c** was well tolerated in mice, sensitized PDAC mouse xenografts to gemcitabine treatment,^401^ and impaired cancer angiogenesis by downregulating HIF‐1α and CD31, a biomarker for neovascularization.^402^

By employing a hybrid computational and experimental strategy, Zhang et al. identified an allosteric site in SIRT6 that becomes available only following NAD^+^ binding, and developed the allosteric inhibitor JYQ‐42 (**46**, Figure 14A).^403^ **46** inhibited SIRT6‐mediated deacetylation with an IC_50_ value of 2.33 μM and exhibited noncompetitive behavior toward the acetylated substrate and NAD^+^. Moreover, SPR and biolayer interferometry experiments yielded *K*_*d*_ values of 22.1 and 13.2 μM, respectively. A further screening indicated that **46** does not affect the activity of SIRT1, 3, 5, and 7 and HDAC1‐11 at 100 μM, while it weakly inhibited SIRT2 with an IC_50_ value of 87.2 μM. Docking and single‐point mutation analysis suggest that **46** does indeed bind to the identified allosteric pocket. **46** was then tested in BXPC‐3 and MiaPaCa‐2 PDAC cells, where it showed a dose‐dependent increase of H3K9, H3K18, and H3K56 acetylation without affecting SIRT6 expression. Notably, **46** impaired cell migration and secretion of pro‐inflammatory cytokines (IL6, IL8, and TNF‐α), while not affecting cell proliferation at concentrations up to 20 μM.^403^

In 2019, Yuen et al. reported the first DNA‐encoded chemical library designed to target NAD^+^ binding pockets (NADEL) and able to sample the chemical binder space of enzymes with ADP‐ribosyl transferase activity.^404^ They screened NADEL against SIRT6 and identified a SIRT6 ligand composed of a 5‐aminocarbonyluracil (A127) moiety combined with a 3‐pyridinyl‐1,2,4‐oxadiazole scaffold (B178). Starting from this new ligand, they synthesized the carboxamide derivatives A127‐(CONHMe)‐B178 (**47a**), A127‐(CONHPr)‐B178 (**47b**), and A127‐(CONH_2_)‐B178 (**47c**) (Figure 14A), which exhibited IC_50_ values against SIRT6 demyristoylase activity of 9.2, 6.7, and >20 μM, respectively. These data indicate that a bulkier substituent on the carboxyamide containing linker moiety is favorable for SIRT6 inhibition. **47b** was selective over SIRT1‐3, 5, and 7, while the other compounds were not tested for selectivity. In a docking analysis, it has been shown that the A127 moiety interacts with the C‐pocket, while the B178 portion interacts with the ribose‐binding site. At cellular level, **47b** showed facilitated senescence induction and dose‐dependently increased TNF‐α levels in HUVECs. Nevertheless, no SIRT6‐specfic assays were performed in cells.^404^

Recently, a combination of computational and experimental approaches based on virtual screening and structure‐guided compound design led to the development of the allosteric SIRT6 inhibitors **48a** and **48b**, which inhibited SIRT6 deacetylase activity with IC_50_ values of 0.98 and 1.80 μM, respectively (Figure 14A).^405^ Moreover, **48a** exhibited a *K*_*d*_ value of 9.46 µM and <25% inhibition of SIRT1‐3 and HDAC1‐11 at 100 μM. The authors also managed to obtain the co‐crystal structure of SIRT6 bound to **48b** and ADP‐ribose. Notably, **48b** was shown to occupy the same binding site as the SIRT6 activator **6a**, which is the allosteric site at the end of the acyl‐binding channel (Figure 14B). Differently from **6a**, compound **48a** did not form a hydrogen bond with Pro62, but its amide nitrogen engages in a hydrogen bond with the carboxy group of the side chain of Asp116, which may account for the opposite effect caused by the two molecules. Moreover, the benzamide portion enters the hydrophobic cavity formed by Phe64, Phe82, Phe86, and Ile61, and the thioether portion points toward a solvent‐exposed area, where the phenyl ring and the trifluoromethyl group interact with Val70, Trp71, and Met157 (Figure 14B). In line with these observations, mutation of Asp116 was detrimental for compound activity, and further mutation experiments suggested that Phe86, but not Phe64 or Phe82, is responsible for most of the π‐stacking interactions. Compound **48a** was also shown not to compete with SIRT6 substrates or cofactors, thus confirming the allosteric mode of action of this compound series. Furthermore, when tested at 0–20 μM, **48a** dose‐dependently increased the levels of acetylated H3K9, H3K18, and H3K56 in both human (BxPC‐3) and murine (PANC‐02) pancreatic cancer cells.^405^ Moreover, **48a** dose‐dependently impaired the migration of three human pancreatic cell lines (BxPC‐3, L3.6PL, and SW1990) and the murine cell line PANC‐02, while not exerting any cytotoxic effect at 100 μM. Compound **48a** was also effective in reducing liver metastatic nodules when tested in two mouse xenograft models, one obtained using mouse‐derived PANC‐02 pancreatic cancer cells and another one constructed from human‐derived L3.6PL pancreatic cancer cells at doses of 3, 10, and 30 mg/kg administered intraperitoneally. Furthermore, the levels of acetylated H3K9, H3K18, and H3K56 were shown to increase in the L3.6PL mouse xenograft following treatment with **48a**.^405^

**Figure 15:**
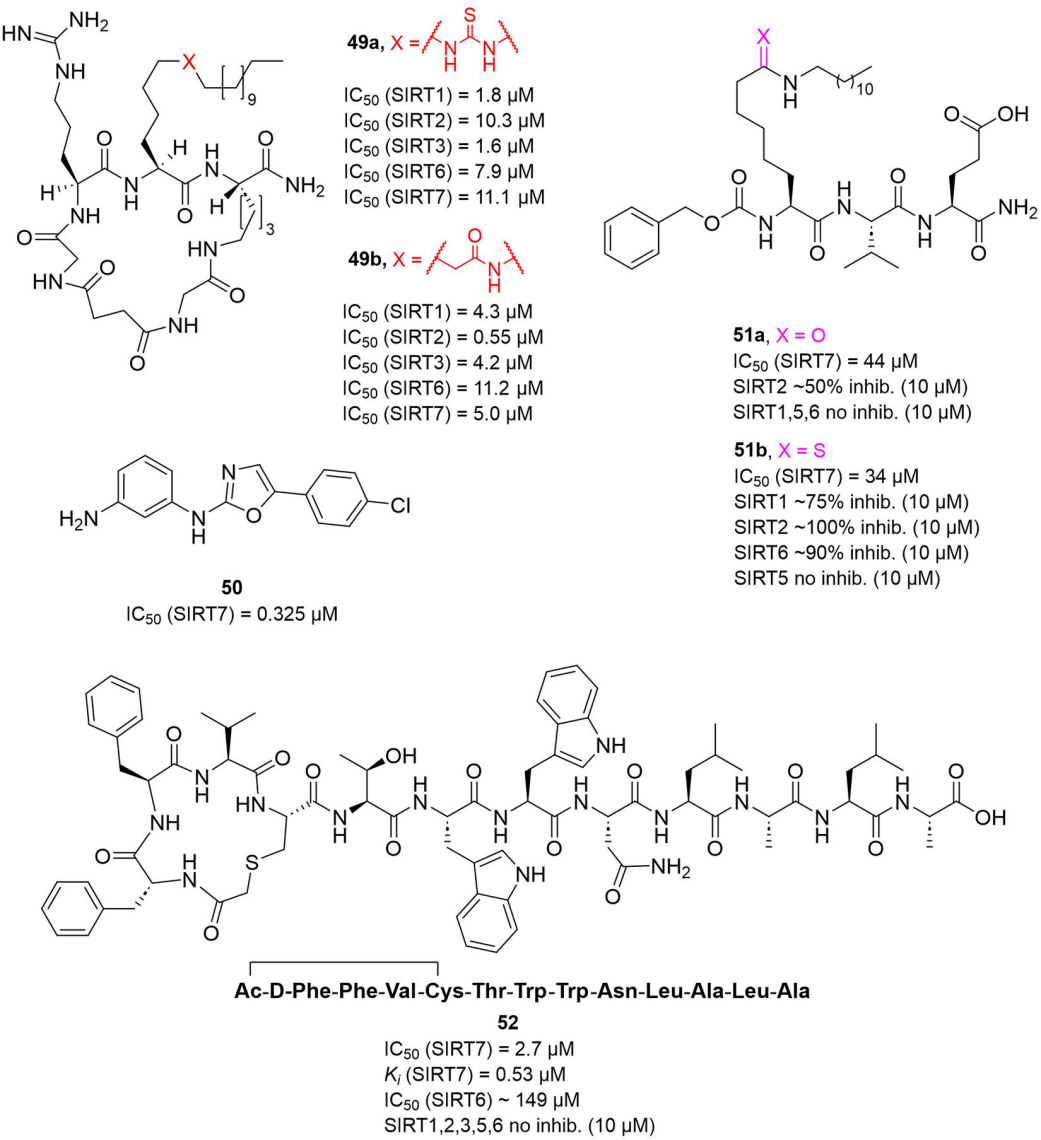
Structures and enzymatic activities of SIRT7i **49a,b**, **50**, **51a,b**, and **52**. [Color figure can be viewed at wileyonlinelibrary.com]

In 2018, Li et al. described two cyclic tripeptides (**49a,b**) as low‐micromolar SIRT7i. In compound **49a** the Lys residue forms a thiourea while in **49b** the *ε*‐amino group is replaced by a methylene group, thereby leading to a carboxamide derivative (Figure 15). The molecules possess IC_50_ values against tRNA activated SIRT7 of 11.1 and 5.0 μM, respectively. However, they are nonselective over other SIRT isoforms, except for SIRT5, and preferentially target SIRT1, 3 (**49a**) or SIRT2 (**49b**), with IC_50_ values in the (sub)micromolar range as shown in Figure 13. No cell‐based assays were performed for these inhibitors.^406^

The oxazole derivative ID:97491 (**50**, Figure 15) is a potent SIRT7i (IC_50_ = 0.325 μM),^407^ though its activity toward other SIRT isoforms was not evaluated. Compound **50** was tested in MES‐SA human uterine sarcoma cells where it augmented p53 acetylation (at Lys373/Lys382) and phosphorylation (at S392) and facilitated caspase‐induced apoptosis by raising the Bax and p21 levels. In line with this, **50** decreased cell proliferation in a dose‐dependent manner while not inducing cytotoxicity. Compound **50** also dose‐dependently suppressed tumor growth in uterine sarcoma xenograft mouse models.^407^ Given that p53 is a substrate of SIRT1 and that isoform selectivity was not assessed for compound **50**, it is not possible to exclude the possibility that the observed cellular effects are the result of SIRT1 inhibition.

The Olsen group recently developed a series of SIRT7i based on the sequence of decanoylated H3_15‐18_ and H3_33‐36_ peptides containing H3K18 and H3K36, which are known SIRT7 substrates.^35^ This led to peptidomimetics **51a,b**, which inhibited SIRT7 dedecanoylase activity with IC_50_ values of 44 and 34 μM, respectively (Figure 15). Nevertheless, both compounds lack in selectivity. Compound **51a** exhibited ~50% SIRT2 inhibition at 10 μM, while little or no inhibition was observed for SIRT1, 5, and 6. Compound **51b** showed ~75% SIRT1 inhibition, ~100% SIRT2, and ~90% SIRT6 inhibition at 10 μM, thus being inactive only toward SIRT5. The fact that both compounds are strong SIRT2 inhibitors is not surprising given the similarities with TM (**23a**) and its analogs. The authors then identified the cyclic peptide **52** through a random nonstandard peptide integrated discovery (RaPID) screening platform. This compound exhibited an IC_50_ value of 2.7 μM, along with a *K*_*i*_ of 0.53 μM and selectivity over SIRT1‐3, 5, and 6 at 10 μM, with an estimated IC_50_ value against SIRT6 of 149 μM. The authors confirmed the target engagement in HEK293T cells through CETSA (performed at a concentration of 10 μM) and showed that **52** can augment H3K18 acetylation in the same cell line at both 1 and 10 μM, while no influence was observed for the other SIRT7 substrates H3K27 and H3K36 at the same concentrations.^35^

The most relevant SIRT6/7 inhibitors identified so far are shown in Table 3.

## 4 CONCLUSIONS AND OUTLOOK

In this review, we summarize the most relevant features of SIRT enzymes along with a description of their most relevant modulators. Over the last 20 years, the intensive efforts to generate SIRT modulators have provided critical findings on SIRT biological functions and considerable advancements in deciphering their catalytic mechanisms. Although the different isoforms are not identical, they do possess similar features, hence most of the presently available modulators are active toward multiple isoforms.

Sirtuins can reprogram energy metabolism pathways (glycolysis, gluconeogenesis, fatty acid β‐oxidation, and lipogenesis) by targeting various metabolic enzymes and transcription factors. Their manifold actions and substrates influence the development of disorders such as metabolic disease, cancer, cardiovascular pathologies, and neurodegeneration. Specifically, all SIRTs have been implicated in cancer by acting either as promoters or suppressors through the modulation of DNA damage repair, oxidative stress response, differentiation, cell cycle, and apoptosis. Due to the wide and critical catalytic activity of the whole family, sirtuins have been regarded as critical targets for the treatment of several disorders, and both SIRT activators and inhibitors have been developed over the years.

Early drug discovery initiatives resulted in a variety of effective SIRT1 activators that have been tested in Phase I and II clinical trials. The discovery of resveratrol (**1**) as a SIRT1a and its evaluation in multiple clinical trials^245^, ^249^, ^252^ prompted the development of STACs (**2a‐h**), among which SRT2104 (**2c**)^254^ showed promising outcomes in Phase I/II clinical trials,^257^, ^258^, ^259^, ^260^ such as in psoriasis patients. It should be noted that studies on STACs have raised different controversies over the past years. Indeed, their activation of SIRT1 was initially attributed to the presence of a hydrophobic fluorophore in the substrate peptide. Further assessment finally indicated that the fluorophore reproduces the features of endogenous substrates necessary for activation and analysis of different peptides confirmed this notion, suggesting that STACs are able to activate SIRT1 in vivo only when it deacetylates certain substrates. These findings, along with the promising (although preliminary) results obtained with **2c** in clinical trials, strongly support ongoing research toward SIRT1 activators. Importantly, the mini‐hSIRT1/**2h** co‐crystal structure represents a key contribution to the field since it uncovered key information on activators' binding mode.^268^ Overall, it would be necessary to follow two paths: one aimed at developing analogs of **2c** endowed with better pharmacokinetic or physicochemical properties to ameliorate oral administration or topical use (as in psoriasis); another research line should be focused on the development of new STACs with different structures and activation modes, independent from the substrate of choice.

Given the positive implications of other sirtuins in health and lifespan, recent efforts have led to the development of SIRT3, SIRT5, and SIRT6 activators. These include the amiodarone derivative **3c**, a submicromolar SIRT3a,^270^ the DHP‐based SIRT3a **5d** ^276^ and SIRT5a **5g**,^278^ and the SIRT6a **7a,c**.^198^, ^285^, ^291^ All these molecules exhibited selective activation of their targets accompanied by potent cellular and in vivo activity. Hence, they represent promising lead compounds for future drug discovery campaigns. Compound **3c** represents a milestone in SIRT3a discovery but necessitates further structural modifications to reduce its pulmonary adverse effects resulting from its structural similarity to the parent drug amiodarone (**3a**). Compounds **5d** and **5g** represents a great example of how even small modifications can lead to a shift in isoform selectivity. Although potent, **5g** may still be optimized to yield submicromolar SIRT5a, hence future studies are necessary to investigate its SAR and develop more potent molecules. The MDL series of SIRT6a represents a great advancement in the field, although compound **7b** has generated controversies related to its binding mode to SIRT6. As mentioned above, this may be due to different conditions employed in the crystallization experiments such as the presence or not of the substrate in the crystallization mixture. Indeed, solving the co‐crystal structure of SIRT6/**7c** in the presence or absence of the substrate would probably help to get a better idea of the binding mode of these molecules, which, although potent, still need optimization to reach submicromolar to nanomolar EC_50_ values.

The development of compound **9a** and the release of SIRT1/**9b** co‐crystal structure definitely represents an important milestone for SIRTi drug discovery. Although the selectivity data over SIRT2 are contrasting (2–159‐fold, depending on the study),^293^, ^294^, ^296^, ^297^ **9a** still represents one of the most promising SIRT1i and, given its good drug‐like properties it was brought to clinical trials where it was shown to be safe^308^ and effective in early HD patients.^309^ Concerning the experiments in cancer cells, the correlation between SIRT1 inhibition and the reported effects should be interpreted cautiously, since the doses employed (e.g., 50 µM or even more) in some studies may have also determined SIRT2 inhibition. Furthermore, contrasting results have been reported when **9a** was assessed in cellular and mouse models of pancreatic cancer.^307^ Consequently, novel derivatives endowed with higher SIRT1 selectivity, along with improved potency, might lead to more promising outcomes, especially in cancer.

The discovery of SirReal2 (**24a**)^361^ set the ground for the design and synthesis of a diverse array of SIRT2‐selective inhibitors and probes that have been used to elucidate SIRT2 biology. Moreover, the SIRT2‐**24a** co‐crystal structure enabled the structure‐based campaign that led to compound **25a** which shows great potency and selectivity against SIRT2 and exhibits anticancer activity in breast cancer cells.^370^ Similarly, FLS‐359 (**26**)^372^ is a valuable tool for studying SIRT2 biology and represents an interesting lead compound for further development. Nevertheless, more research is required to establish these compounds as potential drug candidates. These include further target engagement assays, evaluation in mouse models, and the complete assessment of their pharmacokinetic properties.

In the case of SIRT3, no cellular activity has been reported for the most potent inhibitors identified so far (**29a**,**b** and **30**),^376^, ^377^ while anticancer activity has been reported only for the amino acid derivative **31** in head and neck squamous cell carcinoma lines.^378^ Moreover, there have been efforts to develop mitochondria‐targeted SIRT3 inhibitors such as in the case of compounds **23e** and **23f**.^121^, ^360^ In both instances, the developed compounds exhibit preferential in vitro inhibition of other SIRT isoforms (SIRT2 for **23e** and SIRT1 for **23f**) and further research is required to prove that the observed cellular effects are solely due to the inhibition of SIRT3. As for SIRT4, the recent discovery of the low‐micromolar inhibitors **33a, b** represents a significant advancement in understanding the biology and the clinical implications of this enzyme.^380^ In the case of SIRT5, the most potent and cellularly active molecules described to date are the peptide derivatives **39b‐d** and **39g** ^166^, ^392^, ^394^ and **40b,c** ^395^ which, used as prodrugs, displayed favorable anticancer effects in AML and breast cancer cellular and mouse models. Nonetheless, further research would be necessary to find small molecule derivatives endowed with increased drug‐like properties. To this end, the small molecules **36b** and **37c** represent great starting points given their submicromolar inhibition of SIRT5 and good isoform selectivity. Obtaining the co‐crystal structures of SIRT5 bound to one of the compounds and assessing their cellular activity and target engagement would undoubtedly provide valuable insights, as it would identify the most appropriate lead compound from which to launch subsequent drug discovery programs.

An interesting SIRT6i is **43b**, exhibiting selective low‐micromolar SIRT6 inhibition and reduced blood glucose levels in a diabetic mouse model.^400^ Its basic structure is prone to modifications that may improve compound activity. Moreover, the recently reported JYQ‐42 (**45**) seems an ideal candidate for further development given its low‐micromolar SIRT6 inhibition, isoform selectivity, and cellular activity.^403^ Moreover, the authors disclosed a novel interaction site for SIRT6, proposing the presence of an allosteric pocket. Subsequent research uncovered low‐micromolar SIRT6 inhibitors **48a** and **48b**, and the relative co‐crystal structure of SIRT6 in complex with **48b**, which was shown to bind to the same binding pocket of SIRT6 activator **6a**. Notably, the absence of interaction with a key Pro62 and the presence of a hydrogen bond with Asp116 seem to switch the activity of the compound as an inhibitor rather than activator.^405^ Furthermore, **48a** exhibited promising anticancer activity in both in vitro and in vivo pancreatic cancer models, thereby representing a promising candidate for further optimization.

Finally, the most potent small molecule SIRT7 inhibitor identified to date (**50**) has not been tested for its selectivity,^407^ while the only compound with demonstrated selectivity and target engagement is a cyclic dodecapeptide (**52**),^35^ which is still far from being a drug‐like molecule. Therefore, it is quite clear that the field of SIRT7 inhibition is still in its early stages.

Overall, although many modulators have been reported to date, there are still some underdeveloped areas that need further research, like in the case of SIRT4 and SIRT7 modulation. To this end, the integration of functional data and target engagement assays with biochemical, biophysical, and structural information^408^, ^409^, ^410^, ^411^ will enable the development of new and more specific modulators. For instance, the great advances in cryo‐electron microscopy (cryo‐EM) have significantly increased the possibility of achieving high‐resolution structures of small proteins,^412^, ^413^ thereby offering an alternative method in cases where crystallography has failed. In addition, the recent release of AlphaFold,^414^, ^415^ that has allowed the accurate prediction of hundreds of thousands of proteins, could accelerate the drug discovery programs targeting particularly challenging proteins such as sirtuins.

The potential therapeutic success of SIRT activators and inhibitors will require a deeper knowledge of the sirtuin role in every disease state, particularly cancer, in which SIRT functions are highly context‐dependent. Although there are still some issues, such as imbalanced development of sirtuin modulators and the presence of only few promising compounds in clinical trials, the development of isoform specific SIRT modulators is a topic that certainly deserves further investigation efforts as it has good chances to ultimately lead to the next‐generation drugs.

## Supporting information

Supporting information.

## Data Availability

Data sharing is not applicable to this article as no new data were created or analyzed in this study.
