# Supplementary material for: Activation and inhibition of sirtuins: From bench to bedside
Source: Med Res Rev. 2024 Aug 31;45(2):484–560. doi: 10.1002/med.22076 (PMC11796339; doi:10.1002/med.22076)
Supplement: Supplementary file 1 — Supporting information. [file MED-45-484-s001.docx]

Please complete the following regarding your submission and upload this file at the time of submission as “Supplementary Information – Not for Review.”

1. **Title:** Activation and Inhibition of Sirtuins: from Bench to Bedside
2. **Author(s):** Francesco Fiorentino, Antonello Mai, Dante Rotili
3. **A link to the corresponding author’s published papers, such as ORCID or lab website:** https://orcid.org/0000-0002-8428-8763
4. **Keywords, separated by a semicolon:** Sirtuins; Protein lysine deacylation; Cancer; Metabolism; Drug discovery.
5. **Brief justification for this review:** *Why is this particular review timely and distinct from other reviews in the field?*

Through this review, we aim to provide critical insights into sirtuin chemistry and pharmacology along with a detailed discussion of sirtuin structures, enzymatic activities, and biological functions. The scientific community will benefit from a timely outline of sirtuin modulators that includes their modes of action, structure-activity relationships, pharmacological effects, and potential clinical applications, along with a critical perspective on the state-of-the-art.

To date, there are no updated reviews focusing on sirtuins’ biology and providing at the same time an accurate and systematic description of their activators and inhibitors. Indeed, two recent reviews on sirtuins (doi: 10.3389/fphar.2021.735044 and doi: 10.1080/17460441.2021.1915980) only describe few modulators without providing enough biological context and a discussion on structure-activity relationships, while a recent short review by our group (doi: 10.4155/fmc-2022-0031) focuses on selected compounds and only provides little biological background information. Finally, another review (doi: 10.1146/annurev-biochem-082520-125411) describes the biochemistry of sirtuins, along their interaction partners, however it does not provide a description of sirtuin involvement in pathological contexts and their modulation via small molecules/peptides. Hence, no review has provided an integrated analysis of both the biological properties and activity modulation of sirtuins, and we therefore aim to fill this gap*.*

1. **Brief summary (~5 sentences) of the conclusion / take-home message of your manuscript:**

Over the last 20 years, intensive efforts to generate sirtuin modulators have yielded critical findings on sirtuin biological functions as well as significant advances in deciphering their catalytic mechanisms. Indeed, many sirtuin modulators have been reported to date, although there are some unexplored areas that require further investigation. These questions may be addressed using a combination of biochemical, biophysical, structural, and functional data along with computational approaches.

Furthermore, the therapeutic efficacy of SIRT activators and inhibitors will require a better understanding of the role of sirtuins in every disease state, especially cancer, where sirtuin actions are extremely context-dependent. As a result, while there are still some issues, such as imbalanced development of sirtuin modulators and only a few promising compounds in clinical trials, we believe that, given the important roles of sirtuins in metabolic processes and tumorigenesis, investigating potential modulators is a priority topic in drug discovery that might lead to next-generation drugs.

1. **Years of literature covered?** 2001-present
2. **List of the most related publications by the author(s**)**:**
3. Fiorentino F, Mautone N, Menna M, D’Acunzo F, Mai A, **Rotili D**. Sirtuin modulators: past, present, and future perspectives. *Future Med. Chem.* 2022; online ahead of print. doi: [10.4155/fmc-2022-0031](https://doi.org/10.4155/fmc-2022-0031)
4. Taurone S, De Ponte C, **Rotili D**, De Santis E, Mai A, Fiorentino F, Scarpa S, Artico M, Micera A. Biochemical Functions and Clinical Characterizations of the Sirtuins in Diabetes-Induced Retinal Pathologies. *Int. J. Mol. Sci.* 2022; 23(7):4048. doi: [10.3390/ijms23074048](https://doi.org/10.3390/ijms23074048)
5. Hu T, Shukla SK, Vernucci E, He C, Wang D, King RJ, Jha K, Siddhanta K, Mullen NJ, Attri KS, Murthy D, Chaika NV, Thakur R, Mulder SE, Pacheco CG, Fu X, High RR, Yu F, Lazenby A, Steegborn C, Lan P, Mehla K, **Rotili D**, Chaudhary S, Valente S, Tafani M, Mai A, Auwerx J, Verdin E, Tuveson D, Singh PK.  [Metabolic Rewiring by Loss of Sirt5 Promotes Kras-Induced Pancreatic Cancer Progression.](https://pubmed.ncbi.nlm.nih.gov/34245764/) *Gastroenterology* 2021; 161(5):1584-1600. doi: [10.1053/j.gastro.2021.06.045](https://doi.org/10.1053/j.gastro.2021.06.045).
6. Fiorentino F, Mai A, **Rotili D.** Emerging Therapeutic Potential of SIRT6 Modulators. *J. Med. Chem.* 2021; 64(14):9732-9758. doi: [10.1021/acs.jmedchem.1c00601](https://doi.org/10.1021/acs.jmedchem.1c00601).
7. Molinari F, Feraco A, Mirabilii S, Saladini S, Sansone L, Vernucci E, Tomaselli G, Marzolla V, **Rotili D**, Russo MA, Ricciardi MR, Tafuri A, Mai A, Caprio M, Tafani M, Armani A.
   SIRT5 Inhibition Induces Brown Fat-Like Phenotype in 3T3-L1 Preadipocytes. *Cells* 2021; 10(5):1126. doi: [10.3390/cells10051126](https://doi.org/10.3390/cells10051126).
8. Fiorentino F, Carafa V, Favale G, Altucci L, Mai A, **Rotili D.** The Two-Faced Role of SIRT6 in Cancer. *Cancers* 2021; 13(5):1156. doi: [10.3390/cancers13051156](https://doi.org/10.3390/cancers13051156).
9. Fioravanti R, Mautone N, Rovere A,**Rotili D**,* Mai A. Targeting histone acetylation/deacetylation in parasites: an update (2017-2020). *Curr. Opin. Chem. Biol.* 2020; 57:65-74. doi: [10.1016/j.cbpa.2020.05.008](https://doi.org/10.1016/j.cbpa.2020.05.008). *co-corresponding author
10. Carafa V, Russo R, Della Torre L, Cuomo F, Dell'Aversana C, Sarno F, Sgueglia G, Di Donato M,**Rotili D**, Mai A, Nebbioso A, Cobellis G, Chambery A, Altucci L. The Pan-Sirtuin Inhibitor MC2494 Regulates Mitochondrial Function in a Leukemia Cell Line. *Front. Oncol.* 2020; 10:820. doi: [10.3389/fonc.2020.00820](https://doi.org/10.3389/fonc.2020.00820).
11. Matutino Bastos T, Botelho Pereira Soares M, Haddad Franco C, Alcântara L, Antonini L, Sabatino M, Mautone N, Holanda Freitas-Junior L, Moraes CB, Ragno R,**Rotili D**, Schenkman S, Mai A, Silvio Moretti N. Identification of Inhibitors to *Trypanosoma cruzi* Sirtuins Based on Compounds Developed to Human Enzymes. *Int. J. Mol. Sci.* 2020; 21(10):3659. doi: [10.3390/ijms21103659](https://doi.org/10.3390/ijms21103659).
12. Tomaselli D, Steegborn C, Mai A,**Rotili D.** Sirt4: A Multifaceted Enzyme at the Crossroads of Mitochondrial Metabolism and Cancer. *Front. Oncol.* 2020; 10:474. doi: [10.3389/fonc.2020.00474](https://doi.org/10.3389/fonc.2020.00474).
13. Mautone N, Zwergel C, Mai A,**Rotili D.** Sirtuin modulators: where are we now? A review of patents from 2015 to 2019. *Expert Opin. Ther. Pat.* 2020; 30(6):389-407. doi: [10.1080/13543776.2020.1749264](https://doi.org/10.1080/13543776.2020.1749264).
14. Carafa V, Poziello A, Della Torre L, Giovannelli P, Di Donato M, Safadeh E, Yu Z, Baldi A, Castoria G, Tomaselli D, Mai A, **Rotili D**,* Nebbioso A, Altucci L. *Int. J. Mol. Sci.* 2019; 20(22). pii: E5654. doi: [10.3390/ijms20225654](https://doi.org/10.3390/ijms20225654). *co-corresponding author
15. Monaldi D, **Rotili D**,* Lancelot J, Marek M, Wössner N, Lucidi A, Tomaselli D, Ramos-Morales E, Romier C, Pierce RJ, Mai A, Jung M. [Structure-Reactivity Relationships on Substrates and Inhibitors of the Lysine Deacylase Sirtuin 2 from *Schistosoma mansoni* (*Sm*Sirt2).](https://www.ncbi.nlm.nih.gov/pubmed/31496251) *J. Med. Chem.* 2019; 62(19):8733-8759. doi: [10.1021/acs.jmedchem.9b00638](https://pubs.acs.org/doi/10.1021/acs.jmedchem.9b00638). *co-corresponding author

1. Iachettini S, Trisciuoglio D, **Rotili D**, Lucidi A, Salvati E, Zizza P, Di Leo L, Del Bufalo D, Ciriolo MR, Leonetti C, Steegborn C, Mai A, Rizzo A, Biroccio A. Pharmacological activation of SIRT6 triggers lethal autophagy in human cancer cells. *Cell Death Dis*. 2018; 24;9(10):996. doi: [10.1038/s41419-018-1065-0](https://doi.org/10.1038/s41419-018-1065-0).
2. Carafa V, Nebbioso A, Cuomo F, **Rotili D**, Cobellis G, Bontempo P, Baldi A, Spugnini EP, Citro G, Chambery A, Russo R, Ruvo M, Ciana P, Maravigna L, Shaik J, Radaelli E, De Antonellis P, Tarantino D, Pirolli A, Ragno R, Zollo M, Stunnenberg HG, Mai A, Altucci L. RIP1-HAT1-SIRT Complex Identification and Targeting in Treatment and Prevention of Cancer. *Clin. Cancer Res*. 2018; 15;24(12):2886-2900. doi: [10.1158/1078-0432.CCR-17-3081](https://doi.org/10.1158/1078-0432.CCR-17-3081).
3. Pannek M, Simic Z, Fuszard M, Meleshin M, **Rotili D**, Mai A, Schutkowski M, Steegborn C. Crystal structures of the mitochondrial deacylase Sirtuin 4 reveal isoform-specific acyl recognition and regulation features. *Nat. Commun*. 2017; 15;8(1):1513. doi: [10.1038/s41467-017-01701-2](https://doi.org/10.1038/s41467-017-01701-2).
4. Moniot S, Forgione M, Lucidi A, Hailu GS, Nebbioso A, Carafa V, Baratta F, Altucci L, Giacché N, Passeri D, Pellicciari R, Mai A, Steegborn C, **Rotili D**. Development of 1,2,4-Oxadiazoles as Potent and Selective Inhibitors of the Human Deacetylase Sirtuin 2: Structure-Activity Relationship, X-ray Crystal Structure, and Anticancer Activity. *J. Med. Chem.* 2017; 60(6):2344-2360. doi: [10.1021/acs.jmedchem.6b01609](https://doi.org/10.1021/acs.jmedchem.6b01609).
5. You W, **Rotili D**, Li TM, Kambach C, Meleshin M, Schutkowski M, Chua KF, Mai A, Steegborn C. Structural Basis of Sirtuin 6 Activation by Synthetic Small Molecules. *Angew. Chem. Int. Ed. Engl.* 2017; 56(4):1007-1011. doi: [10.1002/anie.201610082](https://doi.org/10.1002/anie.201610082).
6. Carafa V, **Rotili D**, Forgione M, Cuomo F, Serretiello E, Hailu GS, Jarho E, Lahtela-Kakkonen M, Mai A, Altucci L. Sirtuin functions and modulation: from chemistry to the clinic. *Clin. Epigenetics*. 2016; 8:61. doi: [10.1186/s13148-016-0224-3](https://doi.org/10.1186/s13148-016-0224-3).
7. Polletta L, Vernucci E, Carnevale I, Arcangeli T, **Rotili D**, Palmerio S, Steegborn C, Nowak T, Schutkowski M, Pellegrini L, Sansone L, Villanova L, Runci A, Pucci B, Morgante E, Fini M, Mai A, Russo MA, Tafani M. SIRT5 regulation of ammonia-induced autophagy and mitophagy. *Autophagy*. 2015; 11(2):253-270. doi: [10.1080/15548627.2015.1009778](https://doi.org/10.1080/15548627.2015.1009778).
8. Scuderi C, Stecca C, Bronzuoli MR, **Rotili D**, Valente S, Mai A, Steardo L. Sirtuin modulators control reactive gliosis in an in vitro model of Alzheimer's disease. *Front. Pharmacol.* 2014; 5:89. doi: [10.3389/fphar.2014.00089](https://doi.org/10.3389/fphar.2014.00089).
9. Mellini P, Carafa V, Di Rienzo B, **Rotili D**, De Vita D, Cirilli R, Gallinella B, Provvisiero DP, Di Maro S, Novellino E, Altucci L, Mai A. Carprofen analogues as sirtuin inhibitors: enzyme and cellular studies. *ChemMedChem*. 2012; 7(11):1905-1908. doi: [10.1002/cmdc.201200318](https://doi.org/10.1002/cmdc.201200318).
10. **Rotili D**, Tarantino D, Nebbioso A, Paolini C, Huidobro C, Lara E, Mellini P, Lenoci A, Pezzi R, Botta G, Lahtela-Kakkonen M, Poso A, Steinkühler C, Gallinari P, De Maria R, Fraga M, Esteller M, Altucci L, Mai A. Discovery of salermide-related sirtuin inhibitors: binding mode studies and antiproliferative effects in cancer cells including cancer stem cells. *J. Med. Chem.* 2012; 55(24):10937-10947. doi: [10.1021/jm3011614](https://doi.org/10.1021/jm3011614).
11. **Rotili D**, Tarantino D, Carafa V, Paolini C, Schemies J, Jung M, Botta G, Di Maro S, Novellino E, Steinkühler C, De Maria R, Gallinari P, Altucci L, Mai A. Benzodeazaoxaflavins as sirtuin inhibitors with antiproliferative properties in cancer stem cells*. J. Med. Chem.* 2012; 55(18):8193-8197. doi: [10.1021/jm301115r](https://doi.org/10.1021/jm301115r).
12. **Rotili D**, Carafa V, Tarantino D, Botta G, Nebbioso A, Altucci L, Mai A. Simplification of the tetracyclic SIRT1-selective inhibitor MC2141: coumarin- and pyrimidine-based SIRT1/2 inhibitors with different selectivity profile. *Bioorg. Med. Chem.* 2011; 19(12):3659-3668. doi: [10.1016/j.bmc.2011.01.025](https://doi.org/10.1016/j.bmc.2011.01.025).
13. **Rotili D**, Tarantino D, Carafa V, Lara E, Meade S, Botta G, Nebbioso A, Schemies J, Jung M, Kazantsev AG, Esteller M, Fraga MF, Altucci L, Mai A. Identification of tri- and tetracyclic pyrimidinediones as sirtuin inhibitors. *ChemMedChem.* 2010; 5(5):674-7. doi: [10.1002/cmdc.201000030](https://doi.org/10.1002/cmdc.201000030).
14. Pasco MY, **Rotili D**, Altucci L, Farina F, Rouleau GA, Mai A, Néri C. Characterization of sirtuin inhibitors in nematodes expressing a muscular dystrophy protein reveals muscle cell and behavioral protection by specific sirtinol analogues. *J. Med. Chem*. 2010; 53(3):1407-11. doi: [10.1021/jm9013345](https://doi.org/10.1021/jm9013345).
